# Molecular Detection and Distribution of *Giardia duodenalis* and *Cryptosporidium* spp. Infections in Wild and Domestic Animals in Portugal

**DOI:** 10.1155/2023/5849842

**Published:** 2023-11-08

**Authors:** Ana M. Figueiredo, Pamela C. Köster, Alejandro Dashti, Rita T. Torres, Carlos Fonseca, Atle Mysterud, Begoña Bailo, João Carvalho, Eduardo Ferreira, Dário Hipólito, Joana Fernandes, Ana Lino, Josman D. Palmeira, Pedro Sarmento, Nuno Neves, Carlos Carrapato, Rafael Calero-Bernal, David Carmena

**Affiliations:** ^1^Department of Biology and CESAM, University of Aveiro, Campus Universitário de Santiago, 3810–193, Aveiro, Portugal; ^2^Centre for Ecological and Evolutionary Synthesis, Department of Bioscience, University of Oslo, P.O Box 1066 Blindern, NO-316 Oslo, Norway; ^3^Parasitology Reference and Research Laboratory, Spanish National Centre for Microbiology, Majadahonda 28220, Madrid, Spain; ^4^ForestWISE – Collaborative Laboratory for Integrated Forest & Fire Management, Quinta de Prados, 5001–801, Vila Real, Portugal; ^5^Veterinary Biology Unit, Faculty of Veterinary Medicine, University of Zagreb, Heinzelova 55 10000, Zagreb, Croatia; ^6^Center for Evolutionary Hologenomics, The GLOBE Institute, University of Copenhagen, Copenhagen, Denmark; ^7^Instituto da Conservação da Natureza e das Florestas, Direção Regional do Alentejo, Centro Polivalente da Casa do Lanternim, Rua D. Sancho II., n15 7750–350 Mértola, Portugal; ^8^SALUVET, Animal Health Department Faculty of Veterinary Sciences, Complutense University of Madrid, Ciudad Universitaria s/n 28040, Madrid, Spain; ^9^CIBER Infectious Diseases (CIBERINFEC), Health Institute Carlos III, 28029, Madrid, Spain

## Abstract

Enteric protozoan parasites *Giardia duodenalis*, *Cryptosporidium* spp., and, to a lesser extent, the ciliate *Balantioides coli* are responsible for severe human and animal intestinal disorders globally. However, limited information is available on the occurrence and epidemiology of these parasites in domestic, but especially wild species in Portugal. To fill this gap of knowledge, we have investigated *G. duodenalis*, *Cryptosporidium* spp., and *B. coli* occurrence, distribution, genetic diversity, and zoonotic potential by analyzing 756 fecal samples from several wild carnivores (*n* = 288), wild ungulates (*n* = 242), and domestic species (*n* = 226) collected across different areas of mainland Portugal. Overall, infection rates were 16.1% (122/756; 95% CI: 13.59–18.96) for *G. duodenalis* and 2.7% (20/756; CI: 1.62–4.06) for *Cryptosporidium* spp., while no ungulate sample analyzed yielded positive results for *B. coli. Giardia duodenalis* was found across a wide range of hosts and sampling areas, being most prevalent in the Iberian lynx (26.7%), the Iberian wolf (24.0%), and the domestic dog (23.9%). *Cryptosporidium* spp. was only identified in wild boar (8.4%), red fox (3.4%), Iberian lynx (3.3%), red deer (3.1%), and Iberian wolf (2.5%). Sequence analysis of *G. duodenalis* determined zoonotic assemblage A (subassemblage AI) in one roe deer sample, canine-specific assemblages C and D in Iberian wolf, red fox, and domestic dog, and ungulate-specific assemblage E in wild boar, sheep, cattle, and horse. Six *Cryptosporidium* species were identified: *C. scrofarum* in wild boar, *C. canis* in the Iberian wolf and red fox, *C. ubiquitum* in red deer and wild boar, *C. felis* in the Iberian lynx, and both *C. ryanae* and *C. occultus* in red deer. *Giardia duodenalis* and *Cryptosporidium* spp. coinfections were observed in 0.7% (5/756) of the samples. This is the first, most comprehensive, and largest molecular-based epidemiology study of its kind carried out in Portugal, covering a wide range of wild and domestic hosts and sampling areas. The detection of zoonotic *Cryptosporidium* spp. and *G. duodenalis* subassemblage AI demonstrates the role of wild and domestic host species in the transmission of these agents while representing a potential source of environmental contamination for other animals and humans.

## 1. Introduction

*Giardia duodenalis* (syn. *G. lamblia*, *G. intestinalis*) and *Cryptosporidium* spp. are two of the most common enteric protozoan parasites accountable for human and animal intestinal disorders worldwide [1, 2]. Near 200 million human symptomatic cases of giardiasis are reported annually [3]. Cryptosporidiosis is second only to rotavirus infection as a contributor to childhood diarrhea in poor-resource settings [4]. Acute to chronic diarrhea, abdominal pain, lack of appetite, malabsorption, and weight loss are the main clinical manifestations described for both protozoan infections [2, 5]. In children living in endemic areas, giardiasis and cryptosporidiosis are associated with growth retardation and cognitive impairment, extending their impact to life-threatening malnutrition and wasting [6, 7]. Notwithstanding, asymptomatic infections can also be frequent, depending on the parasite strain and the host's immunological and health status [8, 9]. *Cryptosporidium* spp. and, to a lesser extent, *G. duodenalis* infections are linked to decreased growth rates and, in the case of *Cryptosporidium*, increased mortality in infected livestock species, especially neonatal individuals, triggering significant economic losses to the sector [2, 10]. Although its worldwide prevalence in humans usually does not exceed 1%, *Balantioides coli* (formerly known as *Balantidium coli* and *Neobalantidium coli*) is the only ciliate with public health importance, having domestic pigs as the primary animal reservoir, even though infections in this host are mostly asymptomatic [11]. Human infections by *B. coli* have a similar clinical picture to those previously described for giardiasis and cryptosporidiosis, with the aggravating factor of triggering colitis, an inflammatory bowel disease (IBD) [12].

While *Cryptosporidium* displays a complex life cycle comprising both sexual and asexual replication stages [2], *G. duodenalis* and *B. coli* life cycles involve two developmental stages, the replicative form (trophozoite) and the transmissive and infective form (cyst). Infection occurs through the fecal–oral route, which involves the ingestion of environmentally resistant cysts (*G. duodenalis*, *B. coli*) or oocysts (*Cryptosporidium* spp.) through the consumption of contaminated food or water or direct contact with an infected animal/human host [5, 12, 13].

*Giardia duodenalis* is currently regarded as a species complex consisting of eight assemblages (from A to H) with marked differences in host specificity and range [14]. Assemblages A and B are zoonotic, infecting humans, companion animals, livestock, and wildlife. Host-specific assemblages C and D are mainly reported in canids, E in artiodactyls, F in felids, G in rodents, and H in marine mammals [15, 16]. For the *Cryptosporidium* genus, at least 46 taxonomically valid species have been described [14, 17, 18]. Even though *ca*. 95% of human cases of cryptosporidiosis reported are due to *C. hominis* and *C. parvum* infections [19], over 20 *Cryptosporidium* species have been identified in humans, including host-adapted *C. meleagridis*, *C. canis*, and *C. felis* [14, 20]. As for *B. coli*, three genotypes (A, B, and C) have been described: genotypes A and B have a broad host range, whereas genotype C has only been identified in nonhuman primates [21].

Studies reporting *G. duodenalis*, *Cryptosporidium* spp., and, to a lesser extent, *B. coli* in wildlife have continuously contributed to improving our understanding of the epidemiology, host range, and zoonotic potential of these parasites [22]. In Europe, wild carnivores and ungulates have had an uprising in recent years, increasing the contact rate with domestic animals and humans due to hunting practices, overlapped distribution areas, and consequent synanthropic behaviors [23, 24]. The spatial overlap between wild and domestic species, particularly involving free-roaming livestock herds but also companion animals, is increasing the spillover of zoonotic strains/genotypes in the wildlife–domestic–human interface, perpetuating the transmission and spreading of these parasites. In Europe, *G. duodenalis* and *Cryptosporidium* spp. have been reported in wolf *Canis lupus*, red fox *Vulpes vulpes*, and stone marten *Martes foina* with prevalence rates ranging from 5% to 44% for *G. duodenalis* and 2% to 36% for *Cryptosporidium* spp., whereas for wild ungulates (red deer *Cervus elaphus*, roe deer *Capreolus capreolus*, and wild boar *Sus scrofa*), these values ranged from 1% to 41% for *G. duodenalis* and 1% to 18% for *Cryptosporidium* (Table 1). Zoonotic assemblages A and B and canid-specific C and D were the most frequent genetic variants identified within *G. duodenalis*, while *C. parvum*, *C. canis*, *C. ubiquitum*, and *C. scrofarum* were the predominant *Cryptosporidium* species circulating in such hosts. As for *B. coli*, the only reports were in wild boar and red deer, with the detection of genotypes A and B (Table 1). *Giardia duodenalis* reports in domestic dogs include prevalence rates ranging from 2% to 100% and 1% to 10% for *Cryptosporidium* spp., while for livestock species, these values range from 8% to 100% and 1% to 100%, respectively. Also, there were numerous reports across Europe of *G. duodenalis* assemblages A–E and zoonotic (e.g., *C. parvum*, *C. canis*) and host-adapted (e.g., *C. ryanae*) *Cryptosporidium* species (Table 2).

In Portugal, data on the occurrence and molecular diversity of these three enteric parasites in wild species are limited to the report of *C. scrofarum* in wild boar [51] and *B. coli* genotypes A and B in red deer and wild boar [51, 52] (Table 1). Regarding domestic animals, *G. duodenalis* assemblages B, C, and D have been reported in dogs *Canis lupus familiaris* [86, 87] and A, B, and E in cattle *Bos taurus* [101]. *Cryptosporidium parvum* was documented in horses *Equus caballus* [181], sheep *Ovis aries* [125], and cattle [101, 123–125], as well as *C. meleagridis* and *C. andersoni* [101] (Table 2). Considering the overall low number of studies evaluating the molecular diversity of these enteric parasites carried out in Portugal, especially in the wildlife counterpart, this study aims to determine the distribution, genetic diversity, and zoonotic potential of *G. duodenalis*, *Cryptosporidium* spp., and *B. coli* in wild and domestic animal species across different areas of mainland Portugal.

## 2. Materials and Methods

### 2.1. Study Area and Sampling Collection

**Figure 1:**
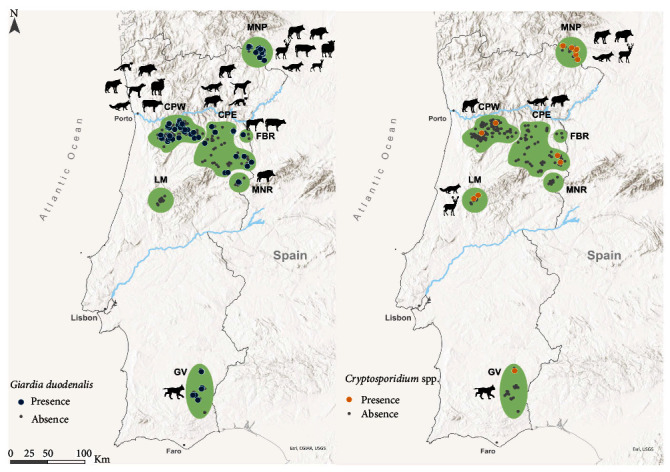
Map of mainland Portugal showing the seven study areas and the geographical distribution of *G. duodenalis* and *Cryptosporidium* spp. detected in wildlife species and domestic animals.

This study was carried out in seven distinct areas of mainland Portugal (Figure 1), reflecting contrasting environmental and climate conditions and differences in their species' community, covering European Union's Natura 2000 Network sites (https://ec.europa.eu/environment/nature/natura2000/index_en.htm). Montesinho Natural Park (MNP), Central Portugal West (CPW), and Central Portugal East (CPE) are characterized by mountainous landscapes and a continental Mediterranean climate, even though CPW exhibits a strong Atlantic influence. The three areas comprise livestock herds raised under the traditional extensive grazing system and a large diversity of wild species (e.g., the Iberian wolf, *Canis lupus signatus* and roe deer). The Faia Brava Reserve (FBR) is a privately protected enclosed area encompassing semiwild herbivores (cattle and horses), co-occurring with other wildlife species. The Guadiana Valley (GV) is situated in the southern region of Portugal and features a continental Mediterranean climate and low-altitude mountains. This area is home to a variety of wild species, free-roaming livestock herds, and the recently reintroduced apex predator, the Iberian lynx (*Lynx pardinus*). Lousã Mountains (LM) and Malcata Nature Reserve (MNR) are both characterized by a Mediterranean climate, presenting a wide variety of wild ungulate and mesocarnivore species (e.g., red deer, the red fox), even though no apex predator and free-roaming livestock species overlap their territories.

### 2.2. Sampling Collection

Between 2017 and 2021, fresh fecal samples from a total of 12 mammal species (wild and domestic), which included wild carnivores (Iberian wolf, Iberian lynx, red fox, and stone marten), wild ungulates (red deer, roe deer, and wild boar), livestock species (cattle, horse, goat *Capra hircus*, and sheep), and domestic/feral dog, were collected in the prospected study areas (Figure 1). A total of 756 individual fecal specimens were sampled from wild carnivore (38.1%, 288/756), wild ungulate (32.0%, 242/756), and domestic (29.9%, 226/756, including livestock and domestic/feral dogs) species. Samples were opportunistically collected from (i) legally hunted animals, (ii) routine checkups/live-capture operations, (iii) free-roaming livestock herds, and (iv) transects or scats trails distributed across the different study areas. During field necropsies of hunted red deer and wild boar specimens and during routine checkups/live capture procedures of Iberian lynx individuals, fecal samples were directly collected from the animal's rectum. Fecal samples from the Iberian lynx were obtained by compressing the intestinal tract of anesthetized individuals captured in the wild in the scope of ongoing projects. Concerning the remaining wild and domestic species, sampling collection was carried out whenever an animal was observed defecating or directly from the ground. For the latter case, samples were identified by experienced and field-trained personnel based on their morphology (e.g., content, size, shape) and deposition site. To reduce misleading identifications, Iberian wolf and domestic dog feces were genetically confirmed [191], together with a limited number of red fox (*n* = 49) and stone marten (*n* = 2) samples [192], as a regular procedure of ongoing monitoring projects of these species. Fecal samples were placed into 50 mL Corning-Falcon® containing 95% ethanol for preservation and transportation purposes and stored at −18°C for subsequent DNA extraction. The period between sample collection and DNA extraction varied from 3 to 24 months in retrospective samples from monitoring projects and a maximum of 5 months in prospective samples.

### 2.3. DNA Extraction and Purification

Genomic DNA was isolated from about 200 mg of feces using the QIAamp® Fast DNA Stool Mini Kit (QIAGEN, Hilden, Germany) following the manufacturer's instructions at the Department of Biology & CESAM, University of Aveiro (Aveiro, Portugal) facilities. Extracted and purified DNA samples were eluted in 200 *μ*l of polymerase chain reaction (PCR)-grade water or buffer ATE provided by the kit and sent to the Spanish National Centre for Microbiology (Majadahonda, Spain) for downstream molecular analyses.

### 2.4. Molecular Detection and Characterization of *Giardia duodenalis*, *Cryptosporidium* spp., and *Balantioides coli*

For the identification of *G. duodenalis*, a real-time PCR (qPCR) method was setup to amplify a 62-bp fragment of the small subunit of the rRNA (*ssu* RNA) gene of the parasite [193]. Samples that yielded cycle threshold (*C*_T_) values <35 in qPCR were then analyzed through a nested PCR, used to amplify a 300-bp fragment of the *ssu* RNA gene [194] to assess *G. duodenalis* molecular diversity at the assemblage level. Samples that yielded qPCR *C*_T_ values <32 were additionally assessed using a sequence-based multilocus genotyping (MLST) scheme targeting the genes encoding for the glutamate dehydrogenase (*gdh*), *β*-giardin (*bg*), and triose phosphate isomerase (*tpi*) proteins to assess *G. duodenalis* molecular diversity at the subassemblage level. A 432-bp fragment of the *gdh* gene was amplified using a seminested PCR [195], while 511- and 530-bp fragments of the *bg* and *tpi* genes, respectively, were amplified through nested PCRs [55, 196].

*Cryptosporidium* spp. presence was investigated using a nested PCR protocol, amplifying a 587-bp fragment of the *ssu* RNA gene of the parasite [197]. Subtyping tools based on the amplification of partial sequences of the 60-kDa glycoprotein (*gp60*) gene were used to ascertain intraspecies genetic diversity in samples that tested positive for *C. canis* [198], *C. felis* [199], *C. ryanae* [200], and *C. ubiquitum* [201] by *ssu*-PCR.

*B. coli* occurrence in wild and domestic ungulates (as it does not naturally infect strict carnivores) was determined by a direct PCR assay targeting the ITS1-5.8s-rRNA-ITS2 region and the last 117 bp (3′ end) of the *ssu*-rRNA sequence of this ciliate, as previously described [21].

Detailed information on the PCR cycling conditions and oligonucleotides used for molecular identification and/or characterization of the abovementioned parasites can be found in *Supplementary 1* and *Supplementary 2*, respectively. The previously described PCR protocols were conducted on a 2720 Thermal Cycler (Applied Biosystems). Reaction mixes included 2.5 units of MyTAQ^TM^ DNA polymerase (Bioline GmbH, Luckenwalde, Germany) and 5–10 *μ*l 5× MyTAQ^TM^ Reaction Buffer containing 5 mM deoxynucleotide triphosphates and 15 mM MgCl_2_. Negative and positive controls were included in all PCR runs. PCR amplicons obtained were examined on 1.5% D5 agarose gel stained with Pronasafe (Conda, Madrid, Spain) and sized using a 100-bp DNA ladder (Boehringer Mannheim GmbH, Mannheim, Germany).

### 2.5. Sequence Analysis

Positive PCR products with the expected size were directly sequenced in both directions with the corresponding internal primer pair (see *Supplementary 2*) in 10 *μ*l reactions using BigDye^TM^ chemistries and an ABI 3730xl sequencer analyser (Applied Biosystems, Foster City, CA). Raw sequences were examined with the Chromas Lite version 2.1 software (http://chromaslite.software.informer.com/2.1) to generate consensus sequences. The BLAST tool (http://blast.ncbi.nlm.nih.gov/Blast.cgi) was used to compare the newly generated sequences with reference sequences deposited at the National Center for Biotechnology Information (NCBI) GenBank database.

### 2.6. Statistical Analysis

Parasite prevalence was estimated using a binomial test in R software [202], establishing confidence limits with 95% confidence intervals (CI). A *χ*^2^ test, using the chisq.test function, was used to compare parasite prevalence between hosts (wild carnivores, wild ungulates, and domestic animals) and study areas. A median-joining haplotype network [203] was constructed using PopART 1.7 (https://popart.maths.otago.ac.nz; Leigh and Bryant [204]) using the resulting *G. duodenalis* and *Cryptosporidium* spp. sequences from *gdh*, *bg*, and *ssu*. For the network construction, all positions containing gaps (indels) or ambiguities (R, Y, W, S) were disregarded, as the algorithm cannot handle those mutations. For the network's representation, we considered the sampling area of each identified assemblage/genotype and its frequency.

## 3. Results

### 3.1. Prevalence of *Giardia duodenalis* and *Cryptosporidium* spp.

From a total of 756 analyzed fecal samples, 122 (16.1%, 95% CI: 13.59–18.96) were infected with *G. duodenalis* and 20 (2.7%, 95% CI: 1.62–4.06) with *Cryptosporidium* spp. (Table 3). None of the wild and domestic ungulate samples (*n* = 422) assessed for *B. coli* yielded positive results for this parasite.

*Giardia duodenalis* was detected across all the examined species, except for the domestic goat, and sampling areas, apart from the LM area. The occurrence of the protozoan varied across the sampled groups of animals (*χ*^2^ (2, *n* = 756) = 9.779, *P*=0.008), with the highest prevalence of *G. duodenalis* found in the Iberian lynx (26.7%, 8/30), followed by the Iberian wolf (25.6%, 31/121) and the domestic dog (23.9%, 11/46). Regarding *G. duodenalis* geographic distribution, its occurrence varied among the seven sampled study areas (*χ*^2^ (6, *n* = 756) = 19.81, *P*=0.003), and the GV (26.7%, 8/30), MNP (20.5%, 44/215), and CPW (19.3%, 48/249) were the areas where this protozoan was mainly detected (Table 3). *Cryptosporidium* spp. was only detected in wild boar (8.4%, 9/107), red fox (3.4%, 4/118), Iberian lynx (3.3%, 1/30), red deer (3.1%, 3/96), and Iberian wolf (2.5%, 3/121), demonstrating the significant differences on the occurrence of this parasite across host species (*χ*^2^ (2, *n* = 756) = 10.661, *P*=0.005). Although this protozoan was not detected in FBR and MNR sampling areas, its occurrence varied among the remaining sampled locations (*χ*^2^ (6, *n* = 756) = 14.971, *P*=0.002) (Table 3).

### 3.2. Molecular Diversity

**Figure 2:**
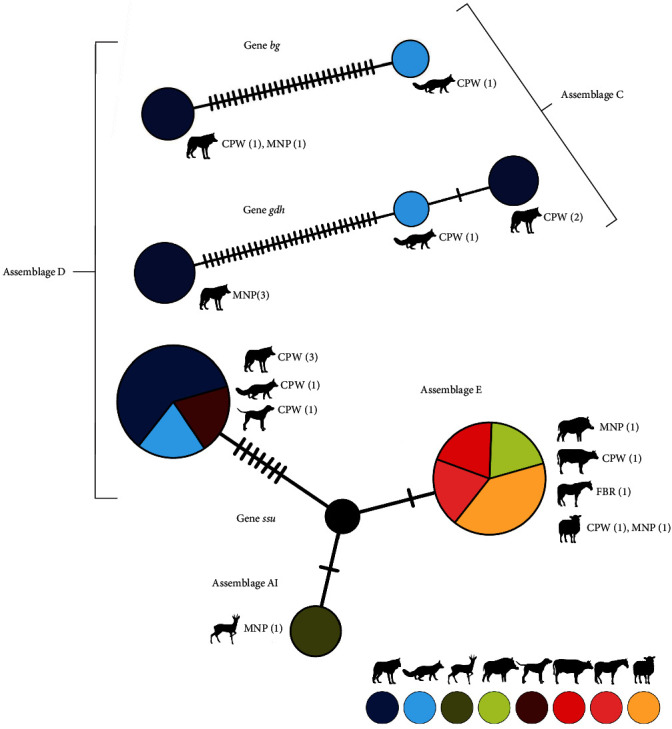
Median-joining haplotype networks constructed in PopART. *Giardia duodenalis* assemblages are represented by circles, being the sizes proportional to the number of individuals where a given assemblage was sampled. Number of mutations among assemblages is represented by the number of slashes. Different colors represent the different wild and domestic hosts. Study areas where assemblages were detected—Montesinho Natural Park (MNP), Central Portugal West (CPW), and Faia Brava Reserve (FBR)—are also indicated.

*Giardia duodenalis* qPCR-positive samples generated *C*_T_ values that ranged from 22.6 to 39.8 (median: 33.9; SD: 3.5). Only samples with *C*_T_ values ≤35 (*n* = 63) were subsequently genotyped at the *ssu*-rRNA locus for assemblage identification. Overall, four *G. duodenalis* assemblages were identified in the investigated host species based on the information retrieved for one or more of the four genetic markers (*ssu*-rRNA, *gdh*, *bg*, and *tpi*) used for genotyping purposes. These include zoonotic assemblage A (5%, 1/20), canine-adapted assemblages C (20%, 4/20) and D (50%, 10/20), and ungulate-adapted assemblage E (25%, 5/20). Nucleotide sequence analysis at the *ssu*-rRNA locus allowed assemblage identification in 55% of the species (11/20), including assemblage A, subassemblage AI (*n* = 1), found in a roe deer sample, assemblage D (*n* = 5), found in samples belonging to Iberian wolf, red fox, and dog specimens, and ungulate-specific assemblage E (*n* = 5), found in wild boar and livestock species (Table 4, Figure 2). For assemblage confirmation and subassemblage identification, samples with *C*_T_ values ≤32 (*n* = 28) were reassessed at the *gdh*, *bg*, and *tpi* loci, allowing the identification of canine-specific assemblages C (*n* = 4) and D (*n* = 5) in Iberian wolf and red fox samples. Additionally, two Iberian wolves carried mixed infections involving assemblages C (detected at the *gdh* locus) and D (detected at the *ssu*-rRNA locus), and one red fox displayed a mixed infection by assemblages C (detected at the *gdh* and *bg* loci) and D (detected at the *ssu*-rRNA locus). MNP and CPW were the only sampling areas where it was possible to determine *Giardia* assemblages (A, C, D, and E), except for one horse sample from FBR, where assemblage E was possible to identify (Table 4, Figure 2).

**Figure 3:**
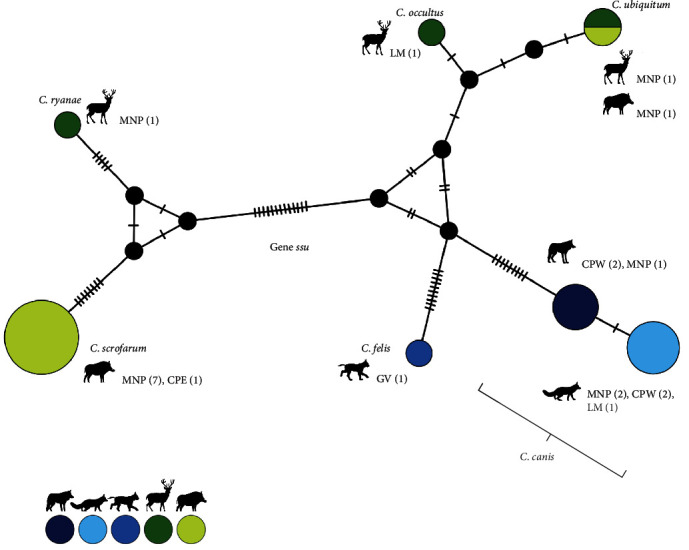
Median-joining haplotype network constructed in PopART. *Cryptosporidium* spp. genotypes are represented by circles, being the sizes proportional to the number of individuals where a given *Cryptosporidium* genotype was sampled. Number of mutations among genotypes is represented by the number of slashes. Different colors represent the different wild and domestic hosts. Study areas where genotypes were detected—Montesinho Natural Park (MNP), Central Portugal West (CPW), Central Portugal East (CPE), Lousã Mountains (LM), and Guadiana Valley (GV)—are also indicated.

Six *Cryptosporidium* species were identified: *C. scrofarum* (40.0%, 8/20), *C. canis* (35.0%, 7/20), *C. ubiquitum* (10.0%, 2/20), *C. felis* (5.0%, 1/20), *C. ryanae* (5.0%, 1/20), and *C. occultus* (5.0%, 1/20) (Table 4Figure 3). *Cryptosporidium scrofarum* was exclusively found in wild boar, while *C. canis* was found in both the Iberian wolf (*n* = 3) and red fox (*n* = 4). *Cryptosporidium ubiquitum* was found in red deer (*n* = 1) and wild boar (*n* = 1). A single sample of red deer amplified *C. ryanae* and another *C. occultus*, while *C. felis* was identified on an Iberian lynx sample. *Cryptosporidium canis* was found across four sampling areas (MNP, CPW, CPE, and LM), while *C. scrofarum* was only detected in MNP and CPE. The two positive samples for *C. ubiquitum* were found in the same area (MNP) (Figure 3). Positive samples for *C. canis*, *C. ubiquitum*, *C. ryanae*, and *C. felis* could not be further genotyped at the *gp60* gene.

Coinfections with *G. duodenalis* and *Cryptosporidium* spp. were found in five specimens (0.7%, 5/756) of the analyzed samples, belonging to two Iberian wolf samples (*G. duodenalis* assemblage D + *C. canis* and *G. duodenalis + C. canis*), one red fox (*G. duodenalis* + *C. canis*), one Iberian lynx (*G. duodenalis* + *C. felis*), and one red deer (*G. duodenalis* + *C. ubiquitum*) sample. The full dataset of this study showing sampling, epidemiological, diagnostic, and molecular data can be found in *Supplementary 3*. The sequences obtained in this study were deposited in the GenBank database under accession numbers OQ818646–OQ818654 and OQ818103–OQ818108 (*G. duodenalis*), OQ818655–OQ818656 (*C. canis*), OQ818657 (*C. felis*), OQ818658 (*C. ryanae*), OQ818659–OQ818660 (*C. scrofarum*), OQ818661 (*C. occultus*), and OQ818662–OQ818663 (*C. ubiquitum*).

## 4. Discussion

As *G. duodenalis* and *Cryptosporidium* spp. have become major sources of enteric parasitic diseases worldwide, it is paramount to recognize the role played by domestic and wild animal reservoirs in the maintenance and spread of these protozoan pathogens of public and veterinary health relevance. This study is the first molecular-based survey ever carried out in Portugal to assess *G. duodenalis* and *Cryptosporidium* spp. occurrence, distribution, molecular diversity, and zoonotic potential in wild and domestic host species. For the first time, we were able (i) to genotype *Cryptosporidium* spp. in the Iberian wolf and the Iberian lynx, (ii) to detect *G. duodenalis* in the Iberian lynx, and (iii) to successfully genotype *G. duodenalis* in the Iberian wolf. In addition, we investigated the occurrence and host distribution of *B. coli*, a ciliate protozoan parasite whose epidemiology is poorly known in Portugal and was not detected in any of the species and areas screened here. This study comes as a follow-up to the one already developed for the microsporidia *Enterocytozoon bieneusi*, using the same range of host species and sampling areas [206].

### 4.1. Prevalence of *Giardia duodenalis* in Wild and Domestic Species

In our survey, *G. duodenalis* was the most prevalent enteric parasite found (16.1%), with the highest prevalence documented in the Iberian lynx (26.7%), followed by the Iberian wolf (25.6%) and the domestic dog (23.9%) (Table 3). In previous molecular studies carried out in Portugal, *G. duodenalis* was reported at prevalence rates of 16.9%–33.8% in dogs [86, 87] and 9.0% in cattle [101], the later found at a lower prevalence than we found in our study (14.9%) (Table 2). While infection rates documented in the three preceding studies were similar to those reported here, a higher figure was expected in those previous studies as canine and livestock samples were mainly from shelters and intensive commercial farms with high animal densities and reduced enclosures favoring the risk of infection and transmission. Discrepancies in prevalence rates among these surveys may be attributed to differences in the diagnostic performance of the screening method used, as light microscopy is usually a less sensitive technique than PCR for pathogen detection.

In neighboring Spain, *G. duodenalis* was reported in the Iberian wolf (16.7%), stone marten (12.5%) [30], red fox (9.6%) [34], and red deer (2.4%) [41], displaying lower prevalence values than those found in our study for the same evaluated hosts. The protozoan was described at similar prevalence rates (7.5%–8.9%) in roe deer [41, 43] (Table 1). As for domestic animal hosts, *G. duodenalis* has been reported across Europe, with prevalence ranging from 2.0% to 100% in dogs [75, 85] and 9.1% to 100% in cattle [55, 92] (see Table 2). Remarkably, *G. duodenalis* was apparently absent in domestic goats, the only host species analyzed in this study where this parasite was undetected. Caprine infections by *G. duodenalis* have been previously reported in a few European countries, namely Spain (19.8% [94]; 42.2% [102]) and Belgium (35.8%) [103]. Differences in environmental and anthropogenic pressures, the composition of wild species communities, and contact rates with livestock or companion animals in the sampled areas might explain the discrepant *G. duodenalis* results among studies.

### 4.2. Genetic Diversity of *Giardia duodenalis* Isolates

Nucleotide sequence analyses of *G. duodenalis* isolates at the *ssu*, *gdh*, and *bg* loci revealed the presence of zoonotic subassemblage AI in one roe deer (from MNP), canine-specific assemblages C and D in Iberian wolves, red foxes, and one dog (from CPW and MNP), and ungulate-specific assemblage E in wild boar, cattle, horse, and sheep (from different sampling locations). Assemblages B, C, and D were previously documented in dogs from Portugal [86, 87], while assemblages A, B, and E were reported in cattle [101]. In Spain, García-Presedo et al. [43] reported subassemblage AII in roe deer, which is considered the *G. duodenalis* genetic variant predominant in humans [14].

*Giardia duodenalis* assemblages C and D have been frequently reported in wolves (e.g., [25, 27]) and dogs (e.g., [61, 65]) populations across Europe (Table 1). In our study, assemblage D was found in wolves and a dog inhabiting the CPW area, displaying 100% identity with reference sequence AF199449 [205]. Interestingly, CPW sustains the most fragile and isolated subpopulation of Iberian wolves, which share their territory with feral dog packs and free-roaming shepherd dogs. As hybridization has already been confirmed between wolves and dogs in CPW [207], our finding indicates the possibility of a transmission route between the two hosts (Figure 2), which can occur either by direct contact or indirectly through environmental contamination of water or food resources with *Giardia* cysts. Additionally, a mixed infection of assemblages C + D was found in one red fox, suggesting that this host may also be involved in the sylvatic cycle of *G. duodenalis* (Figure 2). The report of assemblages C/D (in red fox) and E (in wild boar) is likely the first confirmation of these two species as hosts of these assemblages in Europe, representing another indicator of cross-species transmission. Additional evidence of overlapping sylvatic and domestic life cycles comes from the finding of assemblage E in a sheep from the same area (MNP) as the wild boar was reported, with both amplicons showing 100% identity with reference sequence AF199448 [205] (Table 4, Figure 2). Another interesting result was the identification of assemblage E in a horse from FBR since no other positive samples from cattle were typed in this geographical region. In this area, horses are subjected to a strict annual deworming scheme with ivermectin, while cattle have less rigorous protocols with occasional clorsulon–ivermectin administration. These antiparasitic drugs are essentially used to treat helminth (nematodes) and arthropod infections, while they proved ineffective against protozoan infections like giardiasis and cryptosporidiosis [208]. Therefore, the overall low prevalence of protozoan parasites found in FBR (8.3%) cannot be attributed to ongoing deworming protocols. Lower animal densities or environmental characteristics of the study area can be plausible explanations for reduced cyst contamination and transmission risk.

### 4.3. Prevalence of *Cryptosporidium* spp. in Wild and Domestic Species

Previous studies in Portugal have reported the presence of *Cryptosporidium* spp. in horses (21.4%) [181], sheep and cattle (17.6%–100%) [101, 123–125]. Nonetheless, information concerning this protozoan infection in wild reservoirs is restricted to a report in wild boar, where a prevalence of 1.4% (2/144) was detected [51]. This prevalence is lower than that reported here for the same host species (7.5%, 8/107) (Table 1). Across Europe, *Cryptosporidium* spp. has been described at highly variable prevalence (3.6%–35.7%) in wolves in eastern Slovakia [36] and Poland [35]. As for red fox, a similar prevalence to the one reported in this study was found in Poland (2.7%), the Czech Republic (1.7%), and Slovakia (2.1%) [36], while the highest reported prevalence was found in Ireland (20%) [38]. Even though we could not detect *Cryptosporidium* spp. in any of the analyzed stone martens, this protozoan was documented in this host species in Poland (29.4%) [39]. For wild ungulates, a similar prevalence to the one we reported for red deer was found in neighboring Spain (2.7%) [41]. Higher infection rates were described for wild boar in Austria (18.1%), Czech Republic (16.9%), Poland (8.5%) [49], and Spain (16.8%) [50] (Table 1). In domestic dogs, the highest prevalence was reported in Germany (10.0%) [89]. Furthermore, while *Cryptosporidium* spp. was not detected in any of the livestock samples analyzed, across Europe, literature reports are extensive, particularly for cattle, reporting highly variable prevalence rates (4.9%–100%) [119, 146] (Table 2). One of the reasons behind the lack of detection of this protozoan in our livestock analyzed samples may be related to the fact that we sampled adult individuals, and as previous studies have shown, *Cryptosporidium* infections are more frequent in younger animals, particularly neonatal calves [2, 94, 96, 209]. Furthermore, bovine *Cryptosporidium* infections are generally short-lived, with oocyst shedding lasting 1–2 weeks, decreasing the time frame where it would be possible to detect the parasite in the feces effectively [210].

### 4.4. Genetic Diversity of *Cryptosporidium* spp. Isolates

Six *Cryptosporidium* species were identified circulating in the wild, and domestic species investigated in the present survey (Table 4). Swine-adapted *C. scrofarum* (formerly known as pig genotype II) was the most prevalent species detected but was exclusively found in wild boars. *Cryptosporidium scrofarum* has been reported in wild boar across Europe [41, 44, 49], including Portugal [51]. Wild boars were also the reservoir where a higher prevalence of *Cryptosporidium* spp. (8.4%) was found, previously associated with their omnivorous diet and broader habitat selection requirements [209]. Canine-adapted *C. canis* was detected in the Iberian wolf, as previously described in wolves from Slovakia [36] and in red fox, as reported in Spain [30, 34] and Poland [39]. As for the identification of *C. ryanae* and *C. occultus* in red deer, these results agree with a report from Spain [41]. Interestingly, both studies found *C. occultus* in red deer, a species typically associated with rodents. This suggests the existence of a potential transmission route between these two hosts. Furthermore, the detection of *C. ubiquitum* in red deer and wild boar inhabiting the same area (MNP) suggests that both species are involved in the transmission of the parasite (Figure 3).

The first description of *C. felis* in the Iberian lynx has provided important insights into the potential pathogens that could threaten the successful reintroduction of this endangered species. However, the potential sources of infection remain unclear. Since domestic cats are the acknowledged reservoir of *C. felis* [14], one possibility is that Iberian lynxes acquire the parasite through spillover events between domestic and sylvatic transmission cycles [211]. Another possibility is that *C. felis* naturally circulates in the wild Iberian lynx population.

Apart from ungulate-adapted *C. ryanae*, all *Cryptosporidium* spp. identified in this study and *G. duodenalis* subassemblage AI have zoonotic potential [14]. This fact suggests that the wild and domestic host species can act as potential reservoirs of human cryptosporidiosis and giardiasis, in addition to a source of environmental contamination with infective (oo)cysts.

Even though none of our analyzed wild and domestic ungulate samples yielded positive results for *B. coli*, genotypes A and B were previously described in Portugal in red deer and wild boar [51, 52], as well as in wild boar populations from Spain [41, 44] and Poland [53] (Table 1). Contrary to the other free-ranging livestock species, pigs (*B. coli* primary host) are raised inside enclosures in the sampled areas, restricting their contact with wild boars, which can explain why we did not find this parasite in any of the analyzed samples.

The experiences made during our study may guide future research. Larger sample sizes from some species were due to stored-up collection or ongoing monitoring projects in the sampled areas (e.g., Iberian wolf, red fox, and wild boar), while smaller sample sizes can be attributed to the fact that we are working with endangered species (Iberian lynx) or species with limited distribution in the sampled areas (e.g., stone marten and domestic goat). Without targeted monitoring programs for the latter species, this will likely remain a limitation. The opportunistic sampling limited our ability to capture seasonal variability of pathogen occurrence or to compare potential effects of age, sex, and different sources of sampling. This is particularly important as cryptosporidiosis consistently occurs in younger animals, especially domestic animals, which we failed to sample. Nonetheless, it is unlikely this can be done for endangered species, and more in-depth studies could focus on the more common species. Last, our genotyping PCRs' relatively low amplification was associated with the limited sensibility of the single-copy genes (*gdh*, *bg*, and *tpi* in *G. duodenalis* or *gp60* in *Cryptosporidium* spp.) targeted in our PCR, associated with the small amount of parasite DNA in the analyzed samples, which hampered the attempts to assess the zoonotic potential and the public health significance in the analyzed samples.

## 5. Conclusions

Our findings contributed to bridging the knowledge gap regarding the epidemiology of protist species of public and veterinary health relevance in wild and domestic host species from Portugal. The identification of zoonotic *Cryptosporidium* spp. and *G. duodenalis* subassemblage AI highlights the role played by wild and domestic species in the maintenance of the sylvatic and domestic cycle of such organisms. These findings are a step forward to unraveling the epidemiological scenario in the Portuguese context while comparing it to other European studies (Tables 1 and 2), which is critical knowledge for understanding the possible infection risks that human populations may be facing in the sampled areas. Future studies should not only aim to cover additional ecological niches but also target host-dependent risk factors such as host age, as cryptosporidiosis consistently occurs in younger animals, especially in domestic species. Although not fully understood, the identification of *G. duodenalis* and *Cryptosporidium* infections in endangered species (e.g., Iberian wolf and Iberian lynx) may have important conservation implications, which should be addressed in future research. Therefore, it is essential to implement tailor-made conservation measures to attain the specific needs of these species, including the regular monitoring programs of these enteric protozoan parasites and other emerging infectious pathogens, with the ultimate goal of preserving biological diversity.

## Figures and Tables

**Table 1:** Prevalence and molecular diversity of *Giardia duodenalis*, *Cryptosporidium* spp., and *Balantioides coli* reported in the wild carnivores and ungulates in Europe.

Pathogen	Host scientific name	Country	Occurrence rate (no. pos./total no.)	Detection method	Locus	Species/genotype (*n*)	Subgenotype (*n*)	Reference
Wild carnivores								
*Giardia duodenalis*	*Canis lupus*	Croatia	10 (13/127)	CM, PCR	*ssu*-rRNA, ITS1-ITS2, *tpi*, *tpi*-D^2^	A (6), C (2), D (1), A + B + D (1), A + C + D (1), C + D (1)	A1^3^ (6)	Beck et al. [25]
		Poland	29 (2/7)	CM, PCR	*bg*	D (2)	–	Stojecki et al. [26]
		Romania	100 (3/3)	PCR^1^	*gdh*	D (3)	–	Adriana et al. [27]
		Italy	5 (1/20)	PCR	*ssu*-rRNA	C (1)	–	Di Francesco et al. [28]
		Italy	100 (1/1)	PCR^1^	*ssu*-rRNA	D (1)	–	Guadano Procesi et al. [29]
	*C. lupus signatus*	Spain	17 (1/6)	PCR	*gdh*	NA	–	Mateo et al. [30]
		Portugal	26 (31/121)	PCR	*ssu*-rRNA, *gdh*, *bg*, *tpi*	D (4), C + D (2)	–	This study
	*Lynx pardinus*	Portugal	27 (8/30)	PCR	*ssu*-rRNA, *gdh*, *bg*, *tpi*	NA	NA	This study
	*Vulpes vulpes*	Norway	5 (13/269)	CM, PCR	*gdh*, *bg*	A (5), B (2)	A1^3^ (2), A2^3^ (1), B3^3^ (1)	Hamnes et al. [31]
		Croatia	5 (3/66)	CM, PCR	*ssu*-rRNA, ITS1-ITS2, *tpi*, *tpi*-D^2^	A (1)	NA	Beck et al. [25]
		Romania	5 (10/217)	PCR	*bg*	A (2), B (1)	AII (2)	Onac et al. [32]
		Sweden	44 (46/104)	CM, PCR	*gdh*, *bg*, *tpi*	B (4)	NA	Debenham et al. [33]
		Spain	8 (7/87)	PCR	*gdh*	NA	NA	Mateo et al. [30]
		Spain	10 (19/197)	PCR	*ssu*-rRNA, *gdh*, *bg*	NA	NA	Barrera et al. [34]
		Portugal	19 (22/118)	PCR	*ssu*-rRNA, *gdh*, *bg*, *tpi*	C + D (1)	–	This study
	*Martes foina*	Spain	13 (1/8)	PCR	*gdh*	NA	NA	Mateo et al. [30]
		Portugal	16 (3/19)	PCR	*ssu*-rRNA, *gdh*, *bg*, *tpi*	NA	NA	This study
*Cryptosporidium* spp.	*Canis lupus*	Poland	36 (5/14)	CM, PCR	*cowp*	*C. parvum* genotype 2 (5)	NA	Paziewska et al. [35]
		Czech Republic	6 (1/17)	CM, PCR	*ssu*-rRNA, actin, *gp60*	*C. ubiquitum* (1)	XIId (1)	Kváč et al. [36]
		Slovakia	4 (3/83)	CM, PCR	*ssu*-rRNA, actin, *gp60*	*C. canis* (2), *C. ubiquitum* (1)	XIId (1)	Kváč et al. [36]
		Slovakia	ND (4/ND)	PCR	ND	*C. scrofarum* (4)	–	Valenčáková et al. [37]
	*C. lupus signatus*	Portugal	3 (3/121)	PCR	*ssu*-rRNA, *gp60*	*C. canis* (3)	NA	This study
	*Lynx pardinus*	Portugal	3 (1/30)	PCR	*ssu*-rRNA	*C. felis* (1)	–	This study
	*Vulpes vulpes*	Ireland	20 (2/10)	CM, PCR	*ssu*-rRNA, *gp60*	*C. parvum* (2)	Ic (1), II (1)	Nagano et al. [38]
		Spain	8 (7/87)	PCR	*ssu*-rRNA	*C. parvum* (3), *C. canis* (2), *C. felis* (1), *C. ubiquitum* (1)	NA	Mateo et al. [30]
		Spain	6 (12/197)	PCR	*ssu*-rRNA, *gp60*	*C. hominis* (4), *C. canis* (3), *C. parvum* (2), *C. ubiquitum* (1), *C. suis* (1), *Cryptosporidium* spp. (1)	NA	Barrera et al. [34]
		Czech Republic	2 (1/58)	CM, PCR	*ssu*-rRNA, actin, *gp60*	*C. tyzzeri* (1)	IXa (1)	Kváč et al. [36]
		Poland	3 (2/74)	CM, PCR	*ssu*-rRNA, actin, *gp60*	*C. andersoni* (2)	–	Kváč et al. [36]
		Poland	12 (6/50)	PCR	*ssu*-rRNA, actin	*C. canis* (3), *C. alticolis* (2), *C*. vole genotype II (1)	–	Perec-Matysiak et al. [39]
		Slovakia	2 (1/47)	CM, PCR	*ssu*-rRNA, actin, *gp60*	*C. galli* (1)	–	Kváč et al. [36]
		Portugal	3 (4/118)	PCR	*ssu*-rRNA, *gp60*	*C. canis* (4)	NA	This study
	*Martes foina*	Poland	29 (15/51)	PCR	*ssu*-rRNA, actin	*C. ditrichi* (15)	–	Perec-Matysiak et al. [39]
Wild ungulates								
*Giardia duodenalis*	*Cervus elaphus*	Croatia	1 (4/374)	CM, PCR	*ssu*-rRNA, ITS1-5.8S-ITS2, *tpi*, *tpi*-D^2^	A (4), D (1)	A1^3^ (2), A3^3^ (1)	Beck et al. [25]
		Poland	2 (1/61)	CM, PCR	*gdh*, *bg*, *tpi*	A (1)	AIII (1)	Solarczyk et al. [40]
		Poland	18 (5/28)	CM, PCR	*bg*	B (4)	NA	Stojecki et al. [26]
		Spain	2 (8/329)	PCR	*ssu*-rRNA, *gdh*, *bg*, *tpi*	NA	NA	Dashti et al. [41]
		Portugal	4 (4/96)	PCR	*ssu*-rRNA, *gdh*, *bg*, *tpi*	NA	NA	This study
	*Capreolus capreolus*	The Netherlands	100 (1/1)	PCR^1^	*ssu*-rRNA, *gdh*	A (1)	NA	van der Giessen et al. [42]
		Croatia	24 (5/21)	CM, PCR	*ssu*-rRNA, ITS1-5.8S-ITS2, *tpi*, *tpi*-D^2^	A (2), C (1), D (2)	A1^3^ (1), A3^3^ (1)	Beck et al. [25]
		Poland	4 (2/50)	CM, PCR	*gdh*, *bg*, *tpi*	A (2)	AI (2)	Solarczyk et al. [40]
		Poland	23 (11/48)	CM, PCR	*bg*	B (8)	NA	Stojecki et al. [26]
		Spain	9 (19/212)	CM, PCR	*bg*	A (7)	AII (7)	García-Presedo et al. [43]
		Spain	8 (7/93)	PCR	*ssu*-rRNA, *gdh*, *bg*, *tpi*	NA	NA	Dashti et al. [41]
		Romania	100 (4/4)	PCR^1^	*gdh*	E (4)	–	Adriana et al. [27]
		Portugal	10 (4/39)	PCR	*ssu*-rRNA, *gdh*, *bg*, *tpi*	A (1)	AI (1)	This study
	*Sus scrofa*	Croatia	1 (2/144)	CM, PCR	*ssu*-rRNA, ITS1-5.8S-ITS2, *tpi*, *tpi*-D^2^	A (1)	A3^3^ (1)	Beck et al. [25]
		Poland	41 (11/27)	CM, PCR	*bg*	B (6)	NA	Stojecki et al. [26]
		Spain	23 (32/142)	PCR	*gdh*, *bg*, *tpi*	NA	NA	Rivero-Juarez et al. [44]
		Spain	6 (20/359)	PCR	*ssu*-rRNA, *gdh*, *bg*, *tpi*	A (1)	NA	Dashti et al. [41]
		Spain	1 (6/498)	PCR	*ssu*-rRNA, *gdh*, *bg*	E (1)	NA	Martí-Marco et al. [45]
		Italy	100 (4/4)	PCR^1^	*ssu*-rRNA, *gdh*, *bg*, *tpi*	A (4), D (1)	AI (2), AII (2)	Guadano Procesi et al. [29]
		Portugal	15 (16/107)	PCR	*ssu*-rRNA, *gdh*, *bg*, *tpi*	E (1)	–	This study
*Cryptosporidium* spp.	*Cervus elaphus*	Czech Republic	100 (1/1)	PCR^1^	*ssu*-rRNA, *cowp*	*C. parvum* (1)	–	Hajdušek et al. [46]
								
		Poland	4 (6/136)	CM, PCR	*ssu*-rRNA, *gp60*	*C. ubiquitum* (5), *C. muris* (1)	XIId (5)	Kotková et al. [47]
		Spain	3 (9/329)	PCR	*ssu*-rRNA, *gp60*	*C. ryanae* (7), *C. parvum* (1), *C. suis* (1)	NA	Dashti et al. [41]
		Portugal	3 (3/96)	PCR	*ssu*-rRNA, *gp60*	*C. ubiquitum* (1), *C. ryanae* (1), *C. suis* (1)	NA	This study
	*Capreolus capreolus*	Spain	4 (9/212)	CM, PCR	*ssu*-rRNA	*C. bovis* (3), *C. ryanae* (3)	–	García-Presedo et al. [43]
		Spain	8 (7/93)	PCR	*ssu*-rRNA, *gp60*	*C. ryanae* (5), *C. ubiquitum* (1), *C. canis* (1)	NA	Dashti et al. [41]
		Italy	4 (4/119)	CM, PCR	*cowp*, *gp60*	*C. ubiquitum* (4)	NA	Trogu et al. [48]
	*Sus scrofa*	Czech Republic	17 (32/193)	CM, PCR	*ssu*-rRNA	*C. suis* (18), *C. scrofarum*^3^ (7), *C. suis/C. scrofarum*^4^ (12)	–	Němejc et al. [49]
		Czech Republic	17 (39/231)	CM, PCR	*ssu*-rRNA, *gp60*	*C. scrofarum* (14), *C. suis* (13), *C. suis/C. scrofarum* (12)	–	Němejc et al. [49]
		Austria	18 (8/44)	CM, PCR	*ssu*-rRNA, *gp60*	*C. suis* (2), *C. scrofarum* (1), *C. suis/C. scrofarum* (2)	–	Němejc et al. [49]
		Poland	9 (11/129)	CM, PCR	*ssu*-rRNA, *gp60*	*C. scrofarum* (8), *C. suis* (1), *C. suis/C. scrofarum* (2)	–	Němejc et al. [49]
		Slovak Republic	5 (3/56)	CM, PCR	*ssu*-rRNA, *gp60*	*C. suis* (2), *C. scrofarum* (1)	–	Němejc et al. [49]
		Slovakia	ND (3/ND)	PCR	ND	*C. scrofarum* (1), C. *suis* (2)	–	Valenčáková et al. [37]
		Spain	17 (35/209)	CM, PCR	*ssu*-rRNA, *gp60*	*C. scrofarum* (19), *C. suis* (5), *C. parvum* (3)	IIa (3)	García-Presedo et al. [50]
		Spain	6 (9/142)	PCR	*ssu*-rRNA	*C. scrofarum* (8), *C. suis* (1)	–	Rivero-Juarez et al. [44]
		Spain	7 (25/359)	PCR	*ssu*-rRNA, *gp60*	*C. scrofarum* (22), *C. ryanae* (1), *C. parvum* (1), *Cryptosporidium* spp. (1)	NA	Dashti et al. [41]
		Spain	22 (108/498)	PCR	*ssu*-rRNA	*C. scrofarum* (94), *C. suis* (14)	NA	Martí-Marco et al. [45]
		Portugal	1 (2/144)	PCR	*ssu*-rRNA	*C. scrofarum* (2)	–	Santos-Silva et al. [51]
		Portugal	8 (9/107)		*ssu*-rRNA, *gp60*	*C. ubiquitum* (1), *C. scrofarum* (8)	NA	This study
*Balantioides coli*	*Cervus elaphus*	Portugal	4 (4/95)	PCR	ITS	A (2), B (2)	–	Mega et al. [52]
	*Sus scrofa*	Czech Republic	100 (1/1)	PCR^1^	ITS	A (1)	–	Pomajbíková et al. [53]
		Spain	12 (16/142)	PCR	ITS	NA	–	Rivero-Juarez et al. [44]
		Spain	3 (9/359)	PCR	ITS	NA	–	Dashti et al. [41]
		Portugal	15 (21/144)	PCR	ITS	A (16), B (5)	–	Santos-Silva et al. [51]

*Note. bg*, *β*-giardin; CM, conventional microscopy; *cowp*, *Cryptosporidium* oocyst wall protein; *gdh*, glutamate dehydrogenase; *gp60*, 60-kDa glycoprotein; ITS, internal transcribed spacer; NA, no amplification; ND, no data available; PCR, polymerase chain reaction; *ssu*-rRNA, small subunit ribosomal RNA; *tpi*, triose phosphate isomerase. ^1^PCR conducted only in samples with a previous positive result by another detection method. ^2^Primer sequences specifically designed to amplify assemblage D. ^3^Previous nomenclature adopted by the authors. ^4^Formerly known as *Cryptosporidium* pig genotype II.

**Table 2:** Prevalence and molecular diversity of *Giardia duodenalis* and *Cryptosporidium* spp. reported in domestic dogs and livestock species in Europe.

Pathogen	Host scientific name	Country	Occurrence rate (no. pos./total no.)	Detection method	Locus	Species/genotype (*n*)	Subgenotype (*n*)	Reference
Domestic carnivore								
*Giardia duodenalis*	*Canis lupus familiaris*	Italy	15 (17/113)	CM, PCR	*ssu*-rRNA	A (2), C (11), D (1), A + C (2), C + D (1)	–	Berrilli et al. [54]
		Italy	100 (21/21)	PCR	*ssu*-rRNA, *gdh*, *bg*	A (6), C (1), D (13), A + D (1)	A1^3^ (5), A8^3^ (1), D1^3^ (7), D2^3^ (4), D1 + D2^3^ (2)	Lalle et al. [55]
		Italy	19 (20/101)	CM, PCR	*tpi*	A (3), C (17)	A1^3^ (3), C1^3^ (13), C2^3^ (4)	Papini et al. [56]
		Italy	21 (26/127)	CM, PCR	*ssu*-rRNA	A (8), C (14), D (4)	–	Scaramozzino et al. [57]
		Italy	64 (9/14)	CM, PCR	*bg*	A (9)	A1^3^ (9)	Marangi et al. [58]
		Italy	58 (165/285)	CM, PCR	*ssu*-rRNA, *bg*	B (1), C (49), D (29)	B1^3^ (1)	Simonato et al. [59]
		Italy	29 (204/705)	CM, PCR	*ssu*-rRNA, *bg*	B (1), C (9), D (11)	NA	Simonato et al. [60]
		Italy	21 (56/262)	CM, PCR	*ssu*-rRNA	C (6), D (19)	–	Liberato et al. [61]
		Italy	41 (69/168)	CM, PCR	*ssu*-rRNA, *bg*, *tpi*	A (16), B (6), C (2), A + B (1)	AII (5)	Agresti et al [62]
		The Netherlands	100 (2/2)	PCR^1^	*ssu*-rRNA, *gdh*	D (2)	–	van der Giessen et al. [42]
		The Netherlands	15 (14/92)	CM, PCR	*ssu*-rRNA	A (1), C (7), D (3), C + D (1)	–	Overgaauw et al. [63]
		The Netherlands	86 (493/573)	CM, PCR	*ssu*-rRNA	NA	–	Uiterwijk et al. [64]
		Hungary	59 (110/187)	CM, PCR	*ssu*-rRNA	C (5), D (9), C + D (1)	–	Szénási et al. [65]
		Germany	100 (92/92)	PCR^1^	*ssu*-rRNA	C (33), D (50), C + D (8), A + D (1)	–	Barutzki et al. [66]
		Germany	92 (55/60)	CM, PCR	*ssu*-rRNA, *gdh*	A (33), C (5), D (2), A + C (15)	AI (14)	Leonhard et al. [67]
		Germany	6 (5/81)	PCR	*gdh*	A (5)	NA	Sotiriadou et al. [68]
		Germany	95 (123/130)	CM, PCR	*ssu*-rRNA, *gdh*, *bg*	A (24), B (8), C (52), D (69), F (6)	A1^3^ (3), A2^3^ (1), A5^3^ (4), A1/A5^3^ (2), BIII (3)	Pallant et al. [69]
		Germany	42 (13/31)	CM, PCR	*ssu*-rRNA, *gdh*, *bg*, *tpi*	A (2), C (1), D (2), A + B (1)	AI (2)	Rehbein et al. [70]
		Germany	31 (115/376)	CM, PCR	*ssu*-rRNA, *gdh*, *tpi*, *tpi*-D^2^	A (2), C (42), D (52), E (2), F (2)	NA	Sommer et al. [71]
		Germany	29 (112/386)	CM, PCR	*ssu*-rRNA, *gdh*, *bg*	A (9), C (43), D (40), C + D (5), D + A (4), C + A (3)	AI (9)	Murnik et al. [72]
		Belgium	23 (270/1159)	CM, PCR	*bg*	A (40), B (4), C (26), D (49)	A2^3^ (2), A3^3^ (36), D1^3^ (16), D2^3^ (25)	Claerebout et al. [73]
		United Kingdom	10 (87/878)	CM, PCR	*ssu*-rRNA, *bg*	A (1), C (10), D (29), C + D (1)	A3^3^ (1), D1^3^ (2), D2^3^ (8)	Upjohn et al. [74]
		Poland	2 (3/148)	CM, PCR	*bg*	C (1), D (1)	–	Solarczyk and Majewska [75]
		Poland	29 (31/108)	CM, PCR	*tpi*	C (2)	–	Bajer et al. [76]
		Poland	19 (7/36)	CM, PCR	*bg*	A (1), E (1)	NA	Stojecki et al. [77]
		Poland	6 (13/217)	PCR	*bg*	C (10), D (3)	–	Piekara-Stępińska et al. [78]
		Spain	16 (96/604)	CM, PCR	*gdh*, *bg*	A (3), B (22), C (1), D (7), A + B (3), A + C (3), A + E (1), B + C (3), B + D (11), B + E (4), C + D (1), C + E (1), B + C + D (3)	AI (10), BIII (21), BIV (25)	Dado et al. [79]
		Spain	37 (64/169)	CM, PCR	*gdh*, *bg*, *tpi*	C (1), D (4), C + D (1)	–	Ortuño et al. [80]
		Spain	29 (16/55)	CM, PCR	*ssu*-rRNA, *gdh*, *bg*	C (3)	–	De Lucio et al. [81]
		Spain	37 (127/348)	CM, PCR	*ssu*-rRNA, *gdh*, *bg*	A (5), B (8), C (2), D (13), A + B (4), A + D (1), A + B + D (2)	AII (11), AIII (2), BIII (7), BIV (8)	Adell-Aledón et al. [82]
		Croatia	62 (16/26)	CM, PCR	*ssu*-rRNA, ITS1-5.8S-ITS2, *gdh*, *bg*, *tpi*	C (6), D (10)	–	Sommer et al. [83]
		Macedonia	33 (45/136)	CM, PCR	*ssu*-rRNA, ITS1-5.8S-ITS2, *gdh*, *bg*, *tpi*	C (7), D (7)	–	Sommer et al. [83]
		Romania	36 (66/183)	CM, PCR	*ssu*-rRNA, ITS1-5.8S-ITS2, *gdh*, *bg*, *tpi*	C (8), D (8)	–	Sommer et al. [83]
		Romania	100 (39/39)	PCR^1^	*gdh*	C (8), D (29), C + D (1), E (1)	–	Adriana et al. [27]
		Serbia	66 (88/134)	CM, PCR	*ssu*-rRNA, ITS1-5.8S-ITS2, *gdh*, *bg*, *tpi*	C (8), D (6)	–	Sommer et al. [83]
		Greece	25 (220/879)	CM, PCR	*ssu*-rRNA, *gdh*, *bg*, *tpi*	A (2), C (45), D (27), C + D (15), A + C (2), A + D (1), B + C (1) A + C + D (2)	AI (1), AII (1), BIV (1)	Kostopoulou et al. [84]
		Czech Republic	100 (54/54)	PCR^1^	*gdh*, *bg*, *tpi*	C (21), D (32), C + D (1)	–	Lecová et al. [85]
		Portugal	17 (25/148)	CM, PCR	*ssu*-rRNA	B (1), C (15), D (12)	–	Ferreira et al. [86]
		Portugal	34 (27/80)	CM, PCR	*gdh*, *bg*, *tpi*	C (3), D (11), C + D (4)	–	Pereira et al. [87]
		Portugal	24 (11/46)	PCR	*ssu*-rRNA, *gdh*, *bg*, *tpi*	D (1)	–	This study
*Cryptosporidium* spp.	*Canis lupus familiaris*	Czech Republic	100 (2/2)	PCR^1^	*ssu*-rRNA, *cowp*	*C. parvum* (1), *C. meleagridis* (1)	–	Hajdušek et al. [46]
		Italy	3 (8/240)	PCR	*cowp*	*C. parvum* (7), *C. canis* (1)	–	Giangaspero et al. [88]
		Italy	1 (3/285)	PCR	*cowp*	*C. parvum* (3)	–	Simonato et al. [59]
		Italy	2 (12/705)	PCR	*ssu*-rRNA, *cowp*	*C. parvum* (11), *C. canis* (1)	–	Simonato et al. [60]
		Germany	1 (1/81)	PCR	*ssu*-rRNA	*C. parvum* (1)	–	Sotiriadou et al. [68]
		Germany	10 (35/349)	PCR	*ssu*-rRNA, *gp60*	*C. canis* (33), *C. parvum* (2)	IIa (2), XXd (5), XXe (6), XXb (2)	Murnik et al. [89]
		France	3 (3/116)	CM, PCR	*ssu*-rRNA	*C. canis* (3)	–	Osman et al. [90]
		Greece	6 (52/879)	CM, PCR	*ssu*-rRNA, *hsp70*	*C. canis* (2), *C. scrofarum* (1)	–	Kostopoulou et al. [84]
		Spain	6 (3/55)	CM, PCR	*ssu*-rRNA	*C. canis* (2), *Cryptosporidium* spp. (1)	–	De Lucio et al. [81]
		Norway	1 (1/170)	CM, PCR	*ssu*-rRNA	*C. canis* (1)	–	Myšková et al. [91]
Domestic ungulates								
*Giardia duodenalis*	*Bos taurus*	The Netherlands	9 (57/628)	CM, PCR	*gdh*	A (5), B (4)	NA	Huetink et al. [92]
		Italy	30 (3/10)	CM, PCR	*ssu*-rRNA	E (3)	–	Berrilli et al. [54]
		Italy	100 (24/24)	CM, PCR	*ssu*-rRNA, *gdh*, *bg*	A (12), B (5), E (3), A + B (2), A + E (2)	A1^3^ (8), A2^3^ (1), A3^3^ (2), A4^3^ (1), B3^3^ (1), B5^3^ (2), B6^3^ (2), E2^3^ (1), E3^3^ (1), E1 + E2^3^ (1)	Lalle et al. [55]
		Italy	32 (162/503)	CM, PCR	*bg*, *tpi*	A (4), E (7), A + E (3)	NA	Geurden et al. [93]
		Spain	27 (101/379)	CM, PCR	*gdh*, *bg*	E (4)	–	Castro-Hermida et al. [94]
		Spain	19 (68/362)	PCR	*ssu*-rRNA, *gdh*, *bg*	E (15), F (4)	–	Cardona et al. [95]
		Spain	30 (192/649)	CM, PCR	*bg*	A (18), E (32)	AI (39)	Castro-Hermida et al. [96]
		Denmark	35 (401/1150)	CM, PCR	*ssu*-rRNA, *gdh*	A (8), E (137)	NA	Langkjær et al. [97]
		Belgium	31 (259/832)	CM, PCR	*bg*, *tpi*	A (43), E (77)	A2^3^ (ND), A3^3^ (ND), E2^3^ (ND), E3^3^ (ND)	Geurden et al. [98]
		France	40 (190/477)	CM, PCR	*bg*, *tpi*	A (8), E (4), A + E (8)	NA	Geurden et al. [93]
		Germany	51 (274/536)	CM, PCR	*bg*, *tpi*	A (8), E (9), A + E (7)	NA	Geurden et al. [93]
		Germany	73 (110/152)	PCR	*ssu*-rRNA, *gdh*, *bg*	A (8), E (101), A + E (1)	A1^3^ (1), E2^3^ (1), E3^3^ (8)	Gillhuber et al. [99]
		United Kingdom	55 (305/556)	CM, PCR	*bg*, *tpi*	A (3), E (2), A + E (4)	NA	Geurden et al. [93]
		Poland	17 (16/86)	CM, PCR	*bg*	A (1), E (3)	NA	Stojecki et al. [77]
		Austria	27 (48/177)	CM, PCR	*ssu*-rRNA, *bg*, *tpi*	A (1), E (30)	NA	Lichtmannsperger et al. [100]
		Portugal	9 (42/467)	CM, PCR	*gdh*, *bg*	A (2), B (1), E (11)	A2^3^ (2)	Mendonça et al. [101]
		Portugal	15 (13/87)	PCR	*ssu*-rRNA, *gdh*, *bg*, *tpi*	E (1)	–	This study
	*Capra hircus*	The Netherlands	100 (1/1)	PCR^1^	*ssu*-rRNA, *gdh*	E (1)	–	van der Giessen et al. [42]
		Spain	20 (23/116)	CM, PCR	*gdh*, *bg*	E (1)	–	Castro-Hermida et al. [94]
		Spain	42 (133/315)	CM, PCR	*bg*, *tpi*	E (31)	–	Ruiz et al. [102]
		Belgium	36 (53/148)	CM, PCR	*bg*, *tpi*	A (6), E (17), E + A (5)	A2^3^ (6), E2^3^ (6), E3^3^ (16)	Geurden et al. [103]
		Greece	40 (103/255)	CM, PCR	*bg*, *tpi*	A (1), E (26), A + E (3)	NA	Tzanidakis et al. [104]
	*Equus caballus*	Italy	13 (20/150)	CM, PCR	*ssu*-rRNA	E (20)	–	Veronesi et al. [105]
		Italy	9 (37/431)	PCR	*ssu*-rRNA, *bg*	A (16), B (11), E (10)	A1^3^ (3), B1-2^3^ (8), B1-6^3^ (2), E3^3^ (1)	Traversa et al. [106]
		Belgium	14 (19/134)	CM, PCR	*bg*, *tpi*	A (8), B (3)	AI (6), AII (2), BIV (3)	Kostopoulou et al. [107]
		Germany	10 (3/30)	CM, PCR	*bg*, *tpi*	NA	NA	Kostopoulou et al. [107]
		Greece	12 (22/190)	CM, PCR	*bg*, *tpi*	A (4), B (9), E (2)	AI (3), AII (1), BIV (9)	Kostopoulou et al. [107]
		The Netherlands	11 (5/44)	CM, PCR	*bg*, *tpi*	A (1), B (1)	AII (1)	Kostopoulou et al. [107]
		Poland	10 (1/10)	CM, PCR	*bg*	E (1)	–	Stojecki et al. [77]
		Portugal	8 (2/26)	PCR	*ssu*-rRNA, *gdh*, *bg*, *tpi*	E (1)	–	This study
	*Ovis aries*	Italy	2 (5/325)	CM, PCR	*gdh*, *bg*	A (5)	AI (5)	Giangaspero et al. [108]
		Italy	100 (3/3)	PCR^1^	*tpi*	B (3)	B1^2^	Aloiso et al. [10]
		The Netherlands	100 (2/2)	PCR^1^	*ssu*-rRNA, *gdh*	E (2)	–	van der Giessen et al. [42]
		Spain	33 (118/575)	CM, PCR	*bg*	E (NA)	–	Castro-Hermida et al. [109]
		Spain	19 (86/446)	CM, PCR	*gdh*, *bg*	E (11), B (1)	NA	Castro-Hermida et al. [94]
		Spain	42 (162/386)	CM, PCR	*bg*	A (1), E (74)	A1^3^ (1), E1^3^ (2), E2^3^ (2)	Gómez-Muñoz et al. [110]
		Spain	30 (112/377)	CM, PCR	*bg*	B (11), E (20)	–	Castro-Hermida et al. [96]
		Spain	89 (107/120)	CM, PCR	*ssu*-rRNA, *gdh*, *bg*, *tpi*	A (59), E (21), A + E (27)	AI (7), AII (1)	Gómez-Muñoz et al. [111]
		Belgium	26 (36/137)	CM, PCR	*bg*, *tpi*	A (2), E (4), E + A (2)	A2^3^ (2), E2^3^ (1), E3^3^ (5)	Geurden et al. [103]
		Norway	22 (244/1095)	CM, PCR	*gdh*, *bg*	B (1), E (47)	NA	Robertson et al. [112]
		Greece	37 (160/429)	CM, PCR	*bg*, *tpi*	A (1), E (35), A + E (3)	NA	Tzanidakis et al. [104]
		Poland	22 (18/81)	CM, PCR	*bg*	A (6), E (10)	NA	Stojecki et al. [77]
		Portugal	17 (8/46)	PCR	*ssu*-rRNA, *gdh*, *bg*, *tpi*	E (2)	–	This study
*Cryptosporidium* spp.	*Bos taurus*	Poland	43 (146/342)	CM, PCR	*ssu*-rRNA	*C. muris* (1), *C. felis* (1)	–	Bornay-Llinares et al. [113]
		Poland	17 (119/700)	PCR	*ssu*-rRNA, *cowp*, LIB13	*C. bovis* (52), *C. parvum* (36), *C. andersoni* (17), *C. ryanae* (8)	–	Rzeżutka and Kaupke [114]
		Poland	10 (76/779)	PCR	*ssu*-rRNA, *cowp*, LIB13, *gp60*	*C. parvum* (76)	IIa (64), IId (7), IIl (5)	Kaupke and Rzeżutka [115]
		Poland	45 (725/1601)	PCR	*ssu*-rRNA, *gp60*	*C. parvum* (98), *C. parvum + C. bovis* (2), *Cryptosporidium* spp. (625)	IIa (80), IId (2)	Kaupke and Rzeżutka [116]
		Czech Republic	100 (4/4)	PCR^1^	*ssu*-rRNA, ITS1, *hsp70*	*C. muris* (4)	–	Morgan et al. [117]
		Czech Republic	100 (11/11)	PCR^1^	*ssu*-rRNA, *hsp70*	*C. andersoni* (9), *C. parvum*, (2)	–	Ryan et al. [118]
		Czech Republic	100 (2/2)	PCR^1^	*ssu*-rRNA, *cowp*	*C. parvum* (1), *C. andersoni* (1)	–	Hajdušek et al. [46]
		Czech Republic	5 (49/995)	CM, PCR	*ssu*-rRNA, *gp60*	*C. andersoni* (41), *C. bovis* (2), *C. parvum* (1)	IIa (1)	Ondráčková et al. [119]
		Czech Republic	21 (159/750)	CM, PCR	*ssu*-rRNA, *gp60*	*C. parvum* (137), *C. andersoni* (21), *C. bovis* (3)	IIa (131)	Kváč et al. [120]
		The Netherlands	9 (54/628)	CM, PCR	*cowp*	*C. parvum* (27)	–	Huetink et al. [92]
		The Netherlands	100 (160/160)	PCR^1^	*ssu*-rRNA, *hsp70*, *cowp*, *gp60*	*C. parvum* (160)	IIa (129), IIj (1)	Wielinga et al. [121]
		The Netherlands	17 (69/399)	PCR	*ssu*-rRNA, *gp60*	*C. parvum* (59), *C. bovis* (6), *C. ryanae* (4)	IIa (47)	Pinto et al. [122]
		Portugal	23 (129/553)	CM, PCR	*cowp*, TRAP-C1	*C. parvum* (14)	–	Fonseca et al. [123]
		Portugal	100 (35/35)	PCR^1^	*ssu*-rRNA, *gp60*	*C. parvum* (35)	IIa (29), IId (3)	Alves et al. [124]
		Portugal	98 (40/41)	PCR^1^	*gp60*	*C. parvum* (40)	IIa (39), IId (1)	Alves et al. [125]
		Portugal	18 (82/467)	CM, PCR	*ssu*-rRNA, *hsp70*	*C parvum* (78), *C. meleagridis* (1), *C. andersoni* (1)	–	Mendonça et al. [101]
		Denmark	31 (90/292)	CM, PCR	*ssu*-rRNA	*C. andersoni* (59), *C. parvum* (21), *C. andersoni* + *C. parvum* (10)	–	Enemark et al. [126]
		Denmark	56 (108/193)	CM, PCR	*cowp*, *ssu*-rRNA	*C. parvum* genotype II (108)	–	Enemark et al. [127]
		Denmark	29 (336/1150)	CM, PCR	*ssu*-rRNA, *hsp70*	*C. parvum* (79), *C. bovis* (57), *C*. deer-like genotype^4^ (11), *C. suis* (2)	–	Langkjær et al. [97]
		Ireland	7 (21/288)	CM, PCR	*ssu*-rRNA	*C. andersoni* (11), *C. parvum* genotype II (10)	–	Moriarty et al. [128]
		Ireland	37 (291/779)	CM, PCR	*ssu*-rRNA, *gp60*	*C. parvum* (215), *C. bovis* (5), *C*. deer-like genotype^4^ (3)	IIa (215)	Thompson et al. [129]
		Ireland	25 (342/1368)	CM, PCR	*ssu*-rRNA, *gp60*	*C. parvum* (84), *C. bovis* (4), *C. ryanae* (1)	IIa (265)	De Waele et al. [130]
		Ireland	66 (68/103)	PCR^1^	*ssu*-rRNA, *gp60*	*C. ryanae* (36), *C. bovis* (23), *C. parvum* (16), *C. andersoni* (4), *Cryptosporidium* pig genotype^5^ (1), *Cryptosporidium* spp. (19), *C. bovis + C. xiaoi* (17), *C. parvum + C. hominis* (3), *C. ryanae + C. bovis* (2)	IIa (9)	Mirhashemi et al. [131]
		United Kingdom	16 (16/101)	CM, PCR	*ssu*-rRNA	*C. andersoni* (7)	–	Robinson et al. [132]
		Serbia/Montenegro	60 (62/103)	PCR	*ssu*-rRNA, *cowp*, *gp60*	*C. parvum* (62)	IIa (38), IIj (21), IId (3)	Misic and Abe [133]
		Spain	14 (41/291)	CM, PCR	*hsp70*	*C. parvum* (41)	–	Castro-Hermida et al. [134]
		Spain	8 (32/379)	CM, PCR	*ssu*-rRNA, *hsp70*	*C. parvum* (10)	–	Castro-Hermida et al. [94]
		Spain	58 (166/287)	CM, PCR	*ssu*-rRNA, *gp60*	*C. parvum* (147), *C. bovis* (2)	IIa (138), IId (2)	Quilez et al. [135]
		Spain	49 (30/61)	CM, PCR	*ssu*-rRNA, *gp60*	*C. parvum* (27)	IIa (27)	Díaz et al. [136]
		Spain	15 (97/649)	CM, PCR	*ssu*-rRNA	*C. parvum* (41) *C. andersoni* (17)	–	Castro-Hermida et al. [96]
		Spain	94 (131/140)	PCR^1^	*ssu*-rRNA, *gp60*	*C. parvum* (131)	IIa (129), IId (2)	Quílez et al. [137]
		Spain	12 (45/362)	PCR	*ssu*-rRNA	*C. felis* (4), *C. bovis* (2), *Cryptosporidium* spp. (3)	–	Cardona et al. [95]
		Spain	17 (99/594)	PCR	*ssu*-rRNA, *gp60*	*C. parvum* (42), *C. bovis* (36), *C. ryanae* (10), *C. occultus* (7), *C. andersoni* (2), *C. xiaoi* (1)	IIa (30)	Díaz et al. [138]
		Belgium	19 (155/832)	CM, PCR	*ssu*-rRNA, *hsp70*	*C. parvum* (105), *C. bovis* (9), *C. suis* (1)	IIa (89), IId (1)	Geurden et al. [139]
		Belgium	18 (63/355)	PCR	*ssu*-rRNA, *gp60*	*C. parvum* (45), *C. bovis* (14), *C. ryanae* (4)	IIa (39)	Pinto et al. [122]
		Hungary	49 (39/79)	CM, PCR	*ssu*-rRNA, *gp60*	*C. parvum* (21), *Cryptosporidium* deer-like genotype^4^ (1)	IIa (19), IId (2)	Plutzer et al. [140]
		Germany	36 (48/134)	CM, PCR	*ssu*-rRNA, *cowp*, *gp60*	*C. parvum* (53)	IIa (52), IId (1)	Broglia et al. [141]
		Germany	89 (455/512)	CM, PCR	*ssu*-rRNA, *gp60*	*C. parvum* (395)	IIa (395)	Holzhausen et al. [142]
		Germany	87 (233/268)	PCR^1^	*ssu*-rRNA, *gp60*	*C. parvum* (233)	IIa (226)	Göhring et al. [143]
		Slovenia	ND (51/ND)	PCR	*ssu*-rRNA, *gp60*	*C. parvum* (45), *C. bovis* (3), *C. ryanae*	IIa (41), IIl (4)	Soba and Logar [144]
		Italy	8 (155/2024)	CM, PCR	*cowp*, *gp60*	*C. parvum* (101)	IIa (62)	Duranti et al. [145]
		Italy	100 (122/122)	PCR^1^	*cowp*	*C. parvum* (122)	–	Drumo et al. [146]
		Italy	39 (57/147)	CM, PCR	*ssu*-rRNA, *gp60*	*C. parvum* (46), *C. bovis* (2)	IIa (33), IId (3)	Díaz et al. [147]
		France	35 (147/422)	PCR	*ssu*-rRNA, *gp60*	*C. parvum* (60), *C. ryanae* (39), *C. bovis* (37), *C. ubiquitum* (1)	IIa (51)	Follet et al. [148]
		France	46 (84/182)	CM, PCR	*ssu*-rRNA, *gp60*	*C. ryanae* (22), *C. bovis* (15), *C. parvum* (14), *C. bovis + C. ryanae* (5), *C. bovis + C. parvum* (4), *C. parvum + C. ryanae* (1)	IIa (15)	Rieux et al. [149]
		France	31 (92/300)	CM, PCR	*ssu*-rRNA, *gp60*	*C. parvum* (80), *C. bovis* (2)	IIa (52)	Rieux et al. [150]
		France	36 (32/88)	CM, PCR	*ssu*-rRNA	*C. bovis* (27), *C. ryanae* (4), *C. parvum* (1)	–	Rieux et al. [151]
		France	64 (201/312)	CM, PCR	*ssu*-rRNA, *gp60*	*C. parvum* (80), *C. bovis* (53), *C. ryanae* (19), *C. bovis + C. parvum* (4), *C. bovis + C. ryanae* (4)	IIa (51)	Rieux et al. [152]
		France	21 (86/412)	CM, PCR	*ssu*-rRNA, *gp60*	*C. parvum* (71), *C. hominis* (15)	IIa (71), Ib (15)	Razakandrainibe et al. [153]
		France	89 (31/35)	CM, PCR	*ssu*-rRNA, *gp60*	*C. parvum* (30)	IIa (24), IId (3)	Mammeri et al. [154]
		France	23 (79/350)	PCR	*ssu*-rRNA, *gp60*	*C. parvum* (51), *C. bovis* (22), *C. ryanae* (4), *C. andersoni* (1), *C. xiaoi* (1)	IIa (47)	Pinto et al. [122]
		Romania	25 (65/258)	CM, PCR	*ssu*-rRNA, *gp60*	*C. parvum* (65)	IIa (13)	Imre et al. [155]
		Romania	59 (17/29)	PCR^1^	*ssu*-rRNA, *gp60*	*C. parvum* (17)	IIa (1), IId (16)	Vieira et al. [156]
		Sweden	63 (110/176)	PCR^1^	*ssu*-rRNA, *gp60*	*C. bovis* (83), *C. parvum* 15), *C. ryanae* (10), *C. andersoni* (2)	IIa (7), IId (6)	Silverlås et al. [157]
		Sweden	31 (242/782)	CM, PCR	*ssu*-rRNA, *gp60*	*C. parvum* (171), *C. bovis* (7), *C. bovis+ C. parvum* (5)	IIa (136), IId (40)	Silverlås et al. [158]
		Sweden	ND (ND/480)	CM, PCR	*ssu*-rRNA, *gp60*	*C. bovis* (48), *C. ryanae* (11), *C. parvum* (2)	IIa (2)	Silverlås and Blanco-Penedo [159]
		Sweden	37 (122/332)	CM, PCR	*ssu*-rRNA, *gp60*	*C. bovis* (72), *C. ryanae* (13), *C. parvum* (8), *C. ubiquitum* (1), *C. bovis + C. parvum* (13), *C. ryanae + C. parvum* (6)	IIa (7), IId (16)	Björkman et al. [160]
		Sweden	39 (92/238)	CM, PCR	*ssu*-rRNA, *gp60*	*C. bovis* (63), *C. ryanae* (7), *C. bovis* + *C. ryanae* (2)	NA	Åberg et al. [161]
		Sweden	22 (99/455)	CM, PCR	*ssu*-rRNA, *gp60*	*C. bovis* (46), *C. ryanae* (7), *C. bovis* + *C. ryanae* (2)	NA	Åberg et al. [162]
		Estonia	18 (9/49)	CM, PCR	*ssu*-rRNA, *hsp70*, *gp60*	*C. parvum* (1)	IIa (1)	Lassen et al. [163]
		Estonia	23 (110/486)	PCR	*ssu*-rRNA, *gp60*	*C. parvum* (105), *C. bovis* (4), *C. ryanae* (1)	IIa (90), IIl (5)	Santoro et al. [164]
		Slovakia	14 (14/100)	PCR	*ssu*-rRNA, *gp60*	*C. parvum* (10), *C. bovis* (4)	IIa (10)	Danišová et al. [165]
		Austria	55 (98/177)	CM, PCR	*gp60*	*C. parvum* (37)	IIa (35)	Lichtmannsperger et al. [100]
		Austria	55 (98/177)	CM, PCR	*ssu*-rRNA, *gp60*	*C. parvum* (67), *C. ryanae* (11), *C. bovis* (7)	IIa (65)	Lichtmannsperger et al. [166]
		Cyprus	44 (106/242)	PCR	*ssu*-rRNA, *gp60*	*C. parvum* (50), *C. bovis* (23), *C. ryanae* (28), *C. ryanae + C. parvum* (5)	IIa (47)	Hoque et al. [167]
		Latvia	34 (313/926)	CM, PCR	*ssu*-rRNA, *gp60*	*C. parvum* (62), *C. bovis* (29), *C. andersoni* (22), *C. ryanae* (11), *C. scrofarum* (1), *C. ubiquitum* (1), *C. parvum + C. bovis* (3), *C. bovis + C. ryanae* (3), *C. parvum* + *C. andersoni* (1), *C. parvum + C. ryanae* (1), *C. bovis + C. andersoni* (1)	IIa (55), IId (3)	Deksne et al. [168]
	*Capra hircus*	Czech Republic	100 (1/1)	PCR^1^	*ssu*-rRNA, *cowp*	*C. parvum* (1)	–	Hajdušek et al. [46]
		Spain	40 (2/5)	CM, PCR	*ssu*-rRNA	*C. xiaoi* (2)	–	Díaz et al. [169]
		Spain	100 (17/17)	PCR^1^	*ssu*-rRNA, *gp60*	*C. parvum* (17)	IId (17)	Quílez et al. [135]
		Spain	63 (74/118)	CM, PCR	*ssu*-rRNA, *gp60*	*C. parvum* (60), *C. xiaoi* (4), *C. parvum + C. xiaoi* (1)	IIa (55), IId (3)	Díaz et al. [170]
		Spain	6 (14/234)	PCR	*ssu*-rRNA, *gp60*	*C. parvum* (3), *C. ubiquitum* (5), *C. xiaoi* (5)	IIa (1), IId (2), XIIa (3)	Díaz et al. [171]
		Belgium	10 (14/148)	CM, PCR	*ssu*-rRNA, *hsp70*, *gp60*	*C. parvum* (11)	IIa (3), IId (8)	Geurden et al. [103]
		The Netherlands	100 (1/1)	PCR^1^	*hsp70*, *gp60*, ML1	*C. parvum* (1)	IIa (1)	Wielinga et al. [121]
		Italy	100 (21/21)	PCR^1^	*cowp*	*C. parvum* (21)	–	Drumo et al. [146]
		Norway	21 (4/19)	CM, PCR	*ssu*-rRNA, LIB13, *gp60*	*C. parvum* (3), *C. xiaoi* (1)	IIa (3)	Lange et al. [172]
		France	24 (61/254)	CM, PCR	*ssu*-rRNA, *gp60*	*C. xiaoi* (18), *C. parvum* (1)	NA	Rieux et al. [173]
		France	NA (22/ND)	CM, PCR	*ssu*-rRNA	*C. ubiquitum* (12)	–	Paraud et al. [174]
		Greece	7 (18/255)	CM, PCR	*ssu*-rRNA, *hsp70*, *gp60*	*C. xiaoi* (7), *C. ubiquitum* (5), *C. parvum* (2)	IId (2)	Tzanidakis et al. [104]
		Greece	28 (41/148)	CM, PCR	*ssu*-rRNA, *gp60*	*C. parvum* (16), *C. xiaoi* (1)	IIa (5), IId (11)	Papanikolopoulou et al. [175]
		Poland	37 (39/105)	PCR	*ssu*-rRNA, *gp60*	*C. xiaoi* (29), *C. parvum* (1)	IId (1)	Kaupke et al. [176]
	*Equus caballus*	Czech Republic	100 (3/3)	PCR^1^	*ssu*-rRNA, *cowp*	*C. parvum* (3)	–	[46]
		Czech Republic/Poland	3 (12/352)	PCR	*ssu*-rRNA, *gp60*	*C. muris* (9), *C. parvum* (1), *C. tyzzeri* (1), *Cryptosporidium* horse genotype (1)	IIa (1), IXb (1), VIa (1)	Wagnerová et al. [177]
		Italy	8 (12/540)	CM, PCR	*cowp*	*C. parvum* (12)	–	Veronesi et al. [105]
		Italy	2 (4/185)	CM, PCR	*cowp*	*C. parvum* genotype II (4)	–	Perrucci et al. [178]
		Italy	38 (14/37)	PCR	*ssu*-rRNA, *gp60*	*Cryptosporidium* horse genotype (11), *C. parvum* (3)	VIa (9)	Caffara et al. [179]
		Italy	17 (35/205)	CM, PCR	*ssu*-rRNA, *gp60*	*C. parvum* (5), *Cryptosporidium*. horse genotype (21), *C. parvum* + *C*. horse genotype (9)	IIa (6), IId (2), VIa (20)	Galuppi et al. [180]
		Belgium	5 (6/134)	CM, PCR	*ssu*-rRNA, *hsp70*	*Cryptosporidium* horse genotype (6)	–	Kostopoulou et al. [107]
		Greece	1 (2/190)	CM, PCR	*ssu*-rRNA, *hsp70*	*Cryptosporidium* horse genotype (2)	–	Kostopoulou et al. [107]
		Ireland	56 (23/41)	PCR^1^	*ssu*-rRNA, *gp60*	*C. ryanae* (17), *C. parvum* (4), *C. bovis* (1), *Cryptosporidium* horse genotype (1), *C*. pig genotype^6^ (1), *C. bovis + C. xiaoi* (5), *C. andersoni + C. bovis* (1), *C. andersoni + C. bovis* (1)	IIj (1)	Mirhashemi et al. [131]
		Portugal	21 (3/14)	CM, PCR	*ssu*-rRNA, *hsp70*	*C. parvum* (1)	IIa (1)	Couso-Pérez et al. [181]
		Spain	11 (7/65)	CM, PCR	*ssu*-rRNA, *hsp70*	*C. parvum* (6)	IIa (2)	Couso-Pérez et al. [181]
	*Ovis aries*	Poland	10 (16/159)	CM, PCR	*ssu*-rRNA	*C*. spp. (10)	–	Majewska et al. [182]
		Poland	19 (45/234)	PCR	*ssu*-rRNA, *gp60*	*C. xiaoi* (30), *C. bovis* (9), *C. ubiquitum* (3), *C. xiaoi* + *C. parvum + C. hominis* (1), *C. xiaoi* + *C. parvum* (1), *C. xiaoi* + *Cryptosporidium* spp. (1)	IIa (2)	Kaupke et al. [176]
		Denmark	100 (1/1)	PCR^1^	*cowp*, *ssu*-rRNA	*C. parvum* (1)	–	Enemark et al. [127]
		Portugal	100 (2/2)	PCR^1^	*ssu*-rRNA, *gp60*	*C. parvum* (2)	IIa (1), IId (1)	Alves et al. [125]
		Belgium	13 (18/137)	CM, PCR	*ssu*-rRNA, *hsp70*, *gp60*	*Cryptosporidium* cervine genotype^7^ (9), *C. parvum* (1)	IIa (1)	Geurden et al. [103]
		United Kingdom	43 (127/297)	CM, PCR	*ssu*-rRNA, *cowp*	*C. parvum* (52), *C. bovis* (5), *Cryptosporidium* cervine genotype^7^ (1)	–	Mueller-Doblies et al. [183]
		Italy	17 (26/149)	CM, PCR	*cowp*	*C. parvum* (26)	–	Paoletti et al. [184]
		Italy	100 (21/21)	PCR^1^	*cowp*	*C. parvum* (21)	–	Drumo et al. [146]
		Italy	4 (1/27)	CM, PCR	*gp60*	*C. parvum* (4)	IIa (4)	Cacciò et al. [185]
		Italy	10 (92/915)	CM, PCR	*ssu*-rRNA, *gp60*	*C. parvum* (11), *C. ubiquitum* (4)	IIa (5), IId (1), XIIa (4)	Dessì et al. [186]
		The Netherlands	100 (1/1)	PCR^1^	*hsp70*, *gp60*, ML1	*C. parvum* (1)	IIa (1)	Wielinga et al. [121]
		Spain	100 (131/131)	PCR^1^	*ssu*-rRNA, *gp60*	*C. parvum* (131)	IIa (3), IId (128)	Quílez et al. [187]
		Spain	31 (39/127)	CM, PCR	*ssu*-rRNA, *gp60*	*C. parvum* (14), *Cryptosporidium* cervine genotype^7^ (9)	IIa (12)	Díaz et al. [136]
		Spain	19 (42/227)	CM, PCR	*ssu*-rRNA	*C. parvum* (27)	–	Castro-Hermida et al. [96]
		Spain	32 (54/171)	CM, PCR	*ssu*-rRNA, *gp60*	*C. parvum* (31), *C. ubiquitum* (10), *C. parvum + C. ubiquitum* (1)	IIa (26)	Díaz et al. [170]
		Spain	6 (19/324)	PCR	*ssu*-rRNA, *gp60*	*C. parvum* (13), *C. ubiquitum* (1), *C. xiaoi* (3)	IIa (8), XIIa (1)	Díaz et al. [171]
		Norway	15 (160/1095)	CM, PCR	*ssu*-rRNA, actin	*Cryptosporidium* cervine genotype^7^ (35), *C. xiaoi* (7)	–	Robertson et al. [112]
		Norway	100 (2/2)	CM, PCR	*ssu*-rRNA, LIB13, *gp60*	*C. parvum* (1), *C. xiaoi* (1)	IIa (2)	Lange et al. [172]
		Romania	14 (24/175)	CM, PCR	*ssu*-rRNA, *gp60*	*C. parvum* (20), *C. ubiquitum* (2), *C. xiaoi + C. bovis* (2)	IIa (3), IId (4)	Imre et al. [188]
		Greece	5 (22/429)	CM, PCR	*ssu*-rRNA, *hsp70*, *gp60*	*C. parvum* (7), *C. ubiquitum* (3), *C. parvum* (7)	IId (7)	Tzanidakis et al. [104]
		Greece	30 (39/132)	CM, PCR	*ssu*-rRNA, *gp60*	*C. parvum* (16)	IIa (9), IId (4)	Papanikolopoulou et al. [175]
		Ireland	49 (51/104)	PCR^1^	*ssu*-rRNA, *gp60*	*C. parvum* (14), *C. xiaoi* (10), *C. ryanae* (9), *C. ubiquitum* (7), *C. bovis* (1), *Cryptosporidium* spp. (15), *C. bovis + xiaoi* (18)	IIa (6)	Mirhashemi et al. [131]
		France	100 (23/23)	CM, PCR	*ssu*-rRNA, *gp60*	*C. parvum* (23)	IIa (23)	Mammeri et al. [189]
		France	35 (53/151)	CM, PCR	*ssu*-rRNA, LIB13, *gp60*	*C. parvum* (10), *C. xiaoi* (4), *C. ubiquitum* (1)	IIa (3), IId (2)	Bordes et al. [190]

*Note. bg*, *β*-giardin; CM, conventional microscopy; *cowp*, *Cryptosporidium* oocyst wall protein; *gdh*, glutamate dehydrogenase; *gp60*, 60-kDa glycoprotein; *hsp70*, 70-kDa heat shock protein; ITS, internal transcribed spacer; LIB13, protein of unknown function; NA, no amplification; ND, no data available; PCR, polymerase chain reaction; *ssu*-rRNA, small subunit ribosomal RNA; TRAP-C1, thrombospondin-related adhesive protein of *Cryptosporidium*-1; t*pi*, triose phosphate isomerase. ^1^PCR conducted only in samples with a previous positive result by another detection method. ^2^Primer sequences specifically designed to amplify assemblage D. ^3^Previous nomenclature adopted by the authors. ^4^Current *C. ryanae*. ^5^Current *C. suis*. ^6^Current *C. scrofarum*. ^7^Current *C. ubiquitum*.

**Table 3:** Prevalence of *Giardia duodenalis* and *Cryptosporidium* spp. according to the host species and geographical origin found in the present study.

Variables	No. of samples examined	*Giardia duodenalis* (%)	95% CI	*P*-value	*Cryptosporidium* spp. (%)	95% CI	*P*-value
Hosts				**0.008**			**0.005**
Wild carnivores							
Iberian wolf (*Canis lupus signatus*)	121	31 (25.6)	18.1–34.4		3 (2.5)	0.5–7.1	
Iberian lynx (*Lynx pardinus*)	30	8 (26.7)	12.3–45.9		1 (3.3)	0.1–17.2	
Red fox (*Vulpes vulpes*)	118	22 (18.6)	12.1–26.9		4 (3.4)	0.9–8.5	
Stone marten (*Martes foina*)	19	3 (15.8)	3.4–39.6		0	–	
Subtotal Wild ungulates	288	62 (21.5)	16.9–26.7		8 (2.8)	1.2–5.4	
Red deer (*Cervus elaphus*)	96	4 (4.2)	1.2–10.3		3 (3.1)	0.7–8.9	
Roe deer (*Capreolus capreolus*)	39	4 (10.3)	2.9–24.2		0	–	
Wild boar (*Sus scrofa*)	107	16 (15.0)	8.8–23.1		9 (8.4)	3.9–15.4	
Subtotal	242	24 (9.9)	6.5–14.4		12 (5.0)	2.6–8.5	
Domestic animals							
Dog (*Canis lupus familiaris*)	46	11 (23.9)	12.6–38.8		0	–	
Horse (*Equus caballus*)	26	2 (7.7)	1.0–25.1		0	–	
Cattle (*Bos taurus*)	87	13 (14.9)	8.2–24.2		0	–	
Sheep (*Ovis aries*)	46	8 (17.4)	7.8–31.4		0	–	
Goat (*Capra hircus*)	21	0	–		0	–	
Subtotal	226	34 (15.0)	10.7–20.4		0	–	
Study areas				**0.003**			**0.020**
Montesinho Natural Park	215	44 (20.5)	15.3–26.5		13 (6.0)	3.4–10.1	
Central Portugal West	249	48 (19.3)	14.6–24.7		2 (0.8)	0.1–2.9	
Central Portugal East	102	15 (14.7)	8.5–23.1		2 (2.0)	0.2–6.9	
Faia Brava Reserve	60	5 (8.3)	2.8–18.4		0	–	
Lousã Mountains	61	0	–		2 (3.3)	0.4–11.4	
Malcata Nature Reserve	39	2 (5.1)	0.6–17.3		0	–	
Guadiana Valley	30	8 (26.7)	12.3–45.9		1 (3.3)	0.1–17.2	
TOTAL	756	122 (16.1)	13.6–19.0		20 (2.7)	1.6–4.1	

*Note*. 95% confidence intervals (CIs) are indicated. Values in bold represent statistical significance.

**Table 4:** Frequency and molecular diversity of *Giardia duodenalis* and *Cryptosporidium* spp. in wild and domestic animal species investigated in the present study.

Species	Host	Study area	Genotype	Isolates (*n*)	Locus	Reference sequence	Stretch	Single nucleotide polymorphisms (SNPs)	GenBank ID
*G. duodenalis*	Roe deer	MNP	A^a^	1	*ssu*-rRNA	M54878	1–289	A90C, A185R	OQ818646
	Dog	CPW	D	1	*ssu*-rRNA^b^	AF199449	1–248	None	OQ818647
	Iberian wolf	CPW	D	2	*ssu*-rRNA^b^	AF199449	1–293	None	OQ818648
	Iberian wolf	CPW	D	1	*ssu*-rRNA^b^	AF199449	16–284	C262Y	OQ818649
	Iberian wolf	CPW	C	2	*gdh*	U60984	76–491	C207T	OQ818103
	Iberian wolf	MNP	D	2	*gdh*	U60986	64–491	T240Y, C375Y, T429Y, G441R, T459W	OQ818104
	Iberian wolf	MNP	D	1	*gdh*	U60986	81–491	T429C, G441A	OQ818105
	Iberian wolf	CPW, MNP	D	2	*bg*	AY545647	97–604	A159R, A201G, C207Y	OQ818106
	Red fox	CPW	D	1	*ssu*-rRNA^b^	AF199449	10–293	None	OQ818650
	Red fox	CPW	C	1	*gdh*	U60984	76–491	None	OQ818107
	Red fox	CPW	C	1	*bg*	AY545646	4–499	C217T	OQ818108
	Cattle	CPW	E	1	*ssu*-rRNA^b^	AF199448	10–289	None	OQ818651
	Horse	FBR	E	1	*ssu*-rRNA^b^	AF199448	1–289	None	OQ818652
	Sheep	MNP, CPW	E	2	*ssu*-rRNA^b^	AF199448	1–289	None	OQ818653
	Wild boar	MNP	E	1	*ssu*-rRNA^b^	AF199448	1–289	None	OQ818654
*C. canis*	Iberian wolf	CPW, MNP	Unknown	3	*ssu*-rRNA	AF112576	525–1030	None	OQ818655
	Red fox	CPE, LM, MNP	Unknown	4	*ssu*-rRNA	AF112576	525–1030	686InsTG, T739A	OQ818656
*C. felis*	Iberian lynx	GV	Unknown	1	*ssu*-rRNA	AF108862	550–1029	C579T	OQ818657
*C. ryanae*	Red deer	MNP	Unknown	1	*ssu*-rRNA	MT835148	328–806	None	OQ818658
*C. scrofarum*	Wild boar	CPE, MNP	–	7	*ssu*-rRNA	KF597534	285–734	None	OQ818659
	Wild boar	MNP	–	1	*ssu*-rRNA	KF597534	328–726	438InsTT	OQ818660
*C. occultus*	Red deer	LM	–	1	*ssu*-rRNA	MG699179	323–824	484InsAT	OQ818661
*C. ubiquitum*	Red deer	MNP	Unknown	1	*ssu*-rRNA	KY052177	21–502	None	OQ818662
	Wild boar	MNP	Unknown	1	*ssu*-rRNA	KY052177	21–496	None	OQ818663

*Note: bg*, *β*-giardin; *gdh*, glutamate dehydrogenase; Ins, insertion; R, A/G; *ssu*-rRNA, small subunit ribosomal RNA; W, A/T; Y, C/T. ^a^Characterized as subassemblage AI. ^b^Taxonomy and reference sequences as proposed by Plutzer et al. [205]. Mentioned study areas: MNP, Montesinho Natural Park; CPW, Central Portugal West; CPE, Central Portugal East; GV, Guadiana Valley; LM, Lousã Mountains; FBR, Faia Brava Reserve.

## Data Availability

The authors confirm that the data supporting the findings of this study are available within the main body of the manuscript.

## Supplementary Materials

Supplementary 1 PCR cycling conditions used for molecular identification and/or characterization of *Giardia duodenalis*, *Cryptosporidium* spp., and *Balantioides coli* in the present study.

Supplementary 2 Oligonucleotides used for molecular identification and/or characterization of *Giardia duodenalis*, *Cryptosporidium* spp., and *Balantioides coli* in the present study.

Supplementary 3 Dataset showing sampling, epidemiological, diagnostic, and molecular data of the present study.
