# Supplementary material for: Molecular Detection and Distribution of Giardia duodenalis and Cryptosporidium spp. Infections in Wild and Domestic Animals in Portugal
Source: Transbound Emerg Dis. 2023 Nov 8;2023:5849842. doi: 10.1155/2023/5849842 (PMC12017001; doi:10.1155/2023/5849842)
Supplement: Supplementary 1 — PCR cycling conditions used for molecular identification and/or characterization of Giardia duodenalis, Cryptosporidium spp., and Balantioides coli in the present study. [file 5849842.f1.docx]

|  |  | **Temperature and time** | | | |  |  |  |
| --- | --- | --- | --- | --- | --- | --- | --- | --- |
| **Target pathogen** | **Locus** | **Initial denaturation** | **Denaturation** | **Annealing** | **Extension** | **No. cycles** | **Final extension** | **Reference** |
| *Giardia duodenalis* | *ssu* rRNA | 95°C 15 min | 95°C 15 s | 60°C 1 min | 72°C 30 s | 45 | – | Verweij et al. (2003) |
|  | *ssu* rRNA | 95°C 2 min | 95°C 45 s | 58/55°C 30 s | 72°C 45 s | 35 | 72°C 4 min | Appelbee et al. (2003) |
|  | *gdh* | 95°C 3 min | 95°C 30 s | 55°C 30 s | 72°C 1 min | 35 | 72°C 7 min | Read et al. (2004) |
|  | *bg* | 95°C 7 min | 95°C 30 s | 65/55°C 30 s | 72°C 1 min | 35 | 72°C 7 min | Lalle et al. (2005) |
|  | *tpi* | 94°C 5 min | 94°C 45 s | 50°C 45 s | 72°C 1 min | 35 | 72°C 10 min | Sulaiman et al. (2003) |
| *Cryptosporidium* spp. | *ssu* rRNA | 94°C 3 min | 94°C 40 s | 50°C 40 s | 72°C 1 min | 35 | 72°C 10 min | Tiangtip and Jongwutiwes (2002) |
| *Cryptosporidium canis* | *gp60* | 94°C 5 min | 94°C 45 s | 52/50°C 45 s | 72°C 80 s | 35 | 72°C 10 min | Jiang et al. (2021) |
| *Cryptosporidium felis* | *gp60* | 95ºC 4 min | 95ºC 30 s | 55ºC 30 s | 72º C 90 s | 35 | 72º C 7 min | Rojas-Lopez et al. (2020) |
| *Cryptosporidium ryanae* | *gp60* | 94°C 5 min | 94°C 45 s | 55°C 45 s | 68°C 1 min | 35 | 68°C 10 min | Yang et al. (2020) |
| *Cryptosporidium ubiquitum* | *gp60* | 94°C 5 min | 94°C 45 s | 58/55°C 45 s | 72°C 1 min | 35 | 72°C 7 min | Li et al. (2014) |
| *Balantioides coli* | ITS | 94°C 10 min | 94°C 1 min | 60°C 1 min | 72°C 1 min | 30 | 72°C 5 min | Ponce-Gordo et al. (2011) |

**Supplementary Table** **S1**. PCR cycling conditions used for molecular identification and/or characterisation of *Giardia duodenalis, Cryptosporidium* spp. and *Balantioides coli* in the present study.

*bg*: β-giardin; *gdh*: Glutamate dehydrogenase; *gp60*: 60 kDa glycoprotein; *ssu* rRNA: Small subunit ribosomal RNA; *tpi*: Triose phosphate isomerase; ITS: Internal transcribed spacer.
