# Supplementary material for: Molecular Detection and Distribution of Giardia duodenalis and Cryptosporidium spp. Infections in Wild and Domestic Animals in Portugal
Source: Transbound Emerg Dis. 2023 Nov 8;2023:5849842. doi: 10.1155/2023/5849842 (PMC12017001; doi:10.1155/2023/5849842)
Supplement: Supplementary 2 — Oligonucleotides used for molecular identification and/or characterization of Giardia duodenalis, Cryptosporidium spp., and Balantioides coli in the present study. [file 5849842.f2.docx]

**Supplementary Table** **S2**. Oligonucleotides used for molecular identification and/or characterisation of *Giardia duodenalis*, *Cryptosporidium* spp. and *Balantioides coli* in the present study.

| **Target pathogen** | **Locus** | **Oligonucleotide** | **Sequence (5´–3´)** | **Reference** |
| --- | --- | --- | --- | --- |
| *Giardia duodenalis* | *ssu* rRNA | Probe | FAM–CCCGCGGCGGTCCCTGCTAG–BHQ1 | Verweij et al. (2003) |
|  |  | Gd-80F | GACGGCTCAGGACAACGGTT |  |
|  |  | Gd-127R | TTGCCAGCGGTGTCCG |  |
|  | *ssu* rRNA | Gia2029 | AAGTGTGGTGCAGACGGACTC | Appelbee et al. (2003) |
|  |  | Gia2150c | CTGCTGCCGTCCTTGGATGT |  |
|  |  | RH11 | CATCCGGTCGATCCTGCC | Hopkins et al. (1997) |
|  |  | RH4 | AGTCGAACCCTGATTCTCCGCCAGG |  |
|  | *gdh* | GDHeF | TCAACGTYAAYCGYGGYTTCCGT | Read et al. (2004) |
|  |  | GDHiF | CAGTACACCTCYGCTCTCGG |  |
|  |  | GDHiR | GTTRTCCTTGCACATCTCC |  |
|  | *bg* | G7_F | AAGCCCGACGACCTCACCCGCAGTGC | Lalle et al. (2005) |
|  |  | G759_R | GAGGCCGCCCTGGATCTTCGAGACGAC |  |
|  |  | G99_F | GAACGAACGAGATCGAGGTCCG |  |
|  |  | G609_R | CTCGACGAGCTTCGTGTT |  |
|  | *tpi* | AL3543 | AAATIATGCCTGCTCGTCG | Sulaiman et al. (2003) |
|  |  | AL3546 | CAAACCTTITCCGCAAACC |  |
|  |  | AL3544 | CCCTTCATCGGIGGTAACTT |  |
|  |  | AL3545 | GTGGCCACCACICCCGTGCC |  |
| *Cryptosporidium* spp. | *ssu* rRNA | CR-P1 | CAGGGAGGTAGTGACAAGAA | Tiangtip and Jongwutiwes (2002) |
|  |  | CR-P2 | TCAGCCTTGCGACCATACTC |  |
|  |  | CR-P3 | ATTGGAGGGCAAGTCTGGTG |  |
|  |  | CPB-DIAGR | TAAGGTGCTGAAGGAGTAAGG |  |
| *Cryptosporidium canis* | *gp*60 | GP60-Canis-F1 | ATACTCTGGTCTCCCGTTT | Jiang et al. (2021) |
|  |  | GP60-Canis-R1 | GTACTCGGAAGCGGTGTA |  |
|  |  | GP60-Canis-F2 | AAGGCGCCTCACTCATT |  |
|  |  | GP60-Canis-R2 | TCAGTTAGATATCACCCATTAA |  |
| *Cryptosporidium felis* | *gp60* | CF_F1 | TTT CCG TTA TTG TTG CAG TTG CA | Rojas-Lopez et al. (2020) |
|  |  | CF_R1 | ATC GGA ATC CCA CCA TCG AAC |  |
|  |  | CF_F2 | GGG GGT TCT GAA GGA TGT AA |  |
|  |  | CF_R2 | CGG TGG TCT CCT CAG TCT TC |  |
| *Cryptosporidium ryanae* | *gp*60 | Ry-gp60-F1 | GCTCGAGTTCTGAGTCGA | Yang et al. (2020) |
|  |  | Ry-gp60-F2 | CCTCAGATAATGAGCAGTCTA |  |
|  |  | Ry-gp60-F3 | TCTACCGTTCAGACTGAAGCT |  |
|  |  | Ry-gp60-F4 | AGTTCTGATTCAAGTAACGGTGA |  |
|  |  | Ry-gp60-F5 | GTCGACCTCAGGTAATGAGCA |  |
|  |  | Ry-gp60-F6 | GTCTAGTTCTTCTGATCAAGTTG |  |
|  |  | Ry-gp60-R1 | ATACCGTTAAAATGAAGGCCAA |  |
|  |  | Ry-gp60-R2 | GATGGGATAACATATCTATAACCAAA |  |
| *Cryptosporidium ubiquitum* | *gp*60 | Ubi-18S-F1 | TTTACCCACACATCTGTAGCGTCG | Li et al. (2014) |
|  |  | Ubi-18S-R1 | ACGGACGGAATGATGTATCTGA |  |
|  |  | Ubi-18S-F2 | ATAGGTGATAATTAGTCAGTCTTTAAT |  |
|  |  | Ubi-18S-R2 | TCCAAAAGCGGCTGAGTCAGCATC |  |
| *Balantioides coli* | ITS | B5D | GCTCCTACCGATACCGGGT | Ponce-Gordo et al. (2011) |
|  |  | B5RC | GCGGGTCATCTTACTTGATTTC |  |

*bg*: β-giardin; *gdh*: Glutamate dehydrogenase; *gp60*: 60 kDa glycoprotein; *ssu* rRNA: Small subunit ribosomal RNA; *tpi*: Triose phosphate isomerase; ITS: Internal transcribed spacer.
