# Supplementary material for: Molecular Detection and Distribution of Giardia duodenalis and Cryptosporidium spp. Infections in Wild and Domestic Animals in Portugal
Source: Transbound Emerg Dis. 2023 Nov 8;2023:5849842. doi: 10.1155/2023/5849842 (PMC12017001; doi:10.1155/2023/5849842)
Supplement: Supplementary 3 — Dataset showing sampling, epidemiological, diagnostic, and molecular data of the present study. [file 5849842.f3.pdf]

Supplementary Table S3 - Dataset showing sampling, epidemiological, diagnostic and molecular data of the present study.

Sample Information				Molecular methods for *Giardia duodenalis* detection and characterization																		
Sample ID	Study area	Host (common name)	Host (scientific name)	*Giardia* qPCR	Ct	*ssu*-PCR	*ssu* sequencing	SNPS *ssu*	Consensus sequence *ssu*	*gdh*-PCR	*gdh* sequencing	SNPS *gdh*	Consensus sequence *gdh*	*bg*-PCR	*bg* sequencing	SNPs *bg*	Consensus sequence *bg*	tpi-PCR	*tpi* sequencing	SNPs *tpi*	Consensus sequence *tpi*	Assigned genotype
L6	MNP	Iberian wolf	*Canis lupus signatus*	Negative	–	–	–	–	–	–	–	–	–	–	–	–	–	–	–	–	–	–
L7	MNP	Iberian wolf	*Canis lupus signatus*	Negative	–	–	–	–	–	–	–	–	–	–	–	–	–	–	–	–	–	–
L8	MNP	Iberian wolf	*Canis lupus signatus*	Negative	–	–	–	–	–	–	–	–	–	–	–	–	–	–	–	–	–	–
L9	MNP	Iberian wolf	*Canis lupus signatus*	**Positive**	34.1	Negative	–	–	–	Not done	–	–	–	Not done	–	–	–	Not done	–	–	–	Unknown
L11	MNP	Iberian wolf	*Canis lupus signatus*	**Positive**	35.1	Not done	–	–	–	Not done	–	–	–	Not done	–	–	–	Not done	–	–	–	Unknown
L12	MNP	Iberian wolf	*Canis lupus signatus*	Negative	–	–	–	–	–	–	–	–	–	–	–	–	–	–	–	–	–	–
L21	MNP	Iberian wolf	*Canis lupus signatus*	Negative	–	–	–	–	–	–	–	–	–	–	–	–	–	–	–	–	–	–
L23	MNP	Iberian wolf	*Canis lupus signatus*	Negative	–	–	–	–	–	–	–	–	–	–	–	–	–	–	–	–	–	–
L25	MNP	Iberian wolf	*Canis lupus signatus*	**Positive**	38.6	Not done	–	–	–	Not done	–	–	–	Not done	–	–	–	Not done	–	–	–	Unknown
L27	MNP	Iberian wolf	*Canis lupus signatus*	Negative	–	–	–	–	–	–	–	–	–	–	–	–	–	–	–	–	–	–
L28	MNP	Iberian wolf	*Canis lupus signatus*	Negative	–	–	–	–	–	–	–	–	–	–	–	–	–	–	–	–	–	–
L29	MNP	Iberian wolf	*Canis lupus signatus*	Negative	–	–	–	–	–	–	–	–	–	–	–	–	–	–	–	–	–	–
L31	MNP	Iberian wolf	*Canis lupus signatus*	**Positive**	33.3	Negative	–	–	–	Not done	–	–	–	Not done	–	–	–	Not done	–	–	–	Unknown
L32	MNP	Iberian wolf	*Canis lupus signatus*	**Positive**	35.8	Not done	–	–	–	Not done	–	–	–	Not done	–	–	–	Not done	–	–	–	Unknown
L34	MNP	Iberian wolf	*Canis lupus signatus*	Negative	–	–	–	–	–	–	–	–	–	–	–	–	–	–	–	–	–	–
L36	MNP	Iberian wolf	*Canis lupus signatus*	Negative	–	–	–	–	–	–	–	–	–	–	–	–	–	–	–	–	–	–
L37	MNP	Iberian wolf	*Canis lupus signatus*	Negative	–	–	–	–	–	–	–	–	–	–	–	–	–	–	–	–	–	–
L40	MNP	Iberian wolf	*Canis lupus signatus*	**Positive**	35.9	Not done	–	–	–	–	–	–	–	–	–	–	–	–	–	–	–	–
L44	MNP	Iberian wolf	*Canis lupus signatus*	Negative	–	–	–	–	–	Not done	–	–	–	Not done	–	–	–	Not done	–	–	–	Unknown
L46	MNP	Iberian wolf	*Canis lupus signatus*	Negative	–	–	–	–	–	–	–	–	–	–	–	–	–	–	–	–	–	–
L47	MNP	Iberian wolf	*Canis lupus signatus*	**Positive**	32.5	Negative	–	–	–	Not done	–	–	–	Not done	–	–	–	Not done	–	–	–	Unknown
L49	MNP	Iberian wolf	*Canis lupus signatus*	Negative	–	–	–	–	–	–	–	–	–	–	–	–	–	–	–	–	–	–
J1	MNP	Wild boar	*Sus scrofa*	Negative	–	–	–	–	–	–	–	–	–	–	–	–	–	–	–	–	–	–
J3	MNP	Wild boar	*Sus scrofa*	Negative	–	–	–	–	–	–	–	–	–	–	–	–	–	–	–	–	–	–
C2	MNP	Roe deer	*Capreolus capreolus*	Negative	–	–	–	–	–	–	–	–	–	–	–	–	–	–	–	–	–	–
V1	MNP	Red deer	*Cervus elaphus*	Negative	–	–	–	–	–	–	–	–	–	–	–	–	–	–	–	–	–	–
V2	MNP	Red deer	*Cervus elaphus*	Negative	–	–	–	–	–	–	–	–	–	–	–	–	–	–	–	–	–	–
V3	MNP	Red deer	*Cervus elaphus*	Negative	–	–	–	–	–	–	–	–	–	–	–	–	–	–	–	–	–	–
V4	MNP	Red deer	*Cervus elaphus*	Negative	–	–	–	–	–	–	–	–	–	–	–	–	–	–	–	–	–	–
V5	MNP	Red deer	*Cervus elaphus*	Negative	–	–	–	–	–	–	–	–	–	–	–	–	–	–	–	–	–	–
V6	MNP	Red deer	*Cervus elaphus*	Negative	–	–	–	–	–	–	–	–	–	–	–	–	–	–	–	–	–	–
V9	MNP	Red deer	*Cervus elaphus*	Negative	–	–	–	–	–	–	–	–	–	–	–	–	–	–	–	–	–	–
V10	MNP	Red deer	*Cervus elaphus*	Negative	–	–	–	–	–	–	–	–	–	–	–	–	–	–	–	–	–	–
V11	MNP	Red deer	*Cervus elaphus*	Negative	–	–	–	–	–	–	–	–	–	–	–	–	–	–	–	–	–	–
V12	MNP	Red deer	*Cervus elaphus*	Negative	–	–	–	–	–	–	–	–	–	–	–	–	–	–	–	–	–	–
V13	MNP	Red deer	*Cervus elaphus*	Negative	–	–	–	–	–	–	–	–	–	–	–	–	–	–	–	–	–	–
V14	MNP	Red deer	*Cervus elaphus*	Negative	–	–	–	–	–	–	–	–	–	–	–	–	–	–	–	–	–	–
V16	MNP	Red deer	*Cervus elaphus*	Negative	–	–	–	–	–	–	–	–	–	–	–	–	–	–	–	–	–	–
V17	MNP	Red deer	*Cervus elaphus*	**Positive**	35.5	Not done	–	–	–	Not done	–	–	–	Not done	–	–	–	Not done	–	–	–	Unknown
V18	MNP	Red deer	*Cervus elaphus*	Negative	–	–	–	–	–	–	–	–	–	–	–	–	–	–	–	–	–	–
V21	MNP	Red deer	*Cervus elaphus*	Negative	–	–	–	–	–	–	–	–	–	–	–	–	–	–	–	–	–	–
V22	MNP	Red deer	*Cervus elaphus*	Negative	–	–	–	–	–	–	–	–	–	–	–	–	–	–	–	–	–	–
JAMNP1	MNP	Wild boar	*Sus scrofa*	Negative	–	–	–	–	–	–	–	–	–	–	–	–	–	–	–	–	–	–
JAMNP2	MNP	Wild boar	*Sus scrofa*	Negative	–	–	–	–	–	–	–	–	–	–	–	–	–	–	–	–	–	–
JAMNP3	MNP	Wild boar	*Sus scrofa*	Negative	–	–	–	–	–	–	–	–	–	–	–	–	–	–	–	–	–	–
JAMNP4	MNP	Wild boar	*Sus scrofa*	Negative	–	–	–	–	–	–	–	–	–	–	–	–	–	–	–	–	–	–
JAMNP5	MNP	Wild boar	*Sus scrofa*	Negative	–	–	–	–	–	–	–	–	–	–	–	–	–	–	–	–	–	–
JAMNP6	MNP	Wild boar	*Sus scrofa*	Negative	–	–	–	–	–	–	–	–	–	–	–	–	–	–	–	–	–	–
JAMNP7	MNP	Wild boar	*Sus scrofa*	Negative	–	–	–	–	–	–	–	–	–	–	–	–	–	–	–	–	–	–
JAMNP8	MNP	Wild boar	*Sus scrofa*	Negative	–	–	–	–	–	–	–	–	–	–	–	–	–	–	–	–	–	–
JAMNP9	MNP	Wild boar	*Sus scrofa*	Negative	–	–	–	–	–	–	–	–	–	–	–	–	–	–	–	–	–	–
JAMNP10	MNP	Wild boar	*Sus scrofa*	Negative	–	–	–	–	–	–	–	–	–	–	–	–	–	–	–	–	–	–
JAMNP11	MNP	Wild boar	*Sus scrofa*	Negative	–	–	–	–	–	–	–	–	–	–	–	–	–	–	–	–	–	–
JAMNP12	MNP	Wild boar	*Sus scrofa*	Negative	–	–	–	–	–	–	–	–	–	–	–	–	–	–	–	–	–	–
JAMNP13	MNP	Wild boar	*Sus scrofa*	**Positive**	35.6	Not done	–	–	–	Not done	–	–	–	Not done	–	–	–	Not done	–	–	–	Unknown
JAMNP14	MNP	Wild boar	*Sus scrofa*	Negative	–	–	–	–	–	–	–	–	–	–	–	–	–	–	–	–	–	–
JAMNP15	MNP	Wild boar	*Sus scrofa*	Negative	–	–	–	–	–	–	–	–	–	–	–	–	–	–	–	–	–	–
JAMNP16	MNP	Wild boar	*Sus scrofa*	**Positive**	33.2	Negative	–	–	–	Not done	–	–	–	Not done	–	–	–	Not done	–	–	–	Unknown
JAMNP17	MNP	Wild boar	*Sus scrofa*	Negative	–	–	–	–	–	–	–	–	–	–	–	–	–	–	–	–	–	–
JAMNP18	MNP	Wild boar	*Sus scrofa*	Negative	–	–	–	–	–	–	–	–	–	–	–	–	–	–	–	–	–	–
JAMNP19	MNP	Wild boar	*Sus scrofa*	Negative	–	–	–	–	–	–	–	–	–	–	–	–	–	–	–	–	–	–
JAMNP20	MNP	Wild boar	*Sus scrofa*	Negative	–	–	–	–	–	–	–	–	–	–	–	–	–	–	–	–	–	–
JAMNP21	MNP	Wild boar	*Sus scrofa*	Negative	–	–	–	–	–	–	–	–	–	–	–	–	–	–	–	–	–	–
JAMNP22	MNP	Wild boar	*Sus scrofa*	Negative	–	–	–	–	–	–	–	–	–	–	–	–	–	–	–	–	–	–
JAMNP23	MNP	Wild boar	*Sus scrofa*	**Positive**	32.3	Negative	–	–	–	Not done	–	–	–	Not done	–	–	–	Not done	–	–	–	Unknown
JAMNP24	MNP	Wild boar	*Sus scrofa*	Negative	–	–	–	–	–	–	–	–	–	–	–	–	–	–	–	–	–	–
JAMNP25	MNP	Wild boar	*Sus scrofa*	Negative	–	–	–	–	–	–	–	–	–	–	–	–	–	–	–	–	–	–
JAMNP26	MNP	Wild boar	*Sus scrofa*	Negative	–	–	–	–	–	–	–	–	–	–	–	–	–	–	–	–	–	–
JAMNP27	MNP	Wild boar	*Sus scrofa*	Negative	–	–	–	–	–	–	–	–	–	–	–	–	–	–	–	–	–	–
JAMNP28	MNP	Wild boar	*Sus scrofa*	Negative	–	–	–	–	–	–	–	–	–	–	–	–	–	–	–	–	–	–
VAMNP8	MNP	Cattle	*Bos taurus*	Negative	–	–	–	–	–	–	–	–	–	–	–	–	–	–	–	–	–	–
VAMNP9	MNP	Cattle	*Bos taurus*	Negative	–	–	–	–	–	–	–	–	–	–	–	–	–	–	–	–	–	–
VAMNP10	MNP	Cattle	*Bos taurus*	Negative	–	–	–	–	–	–	–	–	–	–	–	–	–	–	–	–	–	–
VAMNP11	MNP	Cattle	*Bos taurus*	Negative	–	–	–	–	–	–	–	–	–	–	–	–	–	–	–	–	–	–
VAMNP12	MNP	Cattle	*Bos taurus*	Negative	–	–	–	–	–	–	–	–	–	–	–	–	–	–	–	–	–	–
VAMNP13	MNP	Cattle	*Bos taurus*	Negative	–	–	–	–	–	–	–	–	–	–	–	–	–	–	–	–	–	–
VAMNP14	MNP	Cattle	*Bos taurus*	Negative	–	–	–	–	–	–	–	–	–	–	–	–	–	–	–	–	–	–
VAMNP15	MNP	Cattle	*Bos taurus*	Negative	–	–	–	–	–	–	–	–	–	–	–	–	–	–	–	–	–	–
VAMNP16	MNP	Cattle	*Bos taurus*	Negative	–	–	–	–	–	–	–	–	–	–	–	–	–	–	–	–	–	–
VAMNP17	MNP	Cattle	*Bos taurus*	**Positive**	39	Not done	–	–	–	Not done	–	–	–	Not done	–	–	–	Not done	–	–	–	Unknown
VAMNP18	MNP	Cattle	*Bos taurus*	Negative	–	–	–	–	–	–	–	–	–	–	–	–	–	–	–	–	–	–
VAMNP19	MNP	Cattle	*Bos taurus*	Negative	–	–	–	–	–	–	–	–	–	–	–	–	–	–	–	–	–	–
VAMNP20	MNP	Cattle	*Bos taurus*	Negative	–	–	–	–	–	–	–	–	–	–	–	–	–	–	–	–	–	–
VAMNP21	MNP	Cattle	*Bos taurus*	Negative	–	–	–	–	–	–	–	–	–	–	–	–	–	–	–	–	–	–
VAMNP22	MNP	Cattle	*Bos taurus*	**Positive**	32.6	Negative	–	–	–	Not done	–	–	–	Not done	–	–	–	Not done	–	–	–	Unknown
VAMNP23	MNP	Cattle	*Bos taurus*	**Positive**	36.03	Not done	–	–	–	Not done	–	–	–	Not done	–	–	–	Not done	–	–	–	Unknown
VAMNP24	MNP	Cattle	*Bos taurus*	**Positive**	35.5	Not done	–	–	–	Not done	–	–	–	Not done	–	–	–	Not done	–	–	–	Unknown
VAMNP25	MNP	Cattle	*Bos taurus*	Negative	–	–	–	–	–	–	–	–	–	–	–	–	–	–	–	–	–	–
VAMNP26	MNP	Cattle	*Bos taurus*	**Positive**	30.8	Negative	–	–	–	Negative	–	–	–	Negative	–	–	–	Negative	–	–	–	Unknown
VAMNP27	MNP	Cattle	*Bos taurus*	**Positive**	32.7	Negative	–	–	–	Not done	–	–	–	Not done	–	–	–	Not done	–	–	–	Unknown
VAMNP28	MNP	Cattle	*Bos taurus*	Negative	–	–	–	–	–	–	–	–	–	–	–	–	–	–	–	–	–	–
VAMNP29	MNP	Cattle	*Bos taurus*	Negative	–	–	–	–	–	–	–	–	–	–	–	–	–	–	–	–	–	–
VAMNP30	MNP	Cattle	*Bos taurus*	Negative	–	–	–	–	–	–	–	–	–	–	–	–	–	–	–	–	–	–
VAMNP31	MNP	Cattle	*Bos taurus*	Negative	–	–	–	–	–	–	–	–	–	–	–	–	–	–	–	–	–	–
VAMNP32	MNP	Cattle	*Bos taurus*	Negative	–	–	–	–	–	–	–	–	–	–	–	–	–	–	–	–	–	–
VAMNP33	MNP	Cattle	*Bos taurus*	Negative	–	–	–	–	–	–	–	–	–	–	–	–	–	–	–	–	–	–
RMNP1	MNP	Red fox	*Vulpes vulpes*	Negative	–	–	–	–	–	–	–	–	–	–	–	–	–	–	–	–	–	–
LMNP1	MNP	Iberian wolf	*Canis lupus signatus*	Negative	–	–	–	–	–	–	–	–	–	–	–	–	–	–	–	–	–	–
RMNP2	MNP	Red fox	*Vulpes vulpes*	Negative	–	–	–	–	–	–	–	–	–	–	–	–	–	–	–	–	–	–
LMNP2	MNP	Iberian wolf	*Canis lupus signatus*	Negative	–	–	–	–	–	–	–	–	–	–	–	–	–	–	–	–	–	–
LMNP3	MNP	Iberian wolf	*Canis lupus signatus*	**Positive**	27.8	Negative	–	–	–	**Positive**	**D**	T240Y, C375Y, T429Y, G441R, T459W	Yes	Negative	–	–	–	Negative	–	–	–	**D**
LMNP4	MNP	Iberian wolf	*Canis lupus signatus*	**Positive**	32.9	Negative	–	–	–	**Positive**	Untypable	–	–	Negative	–	–	–	Negative	–	–	–	Unknown
LMNP5	MNP	Iberian wolf	*Canis lupus signatus*	**Positive**	37.3	Not done	–	–	–	Not done	–	–	–	Not done	–	–	–	Not done	–	–	–	Unknown
LMNP6	MNP	Iberian wolf	*Canis lupus signatus*	**Positive**	24.7	Negative	–	–	–	**Positive**	**D**	T240Y, C375Y, T429Y, G441R, T459W	Yes	Negative	–	–	–	Negative	–	–	–	**D**
RMNP3	MNP	Red fox	*Vulpes vulpes*	**Positive**	35.7	Not done	–	–	–	Not done	–	–	–	Not done	–	–	–	Not done	–	–	–	Unknown

	Molecular methods for *Crytosporidium* spp. detection and characterization									Molecular methods for *Balantioides coli* detection.			
Sample ID	*Cryptosporidium ssu*-PCR	Sequencing	SNPs *ssu*	Consensus sequence *ssu*	*Cryptosporidium gp60*-PCR	Sequencing *gp60*	SNPs *gp60*	Consensus sequence *gp60*	Assigned genotype	*Balantioides coli* ITS-*PCR*	Sequencing ITS	Homology	Consensus sequence ITS
L6	Negative	*Sordaria fimicola*	–	–	–	–	–	–	–	Not done	–	–	–
L7	Negative	*Sordaria fimicola*	–	–	–	–	–	–	–	Not done	–	–	–
L8	Negative	*Sordaria macrospora*	–	–	–	–	–	–	–	Not done	–	–	–
L9	Negative	*Neospora caninum*	–	–	–	–	–	–	–	Not done	–	–	–
L11	Negative	*Preussia* sp.	–	–	–	–	–	–	–	Not done	–	–	–
L12	Negative	Uncultured eukaryote	–	–	–	–	–	–	–	Not done	–	–	–
L21	Negative	–	–	–	–	–	–	–	–	Not done	–	–	–
L23	Negative	–	–	–	–	–	–	–	–	Not done	–	–	–
L25	Negative	*Preussia* sp.	–	–	–	–	–	–	–	Not done	–	–	–
L27	Negative	–	–	–	–	–	–	–	–	Not done	–	–	–
L28	Negative	*Preussia* sp.	–	–	–	–	–	–	–	Not done	–	–	–
L29	Negative	–	–	–	–	–	–	–	–	Not done	–	–	–
L31	Negative	*Preussia* sp.	–	–	–	–	–	–	–	Not done	–	–	–
L32	Negative	–	–	–	–	–	–	–	–	Not done	–	–	–
L34	Negative	Uncultured eukaryote	–	–	–	–	–	–	–	Not done	–	–	–
L36	Negative	–	–	–	–	–	–	–	–	Not done	–	–	–
L37	Negative	–	–	–	–	–	–	–	–	Not done	–	–	–
L40	Negative	*Preussia* sp.	–	–	–	–	–	–	–	Not done	–	–	–
L44	Negative	*Sordaria fimicola*	–	–	–	–	–	–	–	Not done	–	–	–
L46	Negative	*Tilletiopsis washingtonensis*	–	–	–	–	–	–	–	Not done	–	–	–
L47	Negative	–	–	–	–	–	–	–	–	Not done	–	–	–
L49	Negative	–	–	–	–	–	–	–	–	Not done	–	–	–
J1	**Positive**	***C. ubiquitum***	None	Yes	Negative	Untypable	–	–	***C. ubiquitum***	Negative	–	–	–
J3	Negative	–	–	–	–	–	–	–	–	Negative	–	–	–
C2	Negative	–	–	–	–	–	–	–	–	Negative	–	–	–
V1	Negative	*Sordaria fimicola*	–	–	–	–	–	–	–	Negative	–	–	–
V2	Negative	–	–	–	–	–	–	–	–	Negative	–	–	–
V3	Negative	–	–	–	–	–	–	–	–	Negative	–	–	–
V4	Negative	*Ascitodus*	–	–	–	–	–	–	–	Negative	–	–	–
V5	Negative	–	–	–	–	–	–	–	–	Negative	–	–	–
V6	Negative	–	–	–	–	–	–	–	–	Negative	–	–	–
V9	Negative	–	–	–	–	–	–	–	–	Negative	–	–	–
V10	Negative	–	–	–	–	–	–	–	–	Negative	–	–	–
V11	Negative	–	–	–	–	–	–	–	–	Negative	–	–	–
V12	Negative	–	–	–	–	–	–	–	–	Negative	–	–	–
V13	Negative	–	–	–	–	–	–	–	–	Negative	–	–	–
V14	Negative	–	–	–	–	–	–	–	–	Negative	–	–	–
V16	Negative	–	–	–	–	–	–	–	–	Negative	–	–	–
V17	Negative	–	–	–	–	–	–	–	–	Negative	–	–	–
V18	Negative	*Preussia* sp.	–	–	–	–	–	–	–	Negative	–	–	–
V21	Negative	–	–	–	–	–	–	–	–	Negative	–	–	–
V22	Negative	*Eparis*	–	–	–	–	–	–	–	Negative	–	–	–
JAMNP1	Negative	–	–	–	–	–	–	–	–	Negative	–	–	–
JAMNP2	Negative	–	–	–	–	–	–	–	–	Negative	–	–	–
JAMNP3	**Positive**	***C. scrofarum***	438InsTT	Yes	–	–	–	–	***C. scrofarum***	Negative	–	–	–
JAMNP4	Negative	–	–	–	–	–	–	–	–	Negative	–	–	–
JAMNP5	**Positive**	***C. scrofarum***	None	No	–	–	–	–	***C. scrofarum***	Negative	–	–	–
JAMNP6	Negative	–	–	–	–	–	–	–	–	Negative	–	–	–
JAMNP7	**Positive**	***C. scrofarum***	None	Yes	–	–	–	–	***C. scrofarum***	Negative	–	–	–
JAMNP8	Negative	–	–	–	–	–	–	–	–	Negative	–	–	–
JAMNP9	**Positive**	***C. scrofarum***	None	Yes	–	–	–	–	***C. scrofarum***	Negative	–	–	–
JAMNP10	Negative	–	–	–	–	–	–	–	–	Negative	–	–	–
JAMNP11	Negative	–	–	–	–	–	–	–	–	Negative	–	–	–
JAMNP12	Negative	–	–	–	–	–	–	–	–	Negative	–	–	–
JAMNP13	Negative	–	–	–	–	–	–	–	–	Negative	–	–	–
JAMNP14	**Positive**	***C. scrofarum***	None	Yes	–	–	–	–	***C. scrofarum***	Negative	–	–	–
JAMNP15	**Positive**	***C. scrofarum***	None	Yes	–	–	–	–	***C. scrofarum***	Negative	–	–	–
JAMNP16	Negative	–	–	–	–	–	–	–	–	Negative	–	–	–
JAMNP17	Negative	–	–	–	–	–	–	–	–	Negative	–	–	–
JAMNP18	Negative	–	–	–	–	–	–	–	–	Negative	–	–	–
JAMNP19	Negative	–	–	–	–	–	–	–	–	Negative	–	–	–
JAMNP20	Negative	–	–	–	–	–	–	–	–	Negative	–	–	–
JAMNP21	Negative	–	–	–	–	–	–	–	–	Negative	–	–	–
JAMNP22	Negative	–	–	–	–	–	–	–	–	Negative	–	–	–
JAMNP23	Negative	–	–	–	–	–	–	–	–	Negative	–	–	–
JAMNP24	Negative	–	–	–	–	–	–	–	–	Negative	–	–	–
JAMNP25	Negative	–	–	–	–	–	–	–	–	Negative	–	–	–
JAMNP26	**Positive**	***C. scrofarum***	None	Yes	–	–	–	–	***C. scrofarum***	Negative	–	–	–
JAMNP27	Negative	–	–	–	–	–	–	–	–	Negative	–	–	–
JAMNP28	Negative	–	–	–	–	–	–	–	–	Negative	–	–	–
VAMNP8	Negative	–	–	–	–	–	–	–	–	Negative	–	–	–
VAMNP9	Negative	–	–	–	–	–	–	–	–	Negative	–	–	–
VAMNP10	Negative	–	–	–	–	–	–	–	–	Negative	–	–	–
VAMNP11	Negative	–	–	–	–	–	–	–	–	Negative	–	–	–
VAMNP12	Negative	–	–	–	–	–	–	–	–	Negative	–	–	–
VAMNP13	Negative	–	–	–	–	–	–	–	–	Negative	–	–	–
VAMNP14	Negative	–	–	–	–	–	–	–	–	Negative	–	–	–
VAMNP15	Negative	–	–	–	–	–	–	–	–	Negative	–	–	–
VAMNP16	Negative	–	–	–	–	–	–	–	–	Negative	–	–	–
VAMNP17	Negative	–	–	–	–	–	–	–	–	Negative	–	–	–
VAMNP18	Negative	–	–	–	–	–	–	–	–	Negative	–	–	–
VAMNP19	Negative	–	–	–	–	–	–	–	–	Negative	–	–	–
VAMNP20	Negative	–	–	–	–	–	–	–	–	Negative	–	–	–
VAMNP21	Negative	–	–	–	–	–	–	–	–	Negative	–	–	–
VAMNP22	Negative	–	–	–	–	–	–	–	–	Negative	–	–	–
VAMNP23	Negative	–	–	–	–	–	–	–	–	Negative	–	–	–
VAMNP24	Negative	–	–	–	–	–	–	–	–	Negative	–	–	–
VAMNP25	Negative	–	–	–	–	–	–	–	–	Negative	–	–	–
VAMNP26	Negative	–	–	–	–	–	–	–	–	Negative	–	–	–
VAMNP27	Negative	–	–	–	–	–	–	–	–	Negative	–	–	–
VAMNP28	Negative	–	–	–	–	–	–	–	–	Negative	–	–	–
VAMNP29	Negative	–	–	–	–	–	–	–	–	Negative	–	–	–
VAMNP30	Negative	–	–	–	–	–	–	–	–	Negative	–	–	–
VAMNP31	Negative	–	–	–	–	–	–	–	–	Negative	–	–	–
VAMNP32	Negative	–	–	–	–	–	–	–	–	Negative	–	–	–
VAMNP33	Negative	–	–	–	–	–	–	–	–	Negative	–	–	–
RMNP1	Negative	–	–	–	–	–	–	–	–	Not done	–	–	–
LMNP1	Negative	–	–	–	–	–	–	–	–	Not done	–	–	–
RMNP2	Negative	–	–	–	–	–	–	–	–	Not done	–	–	–
LMNP2	Negative	–	–	–	–	–	–	–	–	Not done	–	–	–
LMNP3	**Positive**	***C. canis***	None	Yes	Untypable	–	–	–	***C. canis***	Not done	–	–	–
LMNP4	Negative	–	–	–	–	–	–	–	–	Not done	–	–	–
LMNP5	Negative	–	–	–	–	–	–	–	–	Not done	–	–	–
LMNP6	Negative	–	–	–	–	–	–	–	–	Not done	–	–	–
RMNP3	Negative	–	–	–	–	–	–	–	–	Not done	–	–	–

RMNP4	MNP	Red fox	*Vulpes vulpes*	Negative	–	–	–	–	–	–	–	–	–	–	–	–	–	–	–	–	–	–	Negative	–	–	–	–	–	–	–	–	Not done	–	–	–
VMNP1	MNP	Red deer	*Cervus elaphus*	Negative	–	–	–	–	–	–	–	–	–	–	–	–	–	–	–	–	–	–	Negative	–	–	–	–	–	–	–	–	Negative	–	–	–
VMNP2	MNP	Red deer	*Cervus elaphus*	Negative	–	–	–	–	–	–	–	–	–	–	–	–	–	–	–	–	–	–	Negative	–	–	–	–	–	–	–	–	Negative	–	–	–
VMNP3	MNP	Red deer	*Cervus elaphus*	Negative	–	–	–	–	–	–	–	–	–	–	–	–	–	–	–	–	–	–	Negative	–	–	–	–	–	–	–	–	Negative	–	–	–
VMNP4	MNP	Red deer	*Cervus elaphus*	Negative	–	–	–	–	–	–	–	–	–	–	–	–	–	–	–	–	–	–	Negative	–	–	–	–	–	–	–	–	Negative	–	–	–
VMNP5	MNP	Red deer	*Cervus elaphus*	Negative	–	–	–	–	–	–	–	–	–	–	–	–	–	–	–	–	–	–	Negative	–	–	–	–	–	–	–	–	Negative	–	–	–
VMNP6	MNP	Red deer	*Cervus elaphus*	**Positive**	34.9	Negative	–	–	–	Not done	–	–	–	Not done	–	–	–	Not done	–	–	–	Unknown	Negative	–	–	–	–	–	–	–	–	Negative	–	–	–
VMNP7	MNP	Red deer	*Cervus elaphus*	Negative	–	–	–	–	–	–	–	–	–	–	–	–	–	–	–	–	–	–	Negative	–	–	–	–	–	–	–	–	Negative	–	–	–
VMNP8	MNP	Red deer	*Cervus elaphus*	Negative	–	–	–	–	–	–	–	–	–	–	–	–	–	–	–	–	–	–	Negative	–	–	–	–	–	–	–	–	Negative	–	–	–
VMNP9	MNP	Red deer	*Cervus elaphus*	Negative	–	–	–	–	–	–	–	–	–	–	–	–	–	–	–	–	–	–	Negative	–	–	–	–	–	–	–	–	Negative	–	–	–
CMNP1	MNP	Roe deer	*Capreolus capreolus*	Negative	–	–	–	–	–	–	–	–	–	–	–	–	–	–	–	–	–	–	Negative	–	–	–	–	–	–	–	–	Negative	–	–	–
CMNP2	MNP	Roe deer	*Capreolus capreolus*	**Positive**	38.0	Not done	–	–	–	Not done	–	–	–	Not done	–	–	–	Not done	–	–	–	Unknown	Negative	–	–	–	–	–	–	–	–	Negative	–	–	–
CMNP3	MNP	Roe deer	*Capreolus capreolus*	Negative	–	–	–	–	–	–	–	–	–	–	–	–	–	–	–	–	–	–	Negative	*Erica tetralix*	–	–	–	–	–	–	–	Negative	–	–	–
JMNP1	MNP	Wild boar	*Sus scrofa*	Negative	–	–	–	–	–	–	–	–	–	–	–	–	–	–	–	–	–	–	Negative	–	–	–	–	–	–	–	–	Negative	–	–	–
VAMNP1	MNP	Cattle	*Bos taurus*	Negative	–	–	–	–	–	–	–	–	–	–	–	–	–	–	–	–	–	–	Negative	–	–	–	–	–	–	–	–	Negative	–	–	–
VAMNP2	MNP	Cattle	*Bos taurus*	Negative	–	–	–	–	–	–	–	–	–	–	–	–	–	–	–	–	–	–	Negative	–	–	–	–	–	–	–	–	Negative	–	–	–
VAMNP3	MNP	Cattle	*Bos taurus*	Negative	–	–	–	–	–	–	–	–	–	–	–	–	–	–	–	–	–	–	Negative	–	–	–	–	–	–	–	–	Negative	–	–	–
OVMNP1	MNP	Sheep	*Ovis aries*	Negative	–	–	–	–	–	–	–	–	–	–	–	–	–	–	–	–	–	–	Negative	–	–	–	–	–	–	–	–	Negative	–	–	–
OVMNP2	MNP	Sheep	*Ovis aries*	Negative	–	–	–	–	–	–	–	–	–	–	–	–	–	–	–	–	–	–	Negative	–	–	–	–	–	–	–	–	Negative	–	–	–
OVMNP3	MNP	Sheep	*Ovis aries*	Negative	–	–	–	–	–	–	–	–	–	–	–	–	–	–	–	–	–	–	Negative	–	–	–	–	–	–	–	–	Negative	–	–	–
OVMNP4	MNP	Sheep	*Ovis aries*	Negative	–	–	–	–	–	–	–	–	–	–	–	–	–	–	–	–	–	–	Negative	–	–	–	–	–	–	–	–	Negative	–	–	–
LMNP9	MNP	Iberian wolf	*Canis lupus signatus*	Negative	–	–	–	–	–	–	–	–	–	–	–	–	–	–	–	–	–	–	Negative	–	–	–	–	–	–	–	–	Not done	–	–	–
LMNP12	MNP	Iberian wolf	*Canis lupus signatus*	Negative	–	–	–	–	–	–	–	–	–	–	–	–	–	–	–	–	–	–	Negative	–	–	–	–	–	–	–	–	Not done	–	–	–
LMNP13	MNP	Iberian wolf	*Canis lupus signatus*	Negative	–	–	–	–	–	–	–	–	–	–	–	–	–	–	–	–	–	–	Negative	–	–	–	–	–	–	–	–	Not done	–	–	–
LMNP16	MNP	Iberian wolf	*Canis lupus signatus*	Negative	–	–	–	–	–	–	–	–	–	–	–	–	–	–	–	–	–	–	Negative	–	–	–	–	–	–	–	–	Not done	–	–	–
LMNP17	MNP	Iberian wolf	*Canis lupus signatus*	Negative	–	–	–	–	–	–	–	–	–	–	–	–	–	–	–	–	–	–	Negative	–	–	–	–	–	–	–	–	Not done	–	–	–
LMNP18	MNP	Iberian wolf	*Canis lupus signatus*	Negative	–	–	–	–	–	–	–	–	–	–	–	–	–	–	–	–	–	–	Negative	–	–	–	–	–	–	–	–	Not done	–	–	–
LMNP19	MNP	Iberian wolf	*Canis lupus signatus*	**Positive**	32.7	Negative	–	–	–	Not done	–	–	–	Not done	–	–	–	Not done	–	–	–	Unknown	Negative	–	–	–	–	–	–	–	–	Not done	–	–	–
VMNP10	MNP	Red deer	*Cervus elaphus*	Negative	–	–	–	–	–	–	–	–	–	–	–	–	–	–	–	–	–	–	Negative	–	–	–	–	–	–	–	–	Negative	–	–	–
VMNP11	MNP	Red deer	*Cervus elaphus*	Negative	–	–	–	–	–	–	–	–	–	–	–	–	–	–	–	–	–	–	Negative	–	–	–	–	–	–	–	–	Negative	–	–	–
VMNP12	MNP	Red deer	*Cervus elaphus*	Negative	–	–	–	–	–	–	–	–	–	–	–	–	–	–	–	–	–	–	Negative	–	–	–	–	–	–	–	–	Negative	–	–	–
VMNP13	MNP	Red deer	*Cervus elaphus*	Negative	–	–	–	–	–	–	–	–	–	–	–	–	–	–	–	–	–	–	Negative	–	–	–	–	–	–	–	–	Negative	–	–	–
VMNP14	MNP	Red deer	*Cervus elaphus*	Negative	–	–	–	–	–	–	–	–	–	–	–	–	–	–	–	–	–	–	Negative	–	–	–	–	–	–	–	–	Negative	–	–	–
VMNP15	MNP	Red deer	*Cervus elaphus*	Negative	–	–	–	–	–	–	–	–	–	–	–	–	–	–	–	–	–	–	Negative	–	–	–	–	–	–	–	–	Negative	–	–	–
VMNP16	MNP	Red deer	*Cervus elaphus*	Negative	–	–	–	–	–	–	–	–	–	–	–	–	–	–	–	–	–	–	Negative	–	–	–	–	–	–	–	–	Negative			
VMNP18	MNP	Red deer	*Cervus elaphus*	Negative	–	–	–	–	–	–	–	–	–	–	–	–	–	–	–	–	–	–	Negative	–	–	–	–	–	–	–	–	Negative	–	–	–
VMNP19	MNP	Red deer	*Cervus elaphus*	Negative	–	–	–	–	–	–	–	–	–	–	–	–	–	–	–	–	–	–	Negative	–	–	–	–	–	–	–	–	Negative	–	–	–
VMNP20	MNP	Red deer	*Cervus elaphus*	Negative	–	–	–	–	–	–	–	–	–	–	–	–	–	–	–	–	–	–	Negative	–	–	–	–	–	–	–	–	Negative	–	–	–
VMNP21	MNP	Red deer	*Cervus elaphus*	Negative	–	–	–	–	–	–	–	–	–	–	–	–	–	–	–	–	–	–	Negative	–	–	–	–	–	–	–	–	Negative	–	–	–
VMNP22	MNP	Red deer	*Cervus elaphus*	Negative	–	–	–	–	–	–	–	–	–	–	–	–	–	–	–	–	–	–	Negative	–	–	–	–	–	–	–	–	Negative	–	–	–
VMNP23	MNP	Red deer	*Cervus elaphus*	Negative	–	–	–	–	–	–	–	–	–	–	–	–	–	–	–	–	–	–	Negative	–	–	–	–	–	–	–	–	Negative	–	–	–
VMNP24	MNP	Red deer	*Cervus elaphus*	Negative	–	–	–	–	–	–	–	–	–	–	–	–	–	–	–	–	–	–	Negative	–	–	–	–	–	–	–	–	Negative	–	–	–
CMNP4	MNP	Roe deer	*Capreolus capreolus*	**Positive**	36.9	Not done	–	–	–	Not done	–	–	–	Not done	–	–	–	Not done	–	–	–	Unknown	Negative	–	–	–	–	–	–	–	–	Negative	–	–	–
CMNP5	MNP	Roe deer	*Capreolus capreolus*	**Positive**	30.2	**Positive**	**A/AI**	A90C, A185R	Yes	Negative	–	–	–	Negative	–	–	–	Negative	–	–	–	**A/AI**	Negative	–	–	–	–	–	–	–	–	Negative	–	–	–
CMNP6	MNP	Roe deer	*Capreolus capreolus*	**Positive**	31.3	Negative	–	–	–	Negative	–	–	–	Negative	–	–	–	Negative	–	–	–	Unknown	Negative	–	–	–	–	–	–	–	–	Negative	–	–	–
CMNP7	MNP	Roe deer	*Capreolus capreolus*	Negative	–	–	–	–	–	–	–	–	–	–	–	–	–	–	–	–	–	–	Negative	–	–	–	–	–	–	–	–	Negative	–	–	–
VMNP25	MNP	Red deer	*Cervus elaphus*	**Positive**	36.9	Not done	–	–	–	Not done	–	–	–	Not done	–	–	–	Not done	–	–	–	Unknown	**Positive**	***C. ubiquitum***	None	Yes	Untypable	–	–	–	***C. ubiquitum***	Negative	–	–	–
VMNP26	MNP	Red deer	*Cervus elaphus*	Negative	–	–	–	–	–	–	–	–	–	–	–	–	–	–	–	–	–	–	Negative	Unspecific	–	–	–	–	–	–	–	Negative	–	–	–
VMNP27	MNP	Red deer	*Cervus elaphus*	Negative	–	–	–	–	–	–	–	–	–	–	–	–	–	–	–	–	–	–	Negative	Unspecific	–	–	–	–	–	–	–	Negative	–	–	–
VMNP28	MNP	Red deer	*Cervus elaphus*	Negative	–	–	–	–	–	–	–	–	–	–	–	–	–	–	–	–	–	–	Negative	*Colpodella tetrahymenae*	–	–	–	–	–	–	–	Negative	–	–	–
VMNP29	MNP	Red deer	*Cervus elaphus*	**Positive**	36.7	Not done	–	–	–	Not done	–	–	–	Not done	–	–	–	Not done	–	–	–	Unknown	Negative	–	–	–	–	–	–	–	–	Negative	–	–	–
VMNP30	MNP	Red deer	*Cervus elaphus*	Negative	–	–	–	–	–	–	–	–	–	–	–	–	–	–	–	–	–	–	Negative	–	–	–	–	–	–	–	–	Negative	–	–	–
VMNP31	MNP	Red deer	*Cervus elaphus*	Negative	–	–	–	–	–	–	–	–	–	–	–	–	–	–	–	–	–	–	Negative	–	–	–	–	–	–	–	–	Negative	–	–	–
VMNP32	MNP	Red deer	*Cervus elaphus*	Negative	–	–	–	–	–	–	–	–	–	–	–	–	–	–	–	–	–	–	Negative	–	–	–	–	–	–	–	–	Negative	–	–	–
VMNP33	MNP	Red deer	*Cervus elaphus*	Negative	–	–	–	–	–	–	–	–	–	–	–	–	–	–	–	–	–	–	**Positive**	***C. ryanae***	None	Yes	Untypable	–	–	–	***C. ryanae***	Negative	–	–	–
JMNP2	MNP	Wild boar	*Sus scrofa*	Negative	–	–	–	–	–	–	–	–	–	–	–	–	–	–	–	–	–	–	Negative	Uncultured eukaryote	–	–	–	–	–	–	–	Negative	–	–	–
JMNP3	MNP	Wild boar	*Sus scrofa*	Negative	–	–	–	–	–	–	–	–	–	–	–	–	–	–	–	–	–	–	Negative	–	–	–	–	–	–	–	–	Negative	–	–	–
JMNP4	MNP	Wild boar	*Sus scrofa*	**Positive**	31.7	Negative	–	–	–														Negative	–	–	–	–	–	–	–	–	Negative	–	–	–
JMNP5	MNP	Wild boar	*Sus scrofa*	**Positive**	32.9	Negative	–	–	–	Not done	–	–	–	Not done	–	–	–	Not done	–	–	–	Unknown	Negative	–	–	–	–	–	–	–	–	Negative	–	–	–
JMNP6	MNP	Wild boar	*Sus scrofa*	Negative	–	–	–	–	–	–	–	–	–	–	–	–	–	–	–	–	–	–	Negative	–	–	–	–	–	–	–	–	Negative	–	–	–
JMNP7	MNP	Wild boar	*Sus scrofa*	Negative	–	–	–	–	–	–	–	–	–	–	–	–	–	–	–	–	–	–	Negative	–	–	–	–	–	–	–	–	Negative	–	–	–
JMNP8	MNP	Wild boar	*Sus scrofa*	Negative	–	–	–	–	–	–	–	–	–	–	–	–	–	–	–	–	–	–	Negative	–	–	–	–	–	–	–	–	Negative	–	–	–
JMNP9	MNP	Wild boar	*Sus scrofa*	**Positive**	28.7	**Positive**	**E**	None	Yes	Negative	–	–	–	Negative	–	–	–	Negative	–	–	–	**E**	Negative	–	–	–	–	–	–	–	–	Negative	–	–	–
JMNP10	MNP	Wild boar	*Sus scrofa*	**Positive**	36.6	Not done	–	–	–	Not done	–	–	–	Not done	–	–	–	Not done	–	–	–	Unknown	Negative	–	–	–	–	–	–	–	–	Negative	–	–	–
JMNP11	MNP	Wild boar	*Sus scrofa*	**Positive**	34.7	Negative	–	–	–	Not done	–	–	–	Not done	–	–	–	Not done	–	–	–	Unknown	Negative	–	–	–	–	–	–	–	–	Negative	–	–	–
JMNP12	MNP	Wild boar	*Sus scrofa*	Negative	–	–	–	–	–	–	–	–	–	–	–	–	–	–	–	–	–	–	Negative	–	–	–	–	–	–	–	–	Negative	–	–	–
JMNP13	MNP	Wild boar	*Sus scrofa*	Negative	–	–	–	–	–	–	–	–	–	–	–	–	–	–	–	–	–	–	Negative	–	–	–	–	–	–	–	–	Negative	–	–	–
RMNP6	MNP	Red fox	*Vulpes vulpes*	Negative	–	–	–	–	–	–	–	–	–	–	–	–	–	–	–	–	–	–	Negative	–	–	–	–	–	–	–	–	Not done	–	–	–
RMNP7	MNP	Red fox	*Vulpes vulpes*	Negative	–	–	–	–	–	–	–	–	–	–	–	–	–	–	–	–	–	–	Negative	–	–	–	–	–	–	–	–	Not done	–	–	–
RMNP8	MNP	Red fox	*Vulpes vulpes*	Negative	–	–	–	–	–	–	–	–	–	–	–	–	–	–	–	–	–	–	Negative	–	–	–	–	–	–	–	–	Not done	–	–	–
RMNP9	MNP	Red fox	*Vulpes vulpes*	Negative	–	–	–	–	–	–	–	–	–	–	–	–	–	–	–	–	–	–	Negative	–	–	–	–	–	–	–	–	Not done	–	–	–
RMNP10	MNP	Red fox	*Vulpes vulpes*	Negative	–	–	–	–	–	–	–	–	–	–	–	–	–	–	–	–	–	–	Negative	–	–	–	–	–	–	–	–	Not done	–	–	–
RMNP11	MNP	Red fox	*Vulpes vulpes*	Negative	–	–	–	–	–	–	–	–	–	–	–	–	–	–	–	–	–	–	Negative	Unspecific	–	–	–	–	–	–	–	Not done	–	–	–
RMNP12	MNP	Red fox	*Vulpes vulpes*	Negative	–	–	–	–	–	–	–	–	–	–	–	–	–	–	–	–	–	–	Negative	–	–	–	–	–	–	–	–	Not done	–	–	–
RMNP13	MNP	Red fox	*Vulpes vulpes*	Negative	–	–	–	–	–	–	–	–	–	–	–	–	–	–	–	–	–	–	Negative	–	–	–	–	–	–	–	–	Not done	–	–	–
RMNP14	MNP	Red fox	*Vulpes vulpes*	**Positive**	36.1	Not done	–	–	–	Not done	–	–	–	Not done	–	–	–	Not done	–	–	–	Unknown	**Positive**	***C. canis***	686InsTG, T739A	Yes	Untypable	–	–	–	***C. canis***	Not done	–	–	–
RMNP15	MNP	Red fox	*Vulpes vulpes*	Negative	–	–	–	–	–	–	–	–	–	–	–	–	–	–	–	–	–	–	Negative	–	–	–	–	–	–	–	–	Not done	–	–	–
OVMNP5	MNP	Sheep	*Ovis aries*	Negative	–	–	–	–	–	–	–	–	–	–	–	–	–	–	–	–	–	–	Negative	–	–	–	–	–	–	–	–	Negative	–	–	–
OVMNP6	MNP	Sheep	*Ovis aries*	Negative	–	–	–	–	–	–	–	–	–	–	–	–	–	–	–	–	–	–	Negative	–	–	–	–	–	–	–	–	Negative	–	–	–
OVMNP7	MNP	Sheep	*Ovis aries*	**Positive**	34	Negative	–	–	–	Not done	–	–	–	Not done	–	–	–	Not done	–	–	–	Unknown	Negative	–	–	–	–	–	–	–	–	Negative	–	–	–
OVMNP8	MNP	Sheep	*Ovis aries*	Negative	–	–	–	–	–	–	–	–	–	–	–	–	–	–	–	–	–	–	Negative	–	–	–	–	–	–	–	–	Negative	–	–	–
OVMNP9	MNP	Sheep	*Ovis aries*	Negative	–	–	–	–	–	–	–	–	–	–	–	–	–	–	–	–	–	–	Negative	–	–	–	–	–	–	–	–	Negative	–	–	–
OVMNP10	MNP	Sheep	*Ovis aries*	Negative	–	–	–	–	–	–	–	–	–	–	–	–	–	–	–	–	–	–	Negative	–	–	–	–	–	–	–	–	Negative	–	–	–
OVMNP11	MNP	Sheep	*Ovis aries*	**Positive**	33	**Positive**	**E**	None	Yes	Not done	–	–	–	Not done	–	–	–	Not done	–	–	–	**E**	Negative	–	–	–	–	–	–	–	–	Negative	–	–	–
OVMNP12	MNP	Sheep	*Ovis aries*	Negative	–	–	–	–	–	–	–	–	–	–	–	–	–	–	–	–	–	–	Negative	–	–	–	–	–	–	–	–	Negative	–	–	–
OVMNP13	MNP	Sheep	*Ovis aries*	Negative	–	–	–	–	–	–	–	–	–	–	–	–	–	–	–	–	–	–	Negative	–	–	–	–	–	–	–	–	Negative	–	–	–
OVMNP14	MNP	Sheep	*Ovis aries*	Negative	–	–	–	–	–	–	–	–	–	–	–	–	–	–	–	–	–	–	Negative	–	–	–	–	–	–	–	–	Negative	–	–	–
OVMNP15	MNP	Sheep	*Ovis aries*	Negative	–	–	–	–	–	–	–	–	–	–	–	–	–	–	–	–	–	–	Negative	–	–	–	–	–	–	–	–	Negative	–	–	–
VAMNP4	MNP	Cattle	*Bos taurus*	Negative	–	–	–	–	–	–	–	–	–	–	–	–	–	–	–	–	–	–	Negative	–	–	–	–	–	–	–	–	Negative	–	–	–
VAMNP5	MNP	Cattle	*Bos taurus*	Negative	–	–	–	–	–	–	–	–	–	–	–	–	–	–	–	–	–	–	Negative	–	–	–	–	–	–	–	–	Negative	–	–	–
VAMNP6	MNP	Cattle	*Bos taurus*	Negative	–	–	–	–	–	–	–	–	–	–	–	–	–	–	–	–	–	–	Negative	–	–	–	–	–	–	–	–	Negative	–	–	–
VAMNP7	MNP	Cattle	*Bos taurus*	Negative	–	–	–	–	–	–	–	–	–	–	–	–	–	–	–	–	–	–	Negative	–	–	–	–	–	–	–	–	Negative	–	–	–
RMNP16	MNP	Red fox	*Vulpes vulpes*	Negative	–	–	–	–	–	–	–	–	–	–	–	–	–	–	–	–	–	–	Negative	–	–	–	–	–	–	–	–	Not done	–	–	–
RMNP17	MNP	Red fox	*Vulpes vulpes*	Negative	–	–	–	–	–	–	–	–	–	–	–	–	–	–	–	–	–	–	Negative	–	–	–	–	–	–	–	–	Not done	–	–	–
RMNP18	MNP	Red fox	*Vulpes vulpes*	**Positive**	34.6	Negative	–	–	–	Not done	–	–	–	Not done	–	–	–	Not done	–	–	–	Unknown	Negative	–	–	–	–	–	–	–	–	Not done	–	–	–
RMNP19	MNP	Red fox	*Vulpes vulpes*	Negative	–	–	–	–	–	–	–	–	–	–	–	–	–	–	–	–	–	–	**Positive**	***C. canis***	686InsTG, T739A	Yes	Untypable	–	–	–	***C. canis***	Not done	–	–	–
RMNP20	MNP	Red fox	*Vulpes vulpes*	**Positive**	33.9	Negative	–	–	–	Not done	–	–	–	Not done	–	–	–	Not done	–	–	–	Unknown	Negative	–	–	–	–	–	–	–	–	Not done	–	–	–
RMNP21	MNP	Red fox	*Vulpes vulpes*	Negative	–	–	–	–	–	–	–	–	–	–	–	–	–	–	–	–	–	–	Negative	–	–	–	–	–	–	–	–	Not done	–	–	–
LMNP21	MNP	Iberian wolf	*Canis lupus signatus*	Negative	–	–	–	–	–	–	–	–	–	–	–	–	–	–	–	–	–	–	Negative	–	–	–	–	–	–	–	–	Not done	–	–	–
LMNP23	MNP	Iberian wolf	*Canis lupus signatus*	**Positive**	25.2	Negative	–	–	–	**Positive**	**D**	T429C, G441A	Yes	**Positive**	**D**	A159R, A201G, C207Y	Yes	Negative	–	–	–	**D**	Negative	–	–	–	–	–	–	–	–	Not done	–	–	–
LMNP25	MNP	Iberian wolf	*Canis lupus signatus*	**Positive**	29.9	Negative	–	–	–	Negative	–	–	–	Negative	–	–	–	Negative	–	–	–	Unknown	Negative	–	–	–	–	–	–	–	–	Not done	–	–	–
OVMNP16	MNP	Sheep	*Ovis aries*	Negative	–	–	–	–	–	–	–	–	–	–	–	–	–	–	–	–	–	–	Negative	–	–	–	–	–	–	–	–	Negative	–	–	–
OVMNP17	MNP	Sheep	*Ovis aries*	Negative	–	–	–	–	–	–	–	–	–	–	–	–	–	–	–	–	–	–	Negative	–	–	–	–	–	–	–	–	Negative	–	–	–
OVMNP18	MNP	Sheep	*Ovis aries*	Negative	–	–	–	–	–	–	–	–	–	–	–	–	–	–	–	–	–	–	Negative	–	–	–	–	–	–	–	–	Negative	–	–	–
OVMNP19	MNP	Sheep	*Ovis aries*	Negative	–	–	–	–	–	–	–	–	–	–	–	–	–	–	–	–	–	–	Negative	–	–	–	–	–	–	–	–	Negative	–	–	–
OVMNP20	MNP	Sheep	*Ovis aries*	Negative	–	–	–	–	–	–	–	–	–	–	–	–	–	–	–	–	–	–	Negative	–	–	–	–	–	–	–	–	Negative	–	–	–
OVMNP21	MNP	Sheep	*Ovis aries*	**Positive**	33.7	Negative	–	–	–	Not done	–	–	–	Not done	–	–	–	Not done	–	–	–	Unknown	Negative	–	–	–	–	–	–	–	–	Negative	–	–	–
OVMNP22	MNP	Sheep	*Ovis aries*	**Positive**	34.8	Negative	–	–	–	Not done	–	–	–	Not done	–	–	–	Not done	–	–	–	Unknown	Negative	–	–	–	–	–	–	–	–	Negative	–	–	–
OVMNP23	MNP	Sheep	*Ovis aries*	Negative	–	–	–	–	–	–	–	–	–	–	–	–	–	–	–	–	–	–	Negative	–	–	–	–	–	–	–	–	Negative	–	–	–
OVMNP24	MNP	Sheep	*Ovis aries*	Negative	–	–	–	–	–	–	–	–	–	–	–	–	–	–	–	–	–	–	Negative	–	–	–	–	–	–	–	–	Negative	–	–	–
RMT1	CPW	Red fox	*Vulpes vulpes*	**Positive**	36.4	Not done	–	–	–	Not done	–	–	–	Not done	–	–	–	Not done	–	–	–	Unknown	Negative	–	–	–	–	–	–	–	–	Not done	–	–	–

RMT2	CPW	Red fox	*Vulpes vulpes*	Negative	–	–	–	–	–	–	–	–	–	–	–	–	–	–	–	–	–	–	Negative	–	–	–	–	–	–	–	–	Not done	–	–	–
RMT3	CPW	Red fox	*Vulpes vulpes*	**Positive**	33.7	Negative	–	–	–	Not done	–	–	–	Not done	–	–	–	Not done	–	–	–	Unknown	Negative	–	–	–	–	–	–	–	–	Not done	–	–	–
LMT1	CPW	Red fox	*Vulpes vulpes*	Negative	–	–	–	–	–	–	–	–	–	–	–	–	–	–	–	–	–	–	Negative	–	–	–	–	–	–	–	–	Not done	–	–	–
RMT4	CPW	Red fox	*Vulpes vulpes*	**Positive**	37.5	Not done	–	–	–	Not done	–	–	–	Not done	–	–	–	Not done	–	–	–	Unknown	Negative	*Tilletiopsis pallescens*	–	–	–	–	–	–	–	Not done	–	–	–
RMT5	CPW	Red fox	*Vulpes vulpes*	**Positive**	35.5	Not done	–	–	–	Not done	–	–	–	Not done	–	–	–	Not done	–	–	–	Unknown	Negative	–	–	–	–	–	–	–	–	Not done	–	–	–
RMT6	CPW	Red fox	*Vulpes vulpes*	Negative	–	–	–	–	–	–	–	–	–	–	–	–	–	–	–	–	–	–	Negative	Unspecific	–	–	–	–	–	–	–	Not done	–	–	–
RMT7	CPW	Red fox	*Vulpes vulpes*	Negative	–	–	–	–	–	–	–	–	–	–	–	–	–	–	–	–	–	–	Negative	–	–	–	–	–	–	–	–	Not done	–	–	–
RMT8	CPW	Red fox	*Vulpes vulpes*	**Positive**	34.2	Negative	–	–	–	Not done	–	–	–	Not done	–	–	–	Not done	–	–	–	Unknown	Negative	–	–	–	–	–	–	–	–	Not done	–	–	–
RMT9	CPW	Red fox	*Vulpes vulpes*	**Positive**	32.7	Negative	–	–	–	Not done	–	–	–	Not done	–	–	–	Not done	–	–	–	Unknown	Negative	–	–	–	–	–	–	–	–	Not done	–	–	–
RMT10	CPW	Red fox	*Vulpes vulpes*	Negative	–	–	–	–	–	–	–	–	–	–	–	–	–	–	–	–	–	–	Negative	–	–	–	–	–	–	–	–	Not done	–	–	–
RMT11	CPW	Red fox	*Vulpes vulpes*	Negative	–	–	–	–	–	–	–	–	–	–	–	–	–	–	–	–	–	–	Negative	–	–	–	–	–	–	–	–	Not done	–	–	–
RMT12	CPW	Red fox	*Vulpes vulpes*	Negative	–	–	–	–	–	–	–	–	–	–	–	–	–	–	–	–	–	–	Negative	–	–	–	–	–	–	–	–	Not done	–	–	–
RMT13	CPW	Red fox	*Vulpes vulpes*	Negative	–	–	–	–	–	–	–	–	–	–	–	–	–	–	–	–	–	–	Negative	–	–	–	–	–	–	–	–	Not done	–	–	–
RFA1	CPW	Red fox	*Vulpes vulpes*	Negative	–	–	–	–	–	–	–	–	–	–	–	–	–	–	–	–	–	–	Negative	Unspecific	–	–	–	–	–	–	–	Not done	–	–	–
RFA2	CPW	Red fox	*Vulpes vulpes*	Negative	–	–	–	–	–	–	–	–	–	–	–	–	–	–	–	–	–	–	Negative	–	–	–	–	–	–	–	–	Not done	–	–	–
RFA3	CPW	Red fox	*Vulpes vulpes*	Negative	–	–	–	–	–	–	–	–	–	–	–	–	–	–	–	–	–	–	Negative	*Sordaria macrospora*	–	–	–	–	–	–	–	Not done	–	–	–
RFA4	CPW	Red fox	*Vulpes vulpes*	Negative	–	–	–	–	–	–	–	–	–	–	–	–	–	–	–	–	–	–	Negative	–	–	–	–	–	–	–	–	Not done	–	–	–
RFA5	CPW	Red fox	*Vulpes vulpes*	Negative	–	–	–	–	–	–	–	–	–	–	–	–	–	–	–	–	–	–	Negative	Unspecific	–	–	–	–	–	–	–	Not done	–	–	–
RFA6	CPW	Red fox	*Vulpes vulpes*	Negative	–	–	–	–	–	–	–	–	–	–	–	–	–	–	–	–	–	–	Negative	–	–	–	–	–	–	–	–	Not done	–	–	–
RFA7	CPW	Red fox	*Vulpes vulpes*	Negative	–	–	–	–	–	–	–	–	–	–	–	–	–	–	–	–	–	–	Negative	–	–	–	–	–	–	–	–	Not done	–	–	–
RFA9	CPW	Red fox	*Vulpes vulpes*	Negative	–	–	–	–	–	–	–	–	–	–	–	–	–	–	–	–	–	–	Negative	–	–	–	–	–	–	–	–	Not done	–	–	–
RFA10	CPW	Red fox	*Vulpes vulpes*	Negative	–	–	–	–	–	–	–	–	–	–	–	–	–	–	–	–	–	–	Negative	*Amphichorda guana*	–	–	–	–	–	–	–	Not done	–	–	–
RFA11	CPW	Red fox	*Vulpes vulpes*	Negative	–	–	–	–	–	–	–	–	–	–	–	–	–	–	–	–	–	–	Negative	Unspecific	–	–	–	–	–	–	–	Not done	–	–	–
FFA1	CPW	Stone marten	*Martes foina*	Negative	–	–	–	–	–	–	–	–	–	–	–	–	–	–	–	–	–	–	Negative	–	–	–	–	–	–	–	–	Not done	–	–	–
FFA2	CPW	Stone marten	*Martes foina*	Negative	–	–	–	–	–	–	–	–	–	–	–	–	–	–	–	–	–	–	Negative	–	–	–	–	–	–	–	–	Not done	–	–	–
CAMT1	CPW	Goat	*Capra hircus*	Negative	–	–	–	–	–	–	–	–	–	–	–	–	–	–	–	–	–	–	Negative	–	–	–	–	–	–	–	–	Negative	–	–	–
CAMT2	CPW	Goat	*Capra hircus*	Negative	–	–	–	–	–	–	–	–	–	–	–	–	–	–	–	–	–	–	Negative	–	–	–	–	–	–	–	–	Negative	–	–	–
CAMT3	CPW	Goat	*Capra hircus*	Negative	–	–	–	–	–	–	–	–	–	–	–	–	–	–	–	–	–	–	Negative	–	–	–	–	–	–	–	–	Negative	–	–	–
CAMT4	CPW	Goat	*Capra hircus*	Negative	–	–	–	–	–	–	–	–	–	–	–	–	–	–	–	–	–	–	Negative	Unspecific	–	–	–	–	–	–	–	Negative	–	–	–
CAMT5	CPW	Goat	*Capra hircus*	Negative	–	–	–	–	–	–	–	–	–	–	–	–	–	–	–	–	–	–	Negative	–	–	–	–	–	–	–	–	Negative	–	–	–
CAMT6	CPW	Goat	*Capra hircus*	Negative	–	–	–	–	–	–	–	–	–	–	–	–	–	–	–	–	–	–	Negative	–	–	–	–	–	–	–	–	Negative	–	–	–
VAMT1	CPW	Cattle	*Bos taurus*	Negative	–	–	–	–	–	–	–	–	–	–	–	–	–	–	–	–	–	–	Negative	–	–	–	–	–	–	–	–	Negative	–	–	–
CAFA1	CPW	Goat	*Capra hircus*	Negative	–	–	–	–	–	–	–	–	–	–	–	–	–	–	–	–	–	–	Negative	–	–	–	–	–	–	–	–	Negative	–	–	–
CAFA2	CPW	Goat	*Capra hircus*	Negative	–	–	–	–	–	–	–	–	–	–	–	–	–	–	–	–	–	–	Negative	–	–	–	–	–	–	–	–	Negative	–	–	–
CAFA3	CPW	Goat	*Capra hircus*	Negative	–	–	–	–	–	–	–	–	–	–	–	–	–	–	–	–	–	–	Negative	–	–	–	–	–	–	–	–	Negative	–	–	–
CAFA4	CPW	Goat	*Capra hircus*	Negative	–	–	–	–	–	–	–	–	–	–	–	–	–	–	–	–	–	–	Negative	–	–	–	–	–	–	–	–	Negative	–	–	–
VAFA1	CPW	Cattle	*Bos taurus*	Negative	–	–	–	–	–	–	–	–	–	–	–	–	–	–	–	–	–	–	Negative	–	–	–	–	–	–	–	–	Negative	–	–	–
VAFA2	CPW	Cattle	*Bos taurus*	Negative	–	–	–	–	–	–	–	–	–	–	–	–	–	–	–	–	–	–	Negative	–	–	–	–	–	–	–	–	Negative	–	–	–
RMT14	CPW	Red fox	*Vulpes vulpes*	**Positive**	36.7	Not done	–	–	–	Not done	–	–	–	Not done	–	–	–	Not done	–	–	–	Unknown	Negative	–	–	–	–	–	–	–	–	Not done	–	–	–
RMT15	CPW	Red fox	*Vulpes vulpes*	Negative	–	–	–	–	–	–	–	–	–	–	–	–	–	–	–	–	–	–	Negative	–	–	–	–	–	–	–	–	Not done	–	–	–
RMT16	CPW	Red fox	*Vulpes vulpes*	Negative	–	–	–	–	–	–	–	–	–	–	–	–	–	–	–	–	–	–	Negative	–	–	–	–	–	–	–	–	Not done	–	–	–
RFA12	CPW	Red fox	*Vulpes vulpes*	**Positive**	36.1	Not done	–	–	–	Not done	–	–	–	Not done	–	–	–	Not done	–	–	–	Unknown	Negative	–	–	–	–	–	–	–	–	Not done	–	–	–
RFA13	CPW	Red fox	*Vulpes vulpes*	Negative	–	–	–	–	–	–	–	–	–	–	–	–	–	–	–	–	–	–	Negative	–	–	–	–	–	–	–	–	Not done	–	–	–
RFA14	CPW	Red fox	*Vulpes vulpes*	Negative	–	–	–	–	–	–	–	–	–	–	–	–	–	–	–	–	–	–	Negative	–	–	–	–	–	–	–	–	Not done	–	–	–
CAFA5	CPW	Goat	*Capra hircus*	Negative	–	–	–	–	–	–	–	–	–	–	–	–	–	–	–	–	–	–	Negative	–	–	–	–	–	–	–	–	Negative	–	–	–
CAFA6	CPW	Goat	*Capra hircus*	Negative	–	–	–	–	–	–	–	–	–	–	–	–	–	–	–	–	–	–	Negative	Unspecific	–	–	–	–	–	–	–	Negative	–	–	–
CAFA7	CPW	Goat	*Capra hircus*	Negative	–	–	–	–	–	–	–	–	–	–	–	–	–	–	–	–	–	–	Negative	–	–	–	–	–	–	–	–	Negative	–	–	–
JFA1	CPW	Wild boar	*Sus scrofa*	Negative	–	–	–	–	–	–	–	–	–	–	–	–	–	–	–	–	–	–	Negative	*Gnomoniopsis smithogilvyi*	–	–	–	–	–	–	–	Negative	–	–	–
VAFA3	CPW	Cattle	*Bos taurus*	Negative	–	–	–	–	–	–	–	–	–	–	–	–	–	–	–	–	–	–	Negative	–	–	–	–	–	–	–	–	Negative	–	–	–
VAFA4	CPW	Cattle	*Bos taurus*	Negative	–	–	–	–	–	–	–	–	–	–	–	–	–	–	–	–	–	–	Negative	–	–	–	–	–	–	–	–	Negative	–	–	–
VAFA5	CPW	Cattle	*Bos taurus*	Negative	–	–	–	–	–	–	–	–	–	–	–	–	–	–	–	–	–	–	Negative	–	–	–	–	–	–	–	–	Negative	–	–	–
VAFA6	CPW	Cattle	*Bos taurus*	Negative	–	–	–	–	–	–	–	–	–	–	–	–	–	–	–	–	–	–	Negative	–	–	–	–	–	–	–	–	Negative	–	–	–
RMT17	CPW	Red fox	*Vulpes vulpes*	Negative	–	–	–	–	–	–	–	–	–	–	–	–	–	–	–	–	–	–	Negative	–	–	–	–	–	–	–	–	Not done	–	–	–
VAMT2	CPW	Cattle	*Bos taurus*	Negative	–	–	–	–	–	–	–	–	–	–	–	–	–	–	–	–	–	–	Negative	–	–	–	–	–	–	–	–	Negative	–	–	–
JMT1	CPW	Wild boar	*Sus scrofa*	**Positive**	35.8	Not done	–	–	–	Not done	–	–	–	Not done	–	–	–	Not done	–	–	–	Unknown	Negative	–	–	–	–	–	–	–	–	Negative	–	–	–
FMT1	CPW	Stone marten	*Martes foina*	**Positive**	37.4	Not done	–	–	–	Not done	–	–	–	Not done	–	–	–	Not done	–	–	–	Unknown	Negative	–	–	–	–	–	–	–	–	Not done	–	–	–
VAMT3	CPW	Cattle	*Bos taurus*	Negative	–	–	–	–	–	–	–	–	–	–	–	–	–	–	–	–	–	–	Negative	–	–	–	–	–	–	–	–	Negative	–	–	–
VAMT4	CPW	Cattle	*Bos taurus*	**Positive**	36.2	Not done	–	–	–	Not done	–	–	–	Not done	–	–	–	Not done	–	–	–	Unknown	Negative	–	–	–	–	–	–	–	–	Negative	–	–	–
CAMT7	CPW	Goat	*Capra hircus*	Negative	–	–	–	–	–	–	–	–	–	–	–	–	–	–	–	–	–	–	Negative	–	–	–	–	–	–	–	–	Negative	–	–	–
OVMT1	CPW	Sheep	*Ovis aries*	**Positive**	35.5	Not done	–	–	–	Not done	–	–	–	Not done	–	–	–	Not done	–	–	–	Unknown	Negative	–	–	–	–	–	–	–	–	Negative	–	–	–
VAMT5	CPW	Cattle	*Bos taurus*	**Positive**	33.8	**Positive**	**E**	None	Yes	Not done	–	–	–	Not done	–	–	–	Not done	–	–	–	**E**	Negative	–	–	–	–	–	–	–	–	Negative	–	–	–
OVMT2	CPW	Sheep	*Ovis aries*	Negative	–	–	–	–	–	–	–	–	–	–	–	–	–	–	–	–	–	–	Negative	–	–	–	–	–	–	–	–	Negative	–	–	–
VAMT6	CPW	Cattle	*Bos taurus*	Negative	–	–	–	–	–	–	–	–	–	–	–	–	–	–	–	–	–	–	Negative	–	–	–	–	–	–	–	–	Negative	–	–	–
FMT2	CPW	Stone marten	*Martes foina*	Negative	–	–	–	–	–	–	–	–	–	–	–	–	–	–	–	–	–	–	Negative	–	–	–	–	–	–	–	–	Not done	–	–	–
JMT2	CPW	Wild boar	*Sus scrofa*	Negative	–	–	–	–	–	–	–	–	–	–	–	–	–	–	–	–	–	–	Negative	–	–	–	–	–	–	–	–	Negative	–	–	–
OVMT3	CPW	Sheep	*Ovis aries*	Negative	–	–	–	–	–	–	–	–	–	–	–	–	–	–	–	–	–	–	Negative	–	–	–	–	–	–	–	–	Negative	–	–	–
FMT3	CPW	Stone marten	*Martes foina*	Negative	–	–	–	–	–	–	–	–	–	–	–	–	–	–	–	–	–	–	Negative	–	–	–	–	–	–	–	–	Not done	–	–	–
RMT18	CPW	Red fox	*Vulpes vulpes*	Negative	–	–	–	–	–	–	–	–	–	–	–	–	–	–	–	–	–	–	Negative	–	–	–	–	–	–	–	–	Not done	–	–	–
FMT4	CPW	Stone marten	*Martes foina*	Negative	–	–	–	–	–	–	–	–	–	–	–	–	–	–	–	–	–	–	Negative	–	–	–	–	–	–	–	–	Not done	–	–	–
CAMT8	CPW	Goat	*Capra hircus*	Negative	–	–	–	–	–	–	–	–	–	–	–	–	–	–	–	–	–	–	Negative	–	–	–	–	–	–	–	–	Negative	–	–	–
OVMT4	CPW	Sheep	*Ovis aries*	Negative	–	–	–	–	–	–	–	–	–	–	–	–	–	–	–	–	–	–	Negative	–	–	–	–	–	–	–	–	Negative	–	–	–
CAMT9	CPW	Goat	*Capra hircus*	Negative	–	–	–	–	–	–	–	–	–	–	–	–	–	–	–	–	–	–	Negative	–	–	–	–	–	–	–	–	Negative	–	–	–
OVMT5	CPW	Sheep	*Ovis aries*	Negative	–	–	–	–	–	–	–	–	–	–	–	–	–	–	–	–	–	–	Negative	–	–	–	–	–	–	–	–	Negative	–	–	–
RFA15	CPW	Red fox	*Vulpes vulpes*	**Positive**	34.6	Negative	–	–	–	Not done	–	–	–	Not done	–	–	–	Not done	–	–	–	Unknown	Negative	–	–	–	–	–	–	–	–	Not done	–	–	–
RMT19	CPW	Red fox	*Vulpes vulpes*	**Positive**	34.8	Negative	–	–	–	Not done	–	–	–	Not done	–	–	–	Not done	–	–	–	Unknown	Negative	Uncultured eukaryote	–	–	–	–	–	–	–	Not done	–	–	–
RMT20	CPW	Red fox	*Vulpes vulpes*	Negative	–	–	–	–	–	–	–	–	–	–	–	–	–	–	–	–	–	–	Negative	*Neospora caninum*	–	–	–	–	–	–	–	Not done	–	–	–
RMT21	CPW	Red fox	*Vulpes vulpes*	Negative	–	–	–	–	–	–	–	–	–	–	–	–	–	–	–	–	–	–	Negative	–	–	–	–	–	–	–	–	Not done	–	–	–
FFA3	CPW	Stone marten	*Martes foina*	Negative	–	–	–	–	–	–	–	–	–	–	–	–	–	–	–	–	–	–	Negative	–	–	–	–	–	–	–	–	Not done	–	–	–
RMT22	CPW	Red fox	*Vulpes vulpes*	Negative	–	–	–	–	–	–	–	–	–	–	–	–	–	–	–	–	–	–	Negative	–	–	–	–	–	–	–	–	Not done	–	–	–
RFA16	CPW	Red fox	*Vulpes vulpes*	Negative	–	–	–	–	–	–	–	–	–	–	–	–	–	–	–	–	–	–	Negative	–	–	–	–	–	–	–	–	Not done	–	–	–
RMT23	CPW	Red fox	*Vulpes vulpes*	Negative	–	–	–	–	–	–	–	–	–	–	–	–	–	–	–	–	–	–	Negative	Uncultured eukaryote	–	–	–	–	–	–	–	Not done	–	–	–
RMT24	CPW	Red fox	*Vulpes vulpes*	Negative	–	–	–	–	–	–	–	–	–	–	–	–	–	–	–	–	–	–	Negative	–	–	–	–	–	–	–	–	Not done	–	–	–
CAMT10	CPW	Goat	*Capra hircus*	Negative	–	–	–	–	–	–	–	–	–	–	–	–	–	–	–	–	–	–	Negative	–	–	–	–	–	–	–	–	Negative	–	–	–
CAMT11	CPW	Goat	*Capra hircus*	Negative	–	–	–	–	–	–	–	–	–	–	–	–	–	–	–	–	–	–	Negative	–	–	–	–	–	–	–	–	Negative	–	–	–
FMT5	CPW	Stone marten	*Martes foina*	Negative	–	–	–	–	–	–	–	–	–	–	–	–	–	–	–	–	–	–	Negative	–	–	–	–	–	–	–	–	Not done	–	–	–
FMT6	CPW	Stone marten	*Martes foina*	Negative	–	–	–	–	–	–	–	–	–	–	–	–	–	–	–	–	–	–	Negative	–	–	–	–	–	–	–	–	Not done	–	–	–
FMT7	CPW	Stone marten	*Martes foina*	Negative	–	–	–	–	–	–	–	–	–	–	–	–	–	–	–	–	–	–	Negative	–	–	–	–	–	–	–	–	Not done	–	–	–
FMT8	CPW	Stone marten	*Martes foina*	Negative	–	–	–	–	–	–	–	–	–	–	–	–	–	–	–	–	–	–	Negative	–	–	–	–	–	–	–	–	Not done	–	–	–
VAMT7	CPW	Cattle	*Bos taurus*	Negative	–	–	–	–	–	–	–	–	–	–	–	–	–	–	–	–	–	–	Negative	–	–	–	–	–	–	–	–	Negative	–	–	–
VAMT8	CPW	Cattle	*Bos taurus*	Negative	–	–	–	–	–	–	–	–	–	–	–	–	–	–	–	–	–	–	Negative	–	–	–	–	–	–	–	–	Negative	–	–	–
CAFA8	CPW	Goat	*Capra hircus*	Negative	–	–	–	–	–	–	–	–	–	–	–	–	–	–	–	–	–	–	Negative	–	–	–	–	–	–	–	–	Negative	–	–	–
OVMT6	CPW	Sheep	*Ovis aries*	Negative	–	–	–	–	–	–	–	–	–	–	–	–	–	–	–	–	–	–	Negative	–	–	–	–	–	–	–	–	Negative	–	–	–
OVMT7	CPW	Sheep	*Ovis aries*	Negative	–	–	–	–	–	–	–	–	–	–	–	–	–	–	–	–	–	–	Negative	–	–	–	–	–	–	–	–	Negative	–	–	–
OVMT8	CPW	Sheep	*Ovis aries*	Negative	–	–	–	–	–	–	–	–	–	–	–	–	–	–	–	–	–	–	Negative	–	–	–	–	–	–	–	–	Negative	–	–	–
OVMT9	CPW	Sheep	*Ovis aries*	Negative	–	–	–	–	–	–	–	–	–	–	–	–	–	–	–	–	–	–	Negative	–	–	–	–	–	–	–	–	Negative	–	–	–
OVFA1	CPW	Sheep	*Ovis aries*	Negative	–	–	–	–	–	–	–	–	–	–	–	–	–	–	–	–	–	–	Negative	*Colpodella* sp.	–	–	–	–	–	–	–	Negative	–	–	–
OVFA2	CPW	Sheep	*Ovis aries*	Negative	–	–	–	–	–	–	–	–	–	–	–	–	–	–	–	–	–	–	Negative	–	–	–	–	–	–	–	–	Negative	–	–	–
OVFA3	CPW	Sheep	*Ovis aries*	Negative	–	–	–	–	–	–	–	–	–	–	–	–	–	–	–	–	–	–	Negative	–	–	–	–	–	–	–	–	Negative	–	–	–
OVFA4	CPW	Sheep	*Ovis aries*	Negative	–	–	–	–	–	–	–	–	–	–	–	–	–	–	–	–	–	–	Negative	–	–	–	–	–	–	–	–	Negative	–	–	–
OVFA5	CPW	Sheep	*Ovis aries*	Negative	–	–	–	–	–	–	–	–	–	–	–	–	–	–	–	–	–	–	Negative	–	–	–	–	–	–	–	–	Negative	–	–	–
VAFA7	CPW	Cattle	*Bos taurus*	Negative	–	–	–	–	–	–	–	–	–	–	–	–	–	–	–	–	–	–	Negative	–	–	–	–	–	–	–	–	Negative	–	–	–
VAFA8	CPW	Cattle	*Bos taurus*	**Positive**	36.3	Not done	–	–	–	Not done	–	–	–	Not done	–	–	–	Not done	–	–	–	Unknown	Negative	–	–	–	–	–	–	–	–	Negative	–	–	–
VAFA9	CPW	Cattle	*Bos taurus*	Negative	–	–	–	–	–	–	–	–	–	–	–	–	–	–	–	–	–	–	Negative	–	–	–	–	–	–	–	–	Negative	–	–	–
VAFA10	CPW	Cattle	*Bos taurus*	Negative	–	–	–	–	–	–	–	–	–	–	–	–	–	–	–	–	–	–	Negative	*Colpodella tetrahymenae*	–	–	–	–	–	–	–	Negative	–	–	–
VAMT9	CPW	Cattle	*Bos taurus*	**Positive**	36.4	Not done	–	–	–	Not done	–	–	–	Not done	–	–	–	Not done	–	–	–	Unknown	Negative	–	–	–	–	–	–	–	–	Negative	–	–	–
VAMT10	CPW	Cattle	*Bos taurus*	Negative	–	–	–	–	–	–	–	–	–	–	–	–	–	–	–	–	–	–	Negative	–	–	–	–	–	–	–	–	Negative	–	–	–
OVMT10	CPW	Sheep	*Ovis aries*	Negative	–	–	–	–	–	–	–	–	–	–	–	–	–	–	–	–	–	–	Negative	–	–	–	–	–	–	–	–	Negative	–	–	–
OVMT11	CPW	Sheep	*Ovis aries*	**Positive**	35.3	Not done	–	–	–	Not done	–	–	–	Not done	–	–	–	Not done	–	–	–	Unknown	Negative	–	–	–	–	–	–	–	–	Negative	–	–	–
OVMT12	CPW	Sheep	*Ovis aries*	**Positive**	35.5	Not done	–	–	–	Not done	–	–	–	Not done	–	–	–	Not done	–	–	–	Unknown	Negative	–	–	–	–	–	–	–	–	Negative	–	–	–
OVMT13	CPW	Sheep	*Ovis aries*	**Positive**	23.7	**Positive**	**E**	None	Yes	Negative	–	–	–	Negative	–	–	–	Negative	–	–	–	**E**	Negative	–	–	–	–	–	–	–	–	Negative	–	–	–
OVMT14	CPW	Sheep	*Ovis aries*	Negative	–	–	–	–	–	–	–	–	–	–	–	–	–	–	–	–	–	–	Negative	–	–	–	–	–	–	–	–	Negative	–	–	–

FFA4	CPW	Stone marten	*Martes foina*	Negative	–	–	–	–	–	–	–	–	–	–	–	–	–	–	–	–	–	–	Negative	–	–	–	–	–	–	–	–	Not done	–	–	–
JFA2	CPW	Wild boar	*Sus scrofa*	Negative	–	–	–	–	–	–	–	–	–	–	–	–	–	–	–	–	–	–	Negative	–	–	–	–	–	–	–	–	Negative	–	–	–
JFA3	CPW	Wild boar	*Sus scrofa*	Negative	–	–	–	–	–	–	–	–	–	–	–	–	–	–	–	–	–	–	Negative	–	–	–	–	–	–	–	–	Negative	–	–	–
JFA4	CPW	Wild boar	*Sus scrofa*	Negative	–	–	–	–	–	–	–	–	–	–	–	–	–	–	–	–	–	–	Negative	–	–	–	–	–	–	–	–	Negative	–	–	–
JFA5	CPW	Wild boar	*Sus scrofa*	Negative	–	–	–	–	–	–	–	–	–	–	–	–	–	–	–	–	–	–	Negative	–	–	–	–	–	–	–	–	Negative	–	–	–
GM186	CPW	Iberian wolf	*Canis lupus signatus*	Negative	–	–	–	–	–	–	–	–	–	–	–	–	–	–	–	–	–	–	Negative	–	–	–	–	–	–	–	–	Not done	–	–	–
GM187	CPW	Domestic dog	*Canis lupus familiaris*	Positive	29.6	Positive	D	None	Yes	Negative	–	–	–	Negative	–	–	–	Negative	–	–	–	D	Negative	–	–	–	–	–	–	–	–	Not done	–	–	–
GM188	CPW	Domestic dog	*Canis lupus familiaris*	Positive	29.9	Negative	–	–	–	Negative	–	–	–	Negative	–	–	–	Negative	–	–	–	Unknown	Negative	–	–	–	–	–	–	–	–	Not done	–	–	–
GM189	CPW	Domestic dog	*Canis lupus familiaris*	Positive	32.9	Not done	–	–	–	Not done	–	–	–	Not done	–	–	–	Not done	–	–	–	Unknown	Negative	–	–	–	–	–	–	–	–	Not done	–	–	–
GM190	CPW	Domestic dog	*Canis lupus familiaris*	Negative	–	–	–	–	–	–	–	–	–	–	–	–	–	–	–	–	–	–	Negative	–	–	–	–	–	–	–	–	Negative	–	–	–
GM191	CPW	Iberian wolf	*Canis lupus signatus*	Negative	–	–	–	–	–	–	–	–	–	–	–	–	–	–	–	–	–	–	Negative	–	–	–	–	–	–	–	–	Negative	–	–	–
GM192	CPW	Iberian wolf	*Canis lupus signatus*	Positive	35.4	Not done	–	–	–	Not done	–	–	–	Not done	–	–	–	Not done	–	–	–	Unknown	Negative	–	–	–	–	–	–	–	–	Negative	–	–	–
GM193	CPW	Iberian wolf	*Canis lupus signatus*	Negative	–	–	–	–	–	–	–	–	–	–	–	–	–	–	–	–	–	–	Negative	*Sordaria* sp.	–	–	–	–	–	–	–	Negative	–	–	–
GA98	CPW	Iberian wolf	*Canis lupus signatus*	Negative	–	–	–	–	–	–	–	–	–	–	–	–	–	–	–	–	–	–	Negative	–	–	–	–	–	–	–	–	Negative	–	–	–
GA99	CPW	Iberian wolf	*Canis lupus signatus*	Positive	34.3	Negative	–	–	–	–	–	–	–	–	–	–	–	–	–	–	–	–	Negative	–	–	–	–	–	–	–	–	Negative	–	–	–
GM194	CPW	Red fox	*Vulpes vulpes*	Negative	–	–	–	–	–	–	–	–	–	–	–	–	–	–	–	–	–	–	Negative	–	–	–	–	–	–	–	–	Negative	–	–	–
GM195	CPW	Red fox	*Vulpes vulpes*	Negative	–	–	–	–	–	–	–	–	–	–	–	–	–	–	–	–	–	–	Negative	–	–	–	–	–	–	–	–	Negative	–	–	–
GM196	CPW	Iberian wolf	*Canis lupus signatus*	Negative	–	–	–	–	–	–	–	–	–	–	–	–	–	–	–	–	–	–	Negative	–	–	–	–	–	–	–	–	Negative	–	–	–
GA100	CPW	Iberian wolf	*Canis lupus signatus*	Negative	–	–	–	–	–	–	–	–	–	–	–	–	–	–	–	–	–	–	Positive	*C. canis*	None	Yes	Untypable	–	–	–	*C. canis*	Negative	–	–	–
GA101	CPW	Iberian wolf	*Canis lupus signatus*	Negative	–	–	–	–	–	–	–	–	–	–	–	–	–	–	–	–	–	–	Negative	–	–	–	–	–	–	–	–	Negative	–	–	–
GA102	CPW	Iberian wolf	*Canis lupus signatus*	Negative	–	–	–	–	–	–	–	–	–	–	–	–	–	–	–	–	–	–	Negative	–	–	–	–	–	–	–	–	Negative	–	–	–
GA103	CPW	Iberian wolf	*Canis lupus signatus*	Negative	–	–	–	–	–	–	–	–	–	–	–	–	–	–	–	–	–	–	Negative	–	–	–	–	–	–	–	–	Negative	–	–	–
GA104	CPW	Iberian wolf	*Canis lupus signatus*	Positive	22.6	Positive	D	None	Yes	Negative	–	–	–	Positive	D	A159R, A201G, C207Y	Yes	Negative	–	–	–	D	Negative	–	–	–	–	–	–	–	–	Negative	–	–	–
GA105	CPW	Iberian wolf	*Canis lupus signatus*	Negative	–	–	–	–	–	–	–	–	–	–	–	–	–	–	–	–	–	–	Negative	–	–	–	–	–	–	–	–	Negative	–	–	–
GA106	CPW	Red fox	*Vulpes vulpes*	Negative	–	–	–	–	–	–	–	–	–	–	–	–	–	–	–	–	–	–	Negative	–	–	–	–	–	–	–	–	Negative	–	–	–
GA107	CPW	Iberian wolf	*Canis lupus signatus*	Positive	35.4	Not done	–	–	–	Not done	–	–	–	Not done	–	–	–	Not done	–	–	–	Unknown	Negative	–	–	–	–	–	–	–	–	Negative	–	–	–
GA108	CPW	Iberian wolf	*Canis lupus signatus*	Positive	35.4	Not done	–	–	–	Not done	–	–	–	Not done	–	–	–	Not done	–	–	–	Unknown	Negative	–	–	–	–	–	–	–	–	Negative	–	–	–
GA109	CPW	Iberian wolf	*Canis lupus signatus*	Negative	–	–	–	–	–	–	–	–	–	–	–	–	–	–	–	–	–	–	Negative	–	–	–	–	–	–	–	–	Negative	–	–	–
GA110	CPW	Iberian wolf	*Canis lupus signatus*	Negative	–	–	–	–	–	–	–	–	–	–	–	–	–	–	–	–	–	–	Negative	–	–	–	–	–	–	–	–	Negative	–	–	–
GA111	CPW	Iberian wolf	*Canis lupus signatus*	Negative	–	–	–	–	–	–	–	–	–	–	–	–	–	–	–	–	–	–	Negative	–	–	–	–	–	–	–	–	Negative	–	–	–
GA112	CPW	Iberian wolf	*Canis lupus signatus*	Negative	–	–	–	–	–	–	–	–	–	–	–	–	–	–	–	–	–	–	Negative	–	–	–	–	–	–	–	–	Negative	–	–	–
GM197	CPW	Domestic dog	*Canis lupus familiaris*	Positive	34.1	Negative	–	–	–	Not done	–	–	–	Not done	–	–	–	Not done	–	–	–	Unknown	Negative	–	–	–	–	–	–	–	–	Negative	–	–	–
GM198	CPW	Domestic dog	*Canis lupus familiaris*	Negative	–	–	–	–	–	–	–	–	–	–	–	–	–	–	–	–	–	–	Negative	–	–	–	–	–	–	–	–	Negative	–	–	–
GM199	CPW	Domestic dog	*Canis lupus familiaris*	Negative	–	–	–	–	–	–	–	–	–	–	–	–	–	–	–	–	–	–	Negative	–	–	–	–	–	–	–	–	Negative	–	–	–
GM200	CPW	Domestic dog	*Canis lupus familiaris*	Negative	–	–	–	–	–	–	–	–	–	–	–	–	–	–	–	–	–	–	Negative	–	–	–	–	–	–	–	–	Negative	–	–	–
GM201	CPW	Iberian wolf	*Canis lupus signatus*	Negative	–	–	–	–	–	–	–	–	–	–	–	–	–	–	–	–	–	–	Negative	–	–	–	–	–	–	–	–	Negative	–	–	–
GM203	CPW	Iberian wolf	*Canis lupus signatus*	Negative	–	–	–	–	–	–	–	–	–	–	–	–	–	–	–	–	–	–	Negative	*Hemiurosomoida warren*	–	–	–	–	–	–	–	Negative	–	–	–
GA113	CPW	Iberian wolf	*Canis lupus signatus*	Negative	–	–	–	–	–	–	–	–	–	–	–	–	–	–	–	–	–	–	Negative	–	–	–	–	–	–	–	–	Negative	–	–	–
GA114	CPW	Iberian wolf	*Canis lupus signatus*	Negative	–	–	–	–	–	–	–	–	–	–	–	–	–	–	–	–	–	–	Negative	–	–	–	–	–	–	–	–	Negative	–	–	–
GA115	CPW	Iberian wolf	*Canis lupus signatus*	Negative	–	–	–	–	–	–	–	–	–	–	–	–	–	–	–	–	–	–	Negative	–	–	–	–	–	–	–	–	Negative	–	–	–
GA116	CPW	Domestic dog	*Canis lupus familiaris*	Negative	–	–	–	–	–	–	–	–	–	–	–	–	–	–	–	–	–	–	Negative	–	–	–	–	–	–	–	–	Negative	–	–	–
GA117	CPW	Domestic dog	*Canis lupus familiaris*	Negative	–	–	–	–	–	–	–	–	–	–	–	–	–	–	–	–	–	–	Negative	–	–	–	–	–	–	–	–	Negative	–	–	–
GA118A	CPW	Iberian wolf	*Canis lupus signatus*	Positive	29.4	Negative	–	–	–	Negative	–	–	–	Negative	–	–	–	Negative	–	–	–	Unknown	Negative	–	–	–	–	–	–	–	–	Negative	–	–	–
GM204	CPW	Iberian wolf	*Canis lupus signatus*	Negative	–	–	–	–	–	–	–	–	–	–	–	–	–	–	–	–	–	–	Negative	–	–	–	–	–	–	–	–	Negative	–	–	–
GM205	CPW	Red fox	*Vulpes vulpes*	Negative	–	–	–	–	–	–	–	–	–	–	–	–	–	–	–	–	–	–	Negative	–	–	–	–	–	–	–	–	Negative	–	–	–
GA118B	CPW	Iberian wolf	*Canis lupus signatus*	Negative	–	–	–	–	–	–	–	–	–	–	–	–	–	–	–	–	–	–	Negative	–	–	–	–	–	–	–	–	Negative	–	–	–
GA120	CPW	Iberian wolf	*Canis lupus signatus*	Negative	–	–	–	–	–	–	–	–	–	–	–	–	–	–	–	–	–	–	Negative	–	–	–	–	–	–	–	–	Negative	–	–	–
GA121	CPW	Iberian wolf	*Canis lupus signatus*	Negative	–	–	–	–	–	–	–	–	–	–	–	–	–	–	–	–	–	–	Negative	–	–	–	–	–	–	–	–	Negative	–	–	–
GA122	CPW	Domestic dog	*Canis lupus familiaris*	Negative	–	–	–	–	–	–	–	–	–	–	–	–	–	–	–	–	–	–	Negative	–	–	–	–	–	–	–	–	Negative	–	–	–
GA123	CPW	Red fox	*Vulpes vulpes*	Negative	–	–	–	–	–	–	–	–	–	–	–	–	–	–	–	–	–	–	Negative	–	–	–	–	–	–	–	–	Negative	–	–	–
GA125	CPW	Iberian wolf	*Canis lupus signatus*	Negative	–	–	–	–	–	–	–	–	–	–	–	–	–	–	–	–	–	–	Negative	–	–	–	–	–	–	–	–	Negative	–	–	–
GA126	CPW	Iberian wolf	*Canis lupus signatus*	Negative	–	–	–	–	–	–	–	–	–	–	–	–	–	–	–	–	–	–	Negative	–	–	–	–	–	–	–	–	Negative	–	–	–
GM206	CPW	Red fox	*Vulpes vulpes*	Positive	38.5	Not done	–	–	–	Not done	–	–	–	Not done	–	–	–	Not done	–	–	–	Unknown	Negative	–	–	–	–	–	–	–	–	Negative	–	–	–
GM207	CPW	Iberian wolf	*Canis lupus signatus*	Negative	–	–	–	–	–	–	–	–	–	–	–	–	–	–	–	–	–	–	Negative	Uncultured eukaryote	–	–	–	–	–	–	–	Negative	–	–	–
GM209	CPW	Red fox	*Vulpes vulpes*	Negative	–	–	–	–	–	–	–	–	–	–	–	–	–	–	–	–	–	–	Negative	–	–	–	–	–	–	–	–	Negative	–	–	–
GM210	CPW	Red fox	*Vulpes vulpes*	Negative	–	–	–	–	–	–	–	–	–	–	–	–	–	–	–	–	–	–	Negative	–	–	–	–	–	–	–	–	Negative	–	–	–
GM211	CPW	Domestic dog	*Canis lupus familiaris*	Negative	–	–	–	–	–	–	–	–	–	–	–	–	–	–	–	–	–	–	Negative	–	–	–	–	–	–	–	–	Negative	–	–	–
GA127	CPW	Iberian wolf	*Canis lupus signatus*	Negative	–	–	–	–	–	–	–	–	–	–	–	–	–	–	–	–	–	–	Negative	–	–	–	–	–	–	–	–	Negative	–	–	–
GA128	CPW	Red fox	*Vulpes vulpes*	Negative	–	–	–	–	–	–	–	–	–	–	–	–	–	–	–	–	–	–	Negative	–	–	–	–	–	–	–	–	Negative	–	–	–
GA130	CPW	Iberian wolf	*Canis lupus signatus*	Negative	–	–	–	–	–	–	–	–	–	–	–	–	–	–	–	–	–	–	Negative	–	–	–	–	–	–	–	–	Negative	–	–	–
GA131	CPW	Iberian wolf	*Canis lupus signatus*	Negative	–	–	–	–	–	–	–	–	–	–	–	–	–	–	–	–	–	–	Negative	–	–	–	–	–	–	–	–	Negative	–	–	–
GA135	CPW	Iberian wolf	*Canis lupus signatus*	Negative	–	–	–	–	–	–	–	–	–	–	–	–	–	–	–	–	–	–	Negative	*Coniochaetales* sp.	–	–	–	–	–	–	–	Negative	–	–	–
GA137	CPW	Iberian wolf	*Canis lupus signatus*	Negative	–	–	–	–	–	–	–	–	–	–	–	–	–	–	–	–	–	–	Negative	–	–	–	–	–	–	–	–	Negative	–	–	–
GA138	CPW	Iberian wolf	*Canis lupus signatus*	Negative	–	–	–	–	–	–	–	–	–	–	–	–	–	–	–	–	–	–	Negative	–	–	–	–	–	–	–	–	Negative	–	–	–
GA139	CPW	Iberian wolf	*Canis lupus signatus*	Negative	–	–	–	–	–	–	–	–	–	–	–	–	–	–	–	–	–	–	Negative	–	–	–	–	–	–	–	–	Negative	–	–	–
GM213	CPW	Iberian wolf	*Canis lupus signatus*	Negative	–	–	–	–	–	–	–	–	–	–	–	–	–	–	–	–	–	–	Negative	–	–	–	–	–	–	–	–	Negative	–	–	–
GA140	CPW	Iberian wolf	*Canis lupus signatus*	Negative	–	–	–	–	–	–	–	–	–	–	–	–	–	–	–	–	–	–	Negative	–	–	–	–	–	–	–	–	Negative	–	–	–
GM214	CPW	Red fox	*Vulpes vulpes*	Negative	–	–	–	–	–	–	–	–	–	–	–	–	–	–	–	–	–	–	Negative	Uncultured fungus	–	–	–	–	–	–	–	Negative	–	–	–
GA142	CPW	Iberian wolf	*Canis lupus signatus*	Positive	30.8	Positive	D	C262Y	Yes	Positive	C	C207T	Yes	Negative	–	–	–	Negative	–	–	–	C+D	Negative	–	–	–	–	–	–	–	–	Negative	–	–	–
GA143	CPW	Red fox	*Vulpes vulpes*	Positive	31.9	Negative	–	–	–	Negative	–	–	–	Negative	–	–	–	Negative	–	–	–	Unknown	Negative	–	–	–	–	–	–	–	–	Negative	–	–	–
GA144	CPW	Iberian wolf	*Canis lupus signatus*	Negative	–	–	–	–	–	–	–	–	–	–	–	–	–	–	–	–	–	–	Negative	–	–	–	–	–	–	–	–	Negative	–	–	–
GM215	CPW	Red fox	*Vulpes vulpes*	Positive	34.5	Negative	–	–	–	–	–	–	–	–	–	–	–	–	–	–	–	–	Negative	–	–	–	–	–	–	–	–	Negative	–	–	–
GM216	CPW	Domestic dog	*Canis lupus familiaris*	Positive	37.5	Not done	–	–	–	Not done	–	–	–	Not done	–	–	–	Not done	–	–	–	Unknown	Negative	Uncultured eukaryote	–	–	–	–	–	–	–	Negative	–	–	–
GM217	CPW	Red fox	*Vulpes vulpes*	Negative	–	–	–	–	–	–	–	–	–	–	–	–	–	–	–	–	–	–	Negative	–	–	–	–	–	–	–	–	Negative	–	–	–
GM218	CPW	Red fox	*Vulpes vulpes*	Negative	–	–	–	–	–	–	–	–	–	–	–	–	–	–	–	–	–	–	Negative	–	–	–	–	–	–	–	–	Negative	–	–	–
GM219	CPW	Red fox	*Vulpes vulpes*	Negative	–	–	–	–	–	–	–	–	–	–	–	–	–	–	–	–	–	–	Negative	–	–	–	–	–	–	–	–	Negative	–	–	–
GM220	CPW	Red fox	*Vulpes vulpes*	Positive	25.1	Positive	D	None	Yes	Positive	C	None	Yes	Positive	C	C217T	Yes	Negative	–	–	–	C+D	Negative	–	–	–	–	–	–	–	–	Negative	–	–	–
GM222	CPW	Red fox	*Vulpes vulpes*	Negative	–	–	–	–	–	–	–	–	–	–	–	–	–	–	–	–	–	–	Negative	–	–	–	–	–	–	–	–	Negative	–	–	–
GM223	CPW	Red fox	*Vulpes vulpes*	Negative	–	–	–	–	–	–	–	–	–	–	–	–	–	–	–	–	–	–	Negative	–	–	–	–	–	–	–	–	Negative	–	–	–
GM224	CPW	Red fox	*Vulpes vulpes*	Negative	–	–	–	–	–	–	–	–	–	–	–	–	–	–	–	–	–	–	Negative	–	–	–	–	–	–	–	–	Negative	–	–	–
GM226	CPW	Red fox	*Vulpes vulpes*	Negative	–	–	–	–	–	–	–	–	–	–	–	–	–	–	–	–	–	–	Negative	–	–	–	–	–	–	–	–	Negative	–	–	–
GM227	CPW	Red fox	*Vulpes vulpes*	Negative	–	–	–	–	–	–	–	–	–	–	–	–	–	–	–	–	–	–	Negative	–	–	–	–	–	–	–	–	Negative	–	–	–
GM228	CPW	Red fox	*Vulpes vulpes*	Negative	–	–	–	–	–	–	–	–	–	–	–	–	–	–	–	–	–	–	Negative	–	–	–	–	–	–	–	–	Negative	–	–	–
GM289	CPW	Red fox	*Vulpes vulpes*	Negative	–	–	–	–	–	–	–	–	–	–	–	–	–	–	–	–	–	–	Negative	–	–	–	–	–	–	–	–	Negative	–	–	–
GM290	CPW	Red fox	*Vulpes vulpes*	Negative	–	–	–	–	–	–	–	–	–	–	–	–	–	–	–	–	–	–	Negative	–	–	–	–	–	–	–	–	Negative	–	–	–
GM291	CPW	Red fox	*Vulpes vulpes*	Negative	–	–	–	–	–	–	–	–	–	–	–	–	–	–	–	–	–	–	Negative	–	–	–	–	–	–	–	–	Negative	–	–	–
GM293	CPW	Red fox	*Vulpes vulpes*	Negative	–	–	–	–	–	–	–	–	–	–	–	–	–	–	–	–	–	–	Negative	–	–	–	–	–	–	–	–	Negative	–	–	–
GM294	CPW	Red fox	*Vulpes vulpes*	Positive	37.8	Not done	–	–	–	Not done	–	–	–	Not done	–	–	–	Not done	–	–	–	Unknown	Negative	–	–	–	–	–	–	–	–	Negative	–	–	–
GM296	CPW	Red fox	*Vulpes vulpes*	Negative	–	–	–	–	–	–	–	–	–	–	–	–	–	–	–	–	–	–	Negative	–	–	–	–	–	–	–	–	Negative	–	–	–
GM298	CPW	Red fox	*Vulpes vulpes*	Negative		–	–	–	–	–	–	–	–	–	–	–	–	–	–	–	–	–	Negative	–	–	–	–	–	–	–	–	Negative	–	–	–
GA146	CPW	Red fox	*Vulpes vulpes*	Negative	–	–	–	–	–	–	–	–	–	–	–	–	–	–	–	–	–	–	Negative	–	–	–	–	–	–	–	–	Negative	–	–	–
GA147	CPW	Red fox	*Vulpes vulpes*	Negative	–	–	–	–	–	–	–	–	–	–	–	–	–	–	–	–	–	–	Negative	–	–	–	–	–	–	–	–	Negative	–	–	–
GA148	CPW	Red fox	*Vulpes vulpes*	Negative	–	–	–	–	–	–	–	–	–	–	–	–	–	–	–	–	–	–	Negative	–	–	–	–	–	–	–	–	Negative	–	–	–
GA149	CPW	Iberian wolf	*Canis lupus signatus*	Positive	28.8	Positive	D	None	Yes	Positive	C	C207T	–	Negative	–	–	–	Negative	–	–	–	C+D	Negative	–	–	–	–	–	–	–	–	Negative	–	–	–
GA150	CPW	Iberian wolf	*Canis lupus signatus*	Positive	36.0	Not done	–	–	–	Not done	–	–	–	Not done	–	–	–	Not done	–	–	–	Unknown	Negative	–	–	–	–	–	–	–	–	Negative	–	–	–
GA153	CPW	Iberian wolf	*Canis lupus signatus*	Negative	–	–	–	–	–	–	–	–	–	–	–	–	–	–	–	–	–	–	Negative	–	–	–	–	–	–	–	–	Negative	–	–	–
GA154	CPW	Iberian wolf	*Canis lupus signatus*	Negative	–	–	–	–	–	–	–	–	–	–	–	–	–	–	–	–	–	–	Negative	–	–	–	–	–	–	–	–	Negative	–	–	–
GA155	CPW	Iberian wolf	*Canis lupus signatus*	Positive	36.5	Not done	–	–	–	Not done	–	–	–	Not done	–	–	–	Not done	–	–	–	Unknown	Negative	–	–	–	–	–	–	–	–	Negative	–	–	–
GA156	CPW	Iberian wolf	*Canis lupus signatus*	Negative	–	–	–	–	–	–	–	–	–	–	–	–	–	–	–	–	–	–	Negative	*Tetraploa* sp.	–	–	–	–	–	–	–	Negative	–	–	–
GA157	CPW	Red fox	*Vulpes vulpes*	Negative	–	–	–	–	–	–	–	–	–	–	–	–	–	–	–	–	–	–	Negative	*Tilletiopsis pallescens*	–	–	–	–	–	–	–	Negative	–	–	–
GA160	CPW	Iberian wolf	*Canis lupus signatus*	Negative	–	–	–	–	–	–	–	–	–	–	–	–	–	–	–	–	–	–	Negative	–	–	–	–	–	–	–	–	Negative	–	–	–
GA161	CPW	Iberian wolf	*Canis lupus signatus*	Negative	–	–	–	–	–	–	–	–	–	–	–	–	–	–	–	–	–	–	Negative	–	–	–	–	–	–	–	–	Negative	–	–	–
GA163	CPW	Domestic dog	*Canis lupus familiaris*	Negative	–	–	–	–	–	–	–	–	–	–	–	–	–	–	–	–	–	–	Negative	–	–	–	–	–	–	–	–	Negative	–	–	–
GA164	CPW	Domestic dog	*Canis lupus familiaris*	Negative	–	–	–	–	–	–	–	–	–	–	–	–	–	–	–	–	–	–	Negative	*Tilletiopsis pallescens*	–	–	–	–	–	–	–	Negative	–	–	–
JWFL1	CPW	Wild boar	*Sus scrofa*	Negative	–	–	–	–	–	–	–	–	–	–	–	–	–	–	–	–	–	–	Negative	–	–	–	–	–	–	–	–	Negative	–	–	–
JWFL2	CPW	Wild boar	*Sus scrofa*	Negative	–	–	–	–	–	–	–	–	–	–	–	–	–	–	–	–	–	–	Negative	–	–	–	–	–	–	–	–	Negative	–	–	–
JWFL3	CPW	Wild boar	*Sus scrofa*	Negative	–	–	–	–	–	–	–	–	–	–	–	–	–	–	–	–	–	–	Negative	–	–	–	–	–	–	–	–	Negative	–	–	–
JWFL4	CPW	Wild boar	*Sus scrofa*	Negative	–	–	–	–	–	–	–	–	–	–	–	–	–	–	–	–	–	–	Negative	–	–	–	–	–	–	–	–	Negative	–	–	–
JWFL5	CPW	Wild boar	*Sus scrofa*	Negative	–	–	–	–	–	–	–	–	–	–	–	–	–	–	–	–	–	–	Negative	–	–	–	–	–	–	–	–	Negative	–	–	–
JWFL6	CPW	Wild boar	*Sus scrofa*	Negative	–	–	–	–	–	–	–	–	–	–	–	–	–	–	–	–	–	–	Negative	–	–	–	–	–	–	–	–	Negative	–	–	–
JWFL7	CPW	Wild boar	*Sus scrofa*	Positive	38.5	Not done	–	–	–	Not done	–	–	–	Not done	–	–	–	Not done	–	–	–	Unknown	Negative	–	–	–	–	–	–	–	–	Negative	–	–	–

WFL041	CPW	Iberian wolf	*Canis lupus signatus*	Negative	–	–	–	–	–	–	–	–	–	–	–	–	–	–	–	–	–	–	Negative	–	–	–	–	–	–	–	–	Not done	–	–	–
WFL043	CPW	Domestic dog	*Canis lupus familiaris*	Negative	–	–	–	–	–	–	–	–	–	–	–	–	–	–	–	–	–	–	Negative	–	–	–	–	–	–	–	–	Not done	–	–	–
WFL044	CPE	Iberian wolf	*Canis lupus signatus*	Negative	–	–	–	–	–	–	–	–	–	–	–	–	–	–	–	–	–	–	Negative	–	–	–	–	–	–	–	–	Not done	–	–	–
WFL053	CPE	Iberian wolf	*Canis lupus signatus*	Negative	–	–	–	–	–	–	–	–	–	–	–	–	–	–	–	–	–	–	Negative	–	–	–	–	–	–	–	–	Not done	–	–	–
WFL055	CPE	Domestic dog	*Canis lupus familiaris*	**Positive**	34.3	Negative	–	–	–	Not done	–	–	–	Not done	–	–	–	Not done	–	–	–	Unknown	Negative	–	–	–	–	–	–	–	–	Not done	–	–	–
WFL060	CPE	Domestic dog	*Canis lupus familiaris*	Negative	–	–	–	–	–	–	–	–	–	–	–	–	–	–	–	–	–	–	Negative	–	–	–	–	–	–	–	–	Not done	–	–	–
WFL064	CPW	Iberian wolf	*Canis lupus signatus*	Negative	–	–	–	–	–	–	–	–	–	–	–	–	–	–	–	–	–	–	Negative	–	–	–	–	–	–	–	–	Not done	–	–	–
WFL065	CPW	Iberian wolf	*Canis lupus signatus*	Negative	–	–	–	–	–	–	–	–	–	–	–	–	–	–	–	–	–	–	Negative	–	–	–	–	–	–	–	–	Not done	–	–	–
WFL076	CPE	Red fox	*Vulpes vulpes*	Negative	–	–	–	–	–	–	–	–	–	–	–	–	–	–	–	–	–	–	Negative	–	–	–	–	–	–	–	–	Not done	–	–	–
WFL079	CPE	Domestic dog	*Canis lupus familiaris*	Negative	–	–	–	–	–	–	–	–	–	–	–	–	–	–	–	–	–	–	Negative	–	–	–	–	–	–	–	–	Not done	–	–	–
WFL080	CPE	Domestic dog	*Canis lupus familiaris*	Negative	–	–	–	–	–	–	–	–	–	–	–	–	–	–	–	–	–	–	Negative	–	–	–	–	–	–	–	–	Not done	–	–	–
WFL084	CPW	Red fox	*Vulpes vulpes*	Negative	–	–	–	–	–	–	–	–	–	–	–	–	–	–	–	–	–	–	Negative	–	–	–	–	–	–	–	–	Not done	–	–	–
WFL085	CPW	Iberian wolf	*Canis lupus signatus*	Negative	–	–	–	–	–	–	–	–	–	–	–	–	–	–	–	–	–	–	Negative	–	–	–	–	–	–	–	–	Not done	–	–	–
WFL088	CPW	Iberian wolf	*Canis lupus signatus*	Negative	–	–	–	–	–	–	–	–	–	–	–	–	–	–	–	–	–	–	Negative	Uncultured fungus	–	–	–	–	–	–	–	Not done	–	–	–
WFL089	CPE	Iberian wolf	*Canis lupus signatus*	Negative	–	–	–	–	–	–	–	–	–	–	–	–	–	–	–	–	–	–	Negative	–	–	–	–	–	–	–	–	Not done	–	–	–
WFL091	CPE	Iberian wolf	*Canis lupus signatus*	Negative	–	–	–	–	–	–	–	–	–	–	–	–	–	–	–	–	–	–	Negative	*Sordaria fimicola*	–	–	–	–	–	–	–	Not done	–	–	–
WFL093	CPE	Iberian wolf	*Canis lupus signatus*	Negative	–	–	–	–	–	–	–	–	–	–	–	–	–	–	–	–	–	–	Negative	–	–	–	–	–	–	–	–	Not done	–	–	–
WFL094	CPE	Red fox	*Vulpes vulpes*	Negative	–	–	–	–	–	–	–	–	–	–	–	–	–	–	–	–	–	–	Negative	–	–	–	–	–	–	–	–	Not done	–	–	–
WFL095	CPE	Domestic dog	*Canis lupus familiaris*	Negative	–	–	–	–	–	–	–	–	–	–	–	–	–	–	–	–	–	–	Negative	–	–	–	–	–	–	–	–	Not done	–	–	–
WFL096	CPE	Domestic dog	*Canis lupus familiaris*	Negative	–	–	–	–	–	–	–	–	–	–	–	–	–	–	–	–	–	–	Negative	*Sordaria* sp.	–	–	–	–	–	–	–	Not done	–	–	–
WFL098	CPE	Domestic dog	*Canis lupus familiaris*	**Positive**	38	Not done	–	–	–	Not done	–	–	–	Not done	–	–	–	Not done	–	–	–	Unknown	Negative	*Colpoda* sp.	–	–	–	–	–	–	–	Not done	–	–	–
WFL099	CPE	Domestic dog	*Canis lupus familiaris*	Negative	–	–	–	–	–	–	–	–	–	–	–	–	–	–	–	–	–	–	Negative	–	–	–	–	–	–	–	–	Not done	–	–	–
WFL101	CPE	Red fox	*Vulpes vulpes*	Negative	–	–	–	–	–	–	–	–	–	–	–	–	–	–	–	–	–	–	**Positive**	***C. canis***	686InsTG, T739A	Yes	Untypable	–	–	–	***C. canis***	Not done	–	–	–
WFL102	CPE	Domestic dog	*Canis lupus familiaris*	Negative	–	–	–	–	–	–	–	–	–	–	–	–	–	–	–	–	–	–	Negative	–	–	–	–	–	–	–	–	Not done	–	–	–
WFL110	CPE	Iberian wolf	*Canis lupus signatus*	Negative	–	–	–	–	–	–	–	–	–	–	–	–	–	–	–	–	–	–	Negative	*Sordaria* sp.	–	–	–	–	–	–	–	Not done	–	–	–
WFL111	CPE	Iberian wolf	*Canis lupus signatus*	Negative	–	–	–	–	–	–	–	–	–	–	–	–	–	–	–	–	–	–	Negative	–	–	–	–	–	–	–	–	Not done	–	–	–
WFL113	CPE	Domestic dog	*Canis lupus familiaris*	Negative	–	–	–	–	–	–	–	–	–	–	–	–	–	–	–	–	–	–	Negative	*Sordaria* sp.	–	–	–	–	–	–	–	Not done	–	–	–
WFL114	CPE	Iberian wolf	*Canis lupus signatus*	Negative	–	–	–	–	–	–	–	–	–	–	–	–	–	–	–	–	–	–	Negative	Uncultured eukaryote	–	–	–	–	–	–	–	Not done	–	–	–
WFL115	CPW	Iberian wolf	*Canis lupus signatus*	**Positive**	35.1	Not done	–	–	–	Not done	–	–	–	Not done	–	–	–	Not done	–	–	–	Unknown	Negative	–	–	–	–	–	–	–	–	Not done	–	–	–
WFL116	CPW	Iberian wolf	*Canis lupus signatus*	Negative	–	–	–	–	–	–	–	–	–	–	–	–	–	–	–	–	–	–	Negative	–	–	–	–	–	–	–	–	Not done	–	–	–
WFL117	CPW	Iberian wolf	*Canis lupus signatus*	Negative	–	–	–	–	–	–	–	–	–	–	–	–	–	–	–	–	–	–	Negative	–	–	–	–	–	–	–	–	Not done	–	–	–
WFL118	CPW	Iberian wolf	*Canis lupus signatus*	Negative	–	–	–	–	–	–	–	–	–	–	–	–	–	–	–	–	–	–	Negative	–	–	–	–	–	–	–	–	Not done	–	–	–
WFL119	CPE	Iberian wolf	*Canis lupus signatus*	Negative	–	–	–	–	–	–	–	–	–	–	–	–	–	–	–	–	–	–	Negative	–	–	–	–	–	–	–	–	Not done	–	–	–
WFL120	CPW	Iberian wolf	*Canis lupus signatus*	**Positive**	27.3	Negative	–	–	–	Negative	–	–	–	Negative	–	–	–	Negative	–	–	–	Unknown	Negative	–	–	–	–	–	–	–	–	Not done	–	–	–
WFL122	CPW	Domestic dog	*Canis lupus familiaris*	Negative	–	–	–	–	–	–	–	–	–	–	–	–	–	–	–	–	–	–	Negative	–	–	–	–	–	–	–	–	Not done	–	–	–
WFL123	CPW	Iberian wolf	*Canis lupus signatus*	Negative	–	–	–	–	–	–	–	–	–	–	–	–	–	–	–	–	–	–	Negative	–	–	–	–	–	–	–	–	Not done	–	–	–
WFL124	CPW	Iberian wolf	*Canis lupus signatus*	Negative	–	–	–	–	–	–	–	–	–	–	–	–	–	–	–	–	–	–	Negative	–	–	–	–	–	–	–	–	Not done	–	–	–
WFL125	CPW	Domestic dog	*Canis lupus familiaris*	**Positive**	36.5	Not done	–	–	–	Not done	–	–	–	Not done	–	–	–	Not done	–	–	–	Unknown	Negative	–	–	–	–	–	–	–	–	Not done	–	–	–
WFL126	CPW	Iberian wolf	*Canis lupus signatus*	**Positive**	31.6	Negative	–	–	–	Negative	–	–	–	Negative	–	–	–	Negative	–	–	–	Unknown	**Positive**	***C. canis***	None	Yes	Untypable	–	–	–	***C. canis***	Not done	–	–	–
WFL127	CPW	Domestic dog	*Canis lupus familiaris*	Negative	–	–	–	–	–	–	–	–	–	–	–	–	–	–	–	–	–	–	Negative	–	–	–	–	–	–	–	–	Not done	–	–	–
WFL128	CPW	Iberian wolf	*Canis lupus signatus*	Negative	–	–	–	–	–	–	–	–	–	–	–	–	–	–	–	–	–	–	Negative	–	–	–	–	–	–	–	–	Not done	–	–	–
WFL129	CPW	Iberian wolf	*Canis lupus signatus*	**Positive**	38.3	Not done	–	–	–	Not done	–	–	–	Not done	–	–	–	Not done	–	–	–	Unknown	Negative	–	–	–	–	–	–	–	–	Not done	–	–	–
WFL130	CPW	Iberian wolf	*Canis lupus signatus*	Negative	–	–	–	–	–	–	–	–	–	–	–	–	–	–	–	–	–	–	Negative	–	–	–	–	–	–	–	–	Not done	–	–	–
WFL131	CPE	Iberian wolf	*Canis lupus signatus*	Negative	–	–	–	–	–	–	–	–	–	–	–	–	–	–	–	–	–	–	Negative	–	–	–	–	–	–	–	–	Not done	–	–	–
WFL132	CPE	Iberian wolf	*Canis lupus signatus*	**Positive**	36.5	Not done	–	–	–	Not done	–	–	–	Not done	–	–	–	Not done	–	–	–	Unknown	Negative	–	–	–	–	–	–	–	–	Not done	–	–	–
WFL133	CPE	Domestic dog	*Canis lupus familiaris*	Negative	–	–	–	–	–	–	–	–	–	–	–	–	–	–	–	–	–	–	Negative	–	–	–	–	–	–	–	–	Not done	–	–	–
WFL134	CPE	Domestic dog	*Canis lupus familiaris*	Negative	–	–	–	–	–	–	–	–	–	–	–	–	–	–	–	–	–	–	Negative	–	–	–	–	–	–	–	–	Not done	–	–	–
WFL135	CPE	Domestic dog	*Canis lupus familiaris*	Negative	–	–	–	–	–	–	–	–	–	–	–	–	–	–	–	–	–	–	Negative	–	–	–	–	–	–	–	–	Not done	–	–	–
WFL137	CPE	Domestic dog	*Canis lupus familiaris*	Negative	–	–	–	–	–	–	–	–	–	–	–	–	–	–	–	–	–	–	Negative	–	–	–	–	–	–	–	–	Not done	–	–	–
WFL139	CPE	Domestic dog	*Canis lupus familiaris*	Negative	–	–	–	–	–	–	–	–	–	–	–	–	–	–	–	–	–	–	Negative	–	–	–	–	–	–	–	–	Not done	–	–	–
WFL142	CPW	Domestic dog	*Canis lupus familiaris*	**Positive**	33.8	Negative	–	–	–	Not done	–	–	–	Not done	–	–	–	Not done	–	–	–	Unknown	Negative	–	–	–	–	–	–	–	–	Not done	–	–	–
WFL144	CPW	Domestic dog	*Canis lupus familiaris*	Negative	–	–	–	–	–	–	–	–	–	–	–	–	–	–	–	–	–	–	Negative	–	–	–	–	–	–	–	–	Not done	–	–	–
WFL148	CPE	Domestic dog	*Canis lupus familiaris*	Negative	–	–	–	–	–	–	–	–	–	–	–	–	–	–	–	–	–	–	Negative	–	–	–	–	–	–	–	–	Not done	–	–	–
WFL150	CPE	Domestic dog	*Canis lupus familiaris*	Negative	–	–	–	–	–	–	–	–	–	–	–	–	–	–	–	–	–	–	Negative	*Preussia lignicola*	–	–	–	–	–	–	–	Not done	–	–	–
WFL151	CPE	Domestic dog	*Canis lupus familiaris*	Negative	–	–	–	–	–	–	–	–	–	–	–	–	–	–	–	–	–	–	Negative	–	–	–	–	–	–	–	–	Not done	–	–	–
WFL152	CPE	Iberian wolf	*Canis lupus signatus*	Negative	–	–	–	–	–	–	–	–	–	–	–	–	–	–	–	–	–	–	Negative	–	–	–	–	–	–	–	–	Not done	–	–	–
WFL153	CPE	Iberian wolf	*Canis lupus signatus*	Negative	–	–	–	–	–	–	–	–	–	–	–	–	–	–	–	–	–	–	Negative	–	–	–	–	–	–	–	–	Not done	–	–	–
WFL154	CPW	Iberian wolf	*Canis lupus signatus*	Negative	–	–	–	–	–	–	–	–	–	–	–	–	–	–	–	–	–	–	Negative	–	–	–	–	–	–	–	–	Not done	–	–	–
WFL155	CPW	Iberian wolf	*Canis lupus signatus*	**Positive**	29.2	Negative	–	–	–	Negative	–	–	–	Negative	–	–	–	Negative	–	–	–	Unknown	Negative	–	–	–	–	–	–	–	–	Not done	–	–	–
WFL158	CPW	Iberian wolf	*Canis lupus signatus*	Negative	–	–	–	–	–	–	–	–	–	–	–	–	–	–	–	–	–	–	Negative	–	–	–	–	–	–	–	–	Not done	–	–	–
WFL159	CPE	Domestic dog	*Canis lupus familiaris*	**Positive**	29.8	Negative	–	–	–	Negative	–	–	–	Negative	–	–	–	Negative	–	–	–	Unknown	Negative	–	–	–	–	–	–	–	–	Not done	–	–	–
WFL160	CPE	Domestic dog	*Canis lupus familiaris*	Negative	–	–	–	–	–	–	–	–	–	–	–	–	–	–	–	–	–	–	Negative	–	–	–	–	–	–	–	–	Not done	–	–	–
WFL173	CPE	Domestic dog	*Canis lupus familiaris*	Negative	–	–	–	–	–	–	–	–	–	–	–	–	–	–	–	–	–	–	Negative	–	–	–	–	–	–	–	–	Not done	–	–	–
WFL174	CPE	Domestic dog	*Canis lupus familiaris*	Negative	–	–	–	–	–	–	–	–	–	–	–	–	–	–	–	–	–	–	Negative	–	–	–	–	–	–	–	–	Not done	–	–	–
WFL179	CPE	Domestic dog	*Canis lupus familiaris*	Negative	–	–	–	–	–	–	–	–	–	–	–	–	–	–	–	–	–	–	Negative	–	–	–	–	–	–	–	–	Not done	–	–	–
WFL181	CPE	Domestic dog	*Canis lupus familiaris*	**Positive**	33.3	Negative	–	–	–	Not done	–	–	–	Not done	–	–	–	Not done	–	–	–	Unknown	Negative	–	–	–	–	–	–	–	–	Not done	–	–	–
WFL188	CPE	Domestic dog	*Canis lupus familiaris*	Negative	–	–	–	–	–	–	–	–	–	–	–	–	–	–	–	–	–	–	Negative	–	–	–	–	–	–	–	–	Not done	–	–	–
JMC1	CPE	Wild boar	*Sus scrofa*	Negative	–	–	–	–	–	–	–	–	–	–	–	–	–	–	–	–	–	–	Negative	–	–	–	–	–	–	–	–	Negative	–	–	–
JMC2	CPE	Wild boar	*Sus scrofa*	Negative	–	–	–	–	–	–	–	–	–	–	–	–	–	–	–	–	–	–	Negative	–	–	–	–	–	–	–	–	Negative	–	–	–
JMC3	CPE	Wild boar	*Sus scrofa*	Negative	–	–	–	–	–	–	–	–	–	–	–	–	–	–	–	–	–	–	Negative	–	–	–	–	–	–	–	–	Negative	–	–	–
JMC4	CPE	Wild boar	*Sus scrofa*	Negative	–	–	–	–	–	–	–	–	–	–	–	–	–	–	–	–	–	–	Negative	–	–	–	–	–	–	–	–	Negative	–	–	–
JMC5	CPE	Wild boar	*Sus scrofa*	Negative	–	–	–	–	–	–	–	–	–	–	–	–	–	–	–	–	–	–	Negative	–	–	–	–	–	–	–	–	Negative	–	–	–
JMC6	CPE	Wild boar	*Sus scrofa*	Negative	–	–	–	–	–	–	–	–	–	–	–	–	–	–	–	–	–	–	Negative	–	–	–	–	–	–	–	–	Negative	–	–	–
JMC7	CPE	Wild boar	*Sus scrofa*	Negative	–	–	–	–	–	–	–	–	–	–	–	–	–	–	–	–	–	–	Negative	–	–	–	–	–	–	–	–	Negative	–	–	–
JMC8	CPE	Wild boar	*Sus scrofa*	Negative	–	–	–	–	–	–	–	–	–	–	–	–	–	–	–	–	–	–	Negative	–	–	–	–	–	–	–	–	Negative	–	–	–
JMC9	CPE	Wild boar	*Sus scrofa*	Negative	–	–	–	–	–	–	–	–	–	–	–	–	–	–	–	–	–	–	Negative	–	–	–	–	–	–	–	–	Negative	–	–	–
JMC10	CPE	Wild boar	*Sus scrofa*	Negative	–	–	–	–	–	–	–	–	–	–	–	–	–	–	–	–	–	–	Negative	–	–	–	–	–	–	–	–	Negative	–	–	–
JMC11	CPE	Wild boar	*Sus scrofa*	Negative	–	–	–	–	–	–	–	–	–	–	–	–	–	–	–	–	–	–	Negative	–	–	–	–	–	–	–	–	Negative	–	–	–
JMC12	CPE	Wild boar	*Sus scrofa*	Negative	–	–	–	–	–	–	–	–	–	–	–	–	–	–	–	–	–	–	Negative	–	–	–	–	–	–	–	–	Negative	–	–	–
JMC13	CPE	Wild boar	*Sus scrofa*	Negative	–	–	–	–	–	–	–	–	–	–	–	–	–	–	–	–	–	–	Negative	–	–	–	–	–	–	–	–	Negative	–	–	–
JMC14	CPE	Wild boar	*Sus scrofa*	**Positive**	35	Negative	–	–	–	Not done	–	–	–	Not done	–	–	–	Not done	–	–	–	Unknown	Negative	–	–	–	–	–	–	–	–	Negative	–	–	–
JMC15	CPE	Wild boar	*Sus scrofa*	**Positive**	34.8	Negative	–	–	–	–	–	–	–	–	–	–	–	–	–	–	–	Unknown	Negative	–	–	–	–	–	–	–	–	Negative	–	–	–
JMC16	CPE	Wild boar	*Sus scrofa*	**Positive**	36.6	Not done	–	–	–	Not done	–	–	–	Not done	–	–	–	Not done	–	–	–	–	Negative	–	–	–	–	–	–	–	–	Negative	–	–	–
JMC17	CPE	Wild boar	*Sus scrofa*	Negative	–	–	–	–	–	–	–	–	–	–	–	–	–	–	–	–	–	–	Negative	–	–	–	–	–	–	–	–	Negative	–	–	–
JMC18	CPE	Wild boar	*Sus scrofa*	Negative	–	–	–	–	–	–	–	–	–	–	–	–	–	–	–	–	–	–	Negative	–	–	–	–	–	–	–	–	Negative	–	–	–
JMC19	CPE	Wild boar	*Sus scrofa*	**Positive**	28.4	Negative	–	–	–	Negative	–	–	–	Negative	–	–	–	Negative	–	–	–	Unknown	Negative	–	–	–	–	–	–	–	–	Negative	–	–	–
JMC20	CPE	Wild boar	*Sus scrofa*	Negative	–	–	–	–	–	–	–	–	–	–	–	–	–	–	–	–	–	–	Negative	–	–	–	–	–	–	–	–	Negative	–	–	–
JMC21	CPE	Wild boar	*Sus scrofa*	Negative	–	–	–	–	–	–	–	–	–	–	–	–	–	–	–	–	–	–	Negative	–	–	–	–	–	–	–	–	Negative	–	–	–
JMC22	CPE	Wild boar	*Sus scrofa*	Negative	–	–	–	–	–	–	–	–	–	–	–	–	–	–	–	–	–	–	Negative	–	–	–	–	–	–	–	–	Negative	–	–	–
JMC23	CPE	Wild boar	*Sus scrofa*	Negative	–	–	–	–	–	–	–	–	–	–	–	–	–	–	–	–	–	–	Negative	–	–	–	–	–	–	–	–	Negative	–	–	–
JMC24	CPE	Wild boar	*Sus scrofa*	Negative	–	–	–	–	–	–	–	–	–	–	–	–	–	–	–	–	–	–	Negative	–	–	–	–	–	–	–	–	Negative	–	–	–
JMC25	CPE	Wild boar	*Sus scrofa*	Negative	–	–	–	–	–	–	–	–	–	–	–	–	–	–	–	–	–	–	Negative	–	–	–	–	–	–	–	–	Negative	–	–	–
JMC26	CPE	Wild boar	*Sus scrofa*	Negative	–	–	–	–	–	–	–	–	–	–	–	–	–	–	–	–	–	–	Negative	–	–	–	–	–	–	–	–	Negative	–	–	–
JMC27	CPE	Wild boar	*Sus scrofa*	Negative	–	–	–	–	–	–	–	–	–	–	–	–	–	–	–	–	–	–	**Positive**	***C. scrofarum***	None	Yes	–	–	–	–	**C. scrofarum**	Negative	–	–	–
JMC28	CPE	Wild boar	*Sus scrofa*	Negative	–	–	–	–	–	–	–	–	–	–	–	–	–	–	–	–	–	–	Negative	–	–	–	–	–	–	–	–	Negative	–	–	–
JMC29	CPE	Wild boar	*Sus scrofa*	Negative	–	–	–	–	–	–	–	–	–	–	–	–	–	–	–	–	–	–	Negative	–	–	–	–	–	–	–	–	Negative	–	–	–
JMC30	CPE	Wild boar	*Sus scrofa*	**Positive**	28.8	Negative	–	–	–	Negative	–	–	–	Negative	–	–	–	Negative	–	–	–	Unknown	Negative	–	–	–	–	–	–	–	–	Negative	–	–	–
JMC31	CPE	Wild boar	*Sus scrofa*	Negative	–	–	–	–	–	–	–	–	–	–	–	–	–	–	–	–	–	–	Negative	–	–	–	–	–	–	–	–	Negative	–	–	–
JMC32	CPE	Wild boar	*Sus scrofa*	Negative	–	–	–	–	–	–	–	–	–	–	–	–	–	–	–	–	–	–	Negative	–	–	–	–	–	–	–	–	Negative	–	–	–
JMC33	CPE	Wild boar	*Sus scrofa*	Negative	–	–	–	–	–	–	–	–	–	–	–	–	–	–	–	–	–	–	Negative	–	–	–	–	–	–	–	–	Negative	–	–	–
RGUA1	CPE	Red fox	*Vulpes vulpes*	**Positive**	36.9	Not done	–	–	–	Not done	–	–	–	Not done	–	–	–	Not done	–	–	–	Unknown	Negative	–	–	–	–	–	–	–	–	Not done	–	–	–
RGUA2	CPE	Red fox	*Vulpes vulpes*	Negative	–	–	–	–	–	–	–	–	–	–	–	–	–	–	–	–	–	–	Negative	–	–	–	–	–	–	–	–	Not done	–	–	–
CGUA1	CPE	Roe deer	*Capreolus capreolus*	Negative	–	–	–	–	–	–	–	–	–	–	–	–	–	–	–	–	–	–	Negative	–	–	–	–	–	–	–	–	Negative	–	–	–
FGUA1	CPE	Stone marten	*Martes foina*	Negative	–	–	–	–	–	–	–	–	–	–	–	–	–	–	–	–	–	–	Negative	–	–	–	–	–	–	–	–	Not done	–	–	–
CGUA2	CPE	Roe deer	*Capreolus capreolus*	Negative	–	–	–	–	–	–	–	–	–	–	–	–	–	–	–	–	–	–	Negative	Unspecific	–	–	–	–	–	–	–	Negative	–	–	–
JGUA1	CPE	Wild boar	*Sus scrofa*	Negative	–	–	–	–	–	–	–	–	–	–	–	–	–	–	–	–	–	–	Negative	–	–	–	–	–	–	–	–	Negative	–	–	–
CGUA3	CPE	Roe deer	*Capreolus capreolus*	Negative	–	–	–	–	–	–	–	–	–	–	–	–	–	–	–	–	–	–	Negative	–	–	–	–	–	–	–	–	Negative	–	–	–
JGUA2	CPE	Wild boar	*Sus scrofa*	Negative	–	–	–	–	–	–	–	–	–	–	–	–	–	–	–	–	–	–	Negative	–	–	–	–	–	–	–	–	Negative	–	–	–
JGUA3	CPE	Wild boar	*Sus scrofa*	Negative	–	–	–	–	–	–	–	–	–	–	–	–	–	–	–	–	–	–	Negative	*Stichotrichia*	–	–	–	–	–	–	–	Negative	–	–	–
JGUA4	CPE	Wild boar	*Sus scrofa*	Negative	–	–	–	–	–	–	–	–	–	–	–	–	–	–	–	–	–	–	Negative	–	–	–	–	–	–	–	–	Negative	–	–	–
JGUA5	CPE	Wild boar	*Sus scrofa*	Negative	–	–	–	–	–	–	–	–	–	–	–	–	–	–	–	–	–	–	Negative	–	–	–	–	–	–	–	–	Negative	–	–	–

CGUA4	CPE	Roe deer	*Capreolus capreolus*	Negative	–	–	–	–	–	–	–	–	–	–	–	–	–	–	–	–	–	–	Negative	*Hortelaria*	–	–	–	–	–	–	–	Negative	–	–	–
CGUA5	CPE	Roe deer	*Capreolus capreolus*	Negative	–	–	–	–	–	–	–	–	–	–	–	–	–	–	–	–	–	–	Negative	Unspecific	–	–	–	–	–	–	–	Negative	–	–	–
CAGUA1	CPE	Goat	*Capra hircus*	Negative	–	–	–	–	–	–	–	–	–	–	–	–	–	–	–	–	–	–	Negative	–	–	–	–	–	–	–	–	Negative	–	–	–
CAGUA2	CPE	Goat	*Capra hircus*	Negative	–	–	–	–	–	–	–	–	–	–	–	–	–	–	–	–	–	–	Negative	–	–	–	–	–	–	–	–	Negative	–	–	–
OVGUA1	CPE	Sheep	*Ovis aries*	Negative	–	–	–	–	–	–	–	–	–	–	–	–	–	–	–	–	–	–	Negative	–	–	–	–	–	–	–	–	Negative	–	–	–
RGUA3	CPE	Red fox	*Vulpes vulpes*	**Positive**	35.5	Not done	–	–	–	Not done	–	–	–	Not done	–	–	–	Not done	–	–	–	Unknown	Negative	–	–	–	–	–	–	–	–	Not done	–	–	–
RGUA4	CPE	Red fox	*Vulpes vulpes*	**Positive**	38.9	Not done	–	–	–	Not done	–	–	–	Not done	–	–	–	Not done	–	–	–	Unknown	Negative	Unspecific	–	–	–	–	–	–	–	Not done	–	–	–
RGUA5	CPE	Red fox	*Vulpes vulpes*	Negative	–	–	–	–	–	–	–	–	–	–	–	–	–	–	–	–	–	–	Negative	Uncultured eukaryote	–	–	–	–	–	–	–	Not done	–	–	–
FGUA3	CPE	Stone marten	*Martes foina*	**Positive**	35.8	Not done	–	–	–	Not done	–	–	–	Not done	–	–	–	Not done	–	–	–	Unknown	Negative	–	–	–	–	–	–	–	–	Not done	–	–	–
FGUA4	CPE	Stone marten	*Martes foina*	**Positive**	39.2	Not done	–	–	–	Not done	–	–	–	Not done	–	–	–	Not done	–	–	–	Unknown	Negative	–	–	–	–	–	–	–	–	Not done	–	–	–
RGUA6	CPE	Red fox	*Vulpes vulpes*	Negative	–	–	–	–	–	–	–	–	–	–	–	–	–	–	–	–	–	–	Negative	–	–	–	–	–	–	–	–	Not done	–	–	–
OVGUA2	CPE	Sheep	*Ovis aries*	Negative	–	–	–	–	–	–	–	–	–	–	–	–	–	–	–	–	–	–	Negative	–	–	–	–	–	–	–	–	Negative	–	–	–
FGUA5	CPE	Stone marten	*Martes foina*	Negative	–	–	–	–	–	–	–	–	–	–	–	–	–	–	–	–	–	–	Negative	–	–	–	–	–	–	–	–	Not done	–	–	–
OVGUA3	CPE	Sheep	*Ovis aries*	Negative	–	–	–	–	–	–	–	–	–	–	–	–	–	–	–	–	–	–	Negative	–	–	–	–	–	–	–	–	Negative	–	–	–
JGUA6	CPE	Wild boar	*Sus scrofa*	Negative	–	–	–	–	–	–	–	–	–	–	–	–	–	–	–	–	–	–	Negative	–	–	–	–	–	–	–	–	Negative	–	–	–
RGUA7	CPE	Red fox	*Vulpes vulpes*	Negative	–	–	–	–	–	–	–	–	–	–	–	–	–	–	–	–	–	–	Negative	–	–	–	–	–	–	–	–	Not done	–	–	–
RGUA8	CPE	Red fox	*Vulpes vulpes*	Negative	–	–	–	–	–	–	–	–	–	–	–	–	–	–	–	–	–	–	Negative	–	–	–	–	–	–	–	–	Not done	–	–	–
VACOA1	FBR	Cattle	*Bos taurus*	Negative	–	–	–	–	–	–	–	–	–	–	–	–	–	–	–	–	–	–	Negative	–	–	–	–	–	–	–	–	Negative	–	–	–
VACOA2	FBR	Cattle	*Bos taurus*	Negative	–	–	–	–	–	–	–	–	–	–	–	–	–	–	–	–	–	–	Negative	–	–	–	–	–	–	–	–	Negative	–	–	–
VACOA3	FBR	Cattle	*Bos taurus*	Negative	–	–	–	–	–	–	–	–	–	–	–	–	–	–	–	–	–	–	Negative	–	–	–	–	–	–	–	–	Negative	–	–	–
VACOA4	FBR	Cattle	*Bos taurus*	Negative	–	–	–	–	–	–	–	–	–	–	–	–	–	–	–	–	–	–	Negative	–	–	–	–	–	–	–	–	Negative	–	–	–
VACOA5	FBR	Cattle	*Bos taurus*	Negative	–	–	–	–	–	–	–	–	–	–	–	–	–	–	–	–	–	–	Negative	–	–	–	–	–	–	–	–	Negative	–	–	–
VACOA6	FBR	Cattle	*Bos taurus*	Negative	–	–	–	–	–	–	–	–	–	–	–	–	–	–	–	–	–	–	Negative	–	–	–	–	–	–	–	–	Negative	–	–	–
VACOA7	FBR	Cattle	*Bos taurus*	**Positive**	36.7	Not done	–	–	–	Not done	–	–	–	Not done	–	–	–	Not done	–	–	–	Unknown	Negative	–	–	–	–	–	–	–	–	Negative	–	–	–
VACOA8	FBR	Cattle	*Bos taurus*	Negative	–	–	–	–	–	–	–	–	–	–	–	–	–	–	–	–	–	–	Negative	–	–	–	–	–	–	–	–	Negative	–	–	–
VACOA9	FBR	Cattle	*Bos taurus*	Negative	–	–	–	–	–	–	–	–	–	–	–	–	–	–	–	–	–	–	Negative	–	–	–	–	–	–	–	–	Negative	–	–	–
VACOA10	FBR	Cattle	*Bos taurus*	Negative	–	–	–	–	–	–	–	–	–	–	–	–	–	–	–	–	–	–	Negative	–	–	–	–	–	–	–	–	Negative	–	–	–
VACOA11	FBR	Cattle	*Bos taurus*	Negative	–	–	–	–	–	–	–	–	–	–	–	–	–	–	–	–	–	–	Negative	–	–	–	–	–	–	–	–	Negative	–	–	–
CAVCOA1	FBR	Horse	*Equus caballus*	Negative	–	–	–	–	–	–	–	–	–	–	–	–	–	–	–	–	–	–	Negative	–	–	–	–	–	–	–	–	Negative	–	–	–
CAVCOA2	FBR	Horse	*Equus caballus*	Negative	–	–	–	–	–	–	–	–	–	–	–	–	–	–	–	–	–	–	Negative	–	–	–	–	–	–	–	–	Negative	–	–	–
CAVCOA3	FBR	Horse	*Equus caballus*	Negative	–	–	–	–	–	–	–	–	–	–	–	–	–	–	–	–	–	–	Negative	–	–	–	–	–	–	–	–	Negative	–	–	–
CAVCOA4	FBR	Horse	*Equus caballus*	Negative	–	–	–	–	–	–	–	–	–	–	–	–	–	–	–	–	–	–	Negative	–	–	–	–	–	–	–	–	Negative	–	–	–
VACOA12	FBR	Cattle	*Bos taurus*	Negative	–	–	–	–	–	–	–	–	–	–	–	–	–	–	–	–	–	–	Negative	–	–	–	–	–	–	–	–	Negative	–	–	–
VACOA13	FBR	Cattle	*Bos taurus*	**Positive**	35.7	Not done	–	–	–	Not done	–	–	–	Not done	–	–	–	Not done	–	–	–	Unknown	Negative	–	–	–	–	–	–	–	–	Negative	–	–	–
VACOA14	FBR	Cattle	*Bos taurus*	**Positive**	37.4	Not done	–	–	–	Not done	–	–	–	Not done	–	–	–	Not done	–	–	–	Unknown	Negative	–	–	–	–	–	–	–	–	Negative	–	–	–
VACOA15	FBR	Cattle	*Bos taurus*	Negative	–	–	–	–	–	–	–	–	–	–	–	–	–	–	–	–	–	–	Negative	–	–	–	–	–	–	–	–	Negative	–	–	–
VACOA16	FBR	Cattle	*Bos taurus*	Negative	–	–	–	–	–	–	–	–	–	–	–	–	–	–	–	–	–	–	Negative	–	–	–	–	–	–	–	–	Negative	–	–	–
VACOA17	FBR	Cattle	*Bos taurus*	Negative	–	–	–	–	–	–	–	–	–	–	–	–	–	–	–	–	–	–	Negative	–	–	–	–	–	–	–	–	Negative	–	–	–
VACOA18	FBR	Cattle	*Bos taurus*	Negative	–	–	–	–	–	–	–	–	–	–	–	–	–	–	–	–	–	–	Negative	–	–	–	–	–	–	–	–	Negative	–	–	–
VACOA19	FBR	Cattle	*Bos taurus*	Negative	–	–	–	–	–	–	–	–	–	–	–	–	–	–	–	–	–	–	Negative	–	–	–	–	–	–	–	–	Negative	–	–	–
VACOA20	FBR	Cattle	*Bos taurus*	Negative	–	–	–	–	–	–	–	–	–	–	–	–	–	–	–	–	–	–	Negative	–	–	–	–	–	–	–	–	Negative	–	–	–
VACOA21	FBR	Cattle	*Bos taurus*	Negative	–	–	–	–	–	–	–	–	–	–	–	–	–	–	–	–	–	–	Negative	–	–	–	–	–	–	–	–	Negative	–	–	–
CAVCOA6	FBR	Horse	*Equus caballus*	**Positive**	30	Not done	–	–	–	Not done	–	–	–	Not done	–	–	–	Not done	–	–	–	Unknown	Negative	–	–	–	–	–	–	–	–	Negative	–	–	–
CAVCOA7	FBR	Horse	*Equus caballus*	**Positive**	29.5	**Positive**	**E**	None	Yes	Not done	–	–	–	Not done	–	–	–	Not done	–	–	–	**E**	Negative	–	–	–	–	–	–	–	–	Negative	–	–	–
CAVCOA8	FBR	Horse	*Equus caballus*	Negative	–	–	–	–	–	–	–	–	–	–	–	–	–	–	–	–	–	–	Negative	–	–	–	–	–	–	–	–	Negative	–	–	–
CAVCOA9	FBR	Horse	*Equus caballus*	Negative	–	–	–	–	–	–	–	–	–	–	–	–	–	–	–	–	–	–	Negative	–	–	–	–	–	–	–	–	Negative	–	–	–
CAVCOA10	FBR	Horse	*Equus caballus*	Negative	–	–	–	–	–	–	–	–	–	–	–	–	–	–	–	–	–	–	Negative	–	–	–	–	–	–	–	–	Negative	–	–	–
CAVCOA11	FBR	Horse	*Equus caballus*	Negative	–	–	–	–	–	–	–	–	–	–	–	–	–	–	–	–	–	–	Negative	–	–	–	–	–	–	–	–	Negative	–	–	–
CAVCOA12	FBR	Horse	*Equus caballus*	Negative	–	–	–	–	–	–	–	–	–	–	–	–	–	–	–	–	–	–	Negative	–	–	–	–	–	–	–	–	Negative	–	–	–
CAVCOA13	FBR	Horse	*Equus caballus*	Negative	–	–	–	–	–	–	–	–	–	–	–	–	–	–	–	–	–	–	Negative	–	–	–	–	–	–	–	–	Negative	–	–	–
CAVCOA14	FBR	Horse	*Equus caballus*	Negative	–	–	–	–	–	–	–	–	–	–	–	–	–	–	–	–	–	–	Negative	–	–	–	–	–	–	–	–	Negative	–	–	–
CAVCOA15	FBR	Horse	*Equus caballus*	Negative	–	–	–	–	–	–	–	–	–	–	–	–	–	–	–	–	–	–	Negative	–	–	–	–	–	–	–	–	Negative	–	–	–
CAVCOA16	FBR	Horse	*Equus caballus*	Negative	–	–	–	–	–	–	–	–	–	–	–	–	–	–	–	–	–	–	Negative	–	–	–	–	–	–	–	–	Negative	–	–	–
CAVCOA17	FBR	Horse	*Equus caballus*	Negative	–	–	–	–	–	–	–	–	–	–	–	–	–	–	–	–	–	–	Negative	Uncultured eukaryote	–	–	–	–	–	–	–	Negative	–	–	–
CAVCOA18	FBR	Horse	*Equus caballus*	Negative	–	–	–	–	–	–	–	–	–	–	–	–	–	–	–	–	–	–	Negative	–	–	–	–	–	–	–	–	Negative	–	–	–
CAVCOA19	FBR	Horse	*Equus caballus*	Negative	–	–	–	–	–	–	–	–	–	–	–	–	–	–	–	–	–	–	Negative	–	–	–	–	–	–	–	–	Negative	–	–	–
CAVCOA20	FBR	Horse	*Equus caballus*	Negative	–	–	–	–	–	–	–	–	–	–	–	–	–	–	–	–	–	–	Negative	–	–	–	–	–	–	–	–	Negative	–	–	–
CAVCOA21	FBR	Horse	*Equus caballus*	Negative	–	–	–	–	–	–	–	–	–	–	–	–	–	–	–	–	–	–	Negative	–	–	–	–	–	–	–	–	Negative	–	–	–
VACOA24	FBR	Cattle	*Bos taurus*	Negative	–	–	–	–	–	–	–	–	–	–	–	–	–	–	–	–	–	–	Negative	–	–	–	–	–	–	–	–	Negative	–	–	–
VACOA25	FBR	Cattle	*Bos taurus*	Negative	–	–	–	–	–	–	–	–	–	–	–	–	–	–	–	–	–	–	Negative	–	–	–	–	–	–	–	–	Negative	–	–	–
VACOA26	FBR	Cattle	*Bos taurus*	Negative	–	–	–	–	–	–	–	–	–	–	–	–	–	–	–	–	–	–	Negative	–	–	–	–	–	–	–	–	Negative	–	–	–
VACOA27	FBR	Cattle	*Bos taurus*	Negative	–	–	–	–	–	–	–	–	–	–	–	–	–	–	–	–	–	–	Negative	–	–	–	–	–	–	–	–	Negative	–	–	–
VACOA28	FBR	Cattle	*Bos taurus*	Negative	–	–	–	–	–	–	–	–	–	–	–	–	–	–	–	–	–	–	Negative	–	–	–	–	–	–	–	–	Negative	–	–	–
VACOA29	FBR	Cattle	*Bos taurus*	Negative	–	–	–	–	–	–	–	–	–	–	–	–	–	–	–	–	–	–	Negative	–	–	–	–	–	–	–	–	Negative	–	–	–
VACOA30	FBR	Cattle	*Bos taurus*	Negative	–	–	–	–	–	–	–	–	–	–	–	–	–	–	–	–	–	–	Negative	–	–	–	–	–	–	–	–	Negative	–	–	–
VACOA31	FBR	Cattle	*Bos taurus*	Negative	–	–	–	–	–	–	–	–	–	–	–	–	–	–	–	–	–	–	Negative	–	–	–	–	–	–	–	–	Negative	–	–	–
VACOA32	FBR	Cattle	*Bos taurus*	Negative	–	–	–	–	–	–	–	–	–	–	–	–	–	–	–	–	–	–	Negative	–	–	–	–	–	–	–	–	Negative	–	–	–
VACOA33	FBR	Cattle	*Bos taurus*	Negative	–	–	–	–	–	–	–	–	–	–	–	–	–	–	–	–	–	–	Negative	–	–	–	–	–	–	–	–	Negative	–	–	–
VACOA34	FBR	Cattle	*Bos taurus*	Negative	–	–	–	–	–	–	–	–	–	–	–	–	–	–	–	–	–	–	Negative	–	–	–	–	–	–	–	–	Negative	–	–	–
VACOA35	FBR	Cattle	*Bos taurus*	Negative	–	–	–	–	–	–	–	–	–	–	–	–	–	–	–	–	–	–	Negative	–	–	–	–	–	–	–	–	Negative	–	–	–
VACOA36	FBR	Cattle	*Bos taurus*	Negative	–	–	–	–	–	–	–	–	–	–	–	–	–	–	–	–	–	–	Negative	–	–	–	–	–	–	–	–	Negative	–	–	–
CAVCOA22	FBR	Horse	*Equus caballus*	Negative	–	–	–	–	–	–	–	–	–	–	–	–	–	–	–	–	–	–	Negative	–	–	–	–	–	–	–	–	Negative	–	–	–
CAVCOA23	FBR	Horse	*Equus caballus*	Negative	–	–	–	–	–	–	–	–	–	–	–	–	–	–	–	–	–	–	Negative	–	–	–	–	–	–	–	–	Negative	–	–	–
CAVCOA24	FBR	Horse	*Equus caballus*	Negative	–	–	–	–	–	–	–	–	–	–	–	–	–	–	–	–	–	–	Negative	–	–	–	–	–	–	–	–	Negative	–	–	–
CAVCOA25	FBR	Horse	*Equus caballus*	Negative	–	–	–	–	–	–	–	–	–	–	–	–	–	–	–	–	–	–	Negative	–	–	–	–	–	–	–	–	Negative	–	–	–
CAVCOA26	FBR	Horse	*Equus caballus*	Negative	–	–	–	–	–	–	–	–	–	–	–	–	–	–	–	–	–	–	Negative	–	–	–	–	–	–	–	–	Negative	–	–	–
CAVCOA27	FBR	Horse	*Equus caballus*	Negative	–	–	–	–	–	–	–	–	–	–	–	–	–	–	–	–	–	–	Negative	–	–	–	–	–	–	–	–	Negative	–	–	–
RLO1	LM	Red fox	*Vulpes vulpes*	Negative	–	–	–	–	–	–	–	–	–	–	–	–	–	–	–	–	–	–	Negative	–	–	–	–	–	–	–	–	Not done	–	–	–
RLO2	LM	Red fox	*Vulpes vulpes*	Negative	–	–	–	–	–	–	–	–	–	–	–	–	–	–	–	–	–	–	Negative	–	–	–	–	–	–	–	–	Not done	–	–	–
RLO3	LM	Red fox	*Vulpes vulpes*	Negative	–	–	–	–	–	–	–	–	–	–	–	–	–	–	–	–	–	–	**Positive**	***C. canis***	686InsTG, T739A	Yes	Untypable	–	–	–	***C. canis***	Not done	–	–	–
RLO4	LM	Red fox	*Vulpes vulpes*	Negative	–	–	–	–	–	–	–	–	–	–	–	–	–	–	–	–	–	–	Negative	–	–	–	–	–	–	–	–	Not done	–	–	–
RLO5	LM	Red fox	*Vulpes vulpes*	Negative	–	–	–	–	–	–	–	–	–	–	–	–	–	–	–	–	–	–	Negative	–	–	–	–	–	–	–	–	Not done	–	–	–
VLOM1	LM	Red deer	*Cervus elaphus*	Negative	–	–	–	–	–	–	–	–	–	–	–	–	–	–	–	–	–	–	Negative	–	–	–	–	–	–	–	–	Negative	–	–	–
VLOM2	LM	Red deer	*Cervus elaphus*	Negative	–	–	–	–	–	–	–	–	–	–	–	–	–	–	–	–	–	–	Negative	–	–	–	–	–	–	–	–	Negative	–	–	–
VLOM3	LM	Red deer	*Cervus elaphus*	Negative	–	–	–	–	–	–	–	–	–	–	–	–	–	–	–	–	–	–	**Positive**	***C. occultus***	484InsAT	Yes	Untypable	–	–	–	***C. occultus***	Negative	–	–	–
VLOM4	LM	Red deer	*Cervus elaphus*	Negative	–	–	–	–	–	–	–	–	–	–	–	–	–	–	–	–	–	–	Negative	–	–	–	–	–	–	–	–	Negative	–	–	–
VLOM5	LM	Red deer	*Cervus elaphus*	Negative	–	–	–	–	–	–	–	–	–	–	–	–	–	–	–	–	–	–	Negative	–	–	–	–	–	–	–	–	Negative	–	–	–
VLOM6	LM	Red deer	*Cervus elaphus*	Negative	–	–	–	–	–	–	–	–	–	–	–	–	–	–	–	–	–	–	Negative	–	–	–	–	–	–	–	–	Negative	–	–	–
VLOM7	LM	Red deer	*Cervus elaphus*	Negative	–	–	–	–	–	–	–	–	–	–	–	–	–	–	–	–	–	–	Negative	–	–	–	–	–	–	–	–	Negative	–	–	–
VLOM8	LM	Red deer	*Cervus elaphus*	Negative	–	–	–	–	–	–	–	–	–	–	–	–	–	–	–	–	–	–	Negative	–	–	–	–	–	–	–	–	Negative	–	–	–
VLOM9	LM	Red deer	*Cervus elaphus*	Negative	–	–	–	–	–	–	–	–	–	–	–	–	–	–	–	–	–	–	Negative	–	–	–	–	–	–	–	–	Negative	–	–	–
VLOM10	LM	Red deer	*Cervus elaphus*	Negative	–	–	–	–	–	–	–	–	–	–	–	–	–	–	–	–	–	–	Negative	–	–	–	–	–	–	–	–	Negative	–	–	–
VLO1	LM	Red deer	*Cervus elaphus*	Negative	–	–	–	–	–	–	–	–	–	–	–	–	–	–	–	–	–	–	Negative	–	–	–	–	–	–	–	–	Negative	–	–	–
VLO2	LM	Red deer	*Cervus elaphus*	Negative	–	–	–	–	–	–	–	–	–	–	–	–	–	–	–	–	–	–	Negative	–	–	–	–	–	–	–	–	Negative	–	–	–
VLO3	LM	Red deer	*Cervus elaphus*	Negative	–	–	–	–	–	–	–	–	–	–	–	–	–	–	–	–	–	–	Negative	–	–	–	–	–	–	–	–	Negative	–	–	–
VLO4	LM	Red deer	*Cervus elaphus*	Negative	–	–	–	–	–	–	–	–	–	–	–	–	–	–	–	–	–	–	Negative	–	–	–	–	–	–	–	–	Negative	–	–	–
VLO5	LM	Red deer	*Cervus elaphus*	Negative	–	–	–	–	–	–	–	–	–	–	–	–	–	–	–	–	–	–	Negative	–	–	–	–	–	–	–	–	Negative	–	–	–
VLO6	LM	Red deer	*Cervus elaphus*	Negative	–	–	–	–	–	–	–	–	–	–	–	–	–	–	–	–	–	–	Negative	*Sterkiella* sp.	–	–	–	–	–	–	–	Negative	–	–	–
VLO7	LM	Red deer	*Cervus elaphus*	Negative	–	–	–	–	–	–	–	–	–	–	–	–	–	–	–	–	–	–	Negative	–	–	–	–	–	–	–	–	Negative	–	–	–
VLO8	LM	Red deer	*Cervus elaphus*	Negative	–	–	–	–	–	–	–	–	–	–	–	–	–	–	–	–	–	–	Negative	–	–	–	–	–	–	–	–	Negative	–	–	–
VLO9	LM	Red deer	*Cervus elaphus*	Negative	–	–	–	–	–	–	–	–	–	–	–	–	–	–	–	–	–	–	Negative	–	–	–	–	–	–	–	–	Negative	–	–	–
VLO10	LM	Red deer	*Cervus elaphus*	Negative	–	–	–	–	–	–	–	–	–	–	–	–	–	–	–	–	–	–	Negative	–	–	–	–	–	–	–	–	Negative	–	–	–
VLO11	LM	Red deer	*Cervus elaphus*	Negative	–	–	–	–	–	–	–	–	–	–	–	–	–	–	–	–	–	–	Negative	–	–	–	–	–	–	–	–	Negative	–	–	–
VLO12	LM	Red deer	*Cervus elaphus*	Negative	–	–	–	–	–	–	–	–	–	–	–	–	–	–	–	–	–	–	Negative	–	–	–	–	–	–	–	–	Negative	–	–	–
VLO13	LM	Red deer	*Cervus elaphus*	Negative	–	–	–	–	–	–	–	–	–	–	–	–	–	–	–	–	–	–	Negative	–	–	–	–	–	–	–	–	Negative	–	–	–
VLO14	LM	Red deer	*Cervus elaphus*	Negative	–	–	–	–	–	–	–	–	–	–	–	–	–	–	–	–	–	–	Negative	–	–	–	–	–	–	–	–	Negative	–	–	–
VLO15	LM	Red deer	*Cervus elaphus*	Negative	–	–	–	–	–	–	–	–	–	–	–	–	–	–	–	–	–	–	Negative	–	–	–	–	–	–	–	–	Negative	–	–	–
VLO16	LM	Red deer	*Cervus elaphus*	Negative	–	–	–	–	–	–	–	–	–	–	–	–	–	–	–	–	–	–	Negative	–	–	–	–	–	–	–	–	Negative	–	–	–
VLO17	LM	Red deer	*Cervus elaphus*	Negative	–	–	–	–	–	–	–	–	–	–	–	–	–	–	–	–	–	–	Negative	–	–	–	–	–	–	–	–	Negative	–	–	–
VLO18	LM	Red deer	*Cervus elaphus*	Negative	–	–	–	–	–	–	–	–	–	–	–	–	–	–	–	–	–	–	Negative	–	–	–	–	–	–	–	–	Negative	–	–	–
VLO19	LM	Red deer	*Cervus elaphus*	Negative	–	–	–	–	–	–	–	–	–	–	–	–	–	–	–	–	–	–	Negative	*Urosoma emarginata*	–	–	–	–	–	–	–	Negative	–	–	–

VLO20	LM	Red deer	*Cervus elaphus*	Negative	–	–	–	–	–	–	–	–	–	–	–	–	–	–	–	–	–	–	Negative	–	–	–	–	–	–	–	–	Negative	–	–	–
VLO21	LM	Red deer	*Cervus elaphus*	Negative	–	–	–	–	–	–	–	–	–	–	–	–	–	–	–	–	–	–	Negative	–	–	–	–	–	–	–	–	Negative	–	–	–
VLO22	LM	Red deer	*Cervus elaphus*	Negative	–	–	–	–	–	–	–	–	–	–	–	–	–	–	–	–	–	–	Negative	–	–	–	–	–	–	–	–	Negative	–	–	–
VLO23	LM	Red deer	*Cervus elaphus*	Negative	–	–	–	–	–	–	–	–	–	–	–	–	–	–	–	–	–	–	Negative	*Rigidohymena inquieta*	–	–	–	–	–	–	–	Negative	–	–	–
VLO24	LM	Red deer	*Cervus elaphus*	Negative	–	–	–	–	–	–	–	–	–	–	–	–	–	–	–	–	–	–	Negative	–	–	–	–	–	–	–	–	Negative	–	–	–
VLO25	LM	Red deer	*Cervus elaphus*	Negative	–	–	–	–	–	–	–	–	–	–	–	–	–	–	–	–	–	–	Negative	–	–	–	–	–	–	–	–	Negative	–	–	–
VLO26	LM	Red deer	*Cervus elaphus*	Negative	–	–	–	–	–	–	–	–	–	–	–	–	–	–	–	–	–	–	Negative	–	–	–	–	–	–	–	–	Negative	–	–	–
VLO27	LM	Red deer	*Cervus elaphus*	Negative	–	–	–	–	–	–	–	–	–	–	–	–	–	–	–	–	–	–	Negative	–	–	–	–	–	–	–	–	Negative	–	–	–
VLO28	LM	Red deer	*Cervus elaphus*	Negative	–	–	–	–	–	–	–	–	–	–	–	–	–	–	–	–	–	–	Negative	–	–	–	–	–	–	–	–	Negative	–	–	–
VLO29	LM	Red deer	*Cervus elaphus*	Negative	–	–	–	–	–	–	–	–	–	–	–	–	–	–	–	–	–	–	Negative	Uncultured eukaryote	–	–	–	–	–	–	–	Negative	–	–	–
VLO30	LM	Red deer	*Cervus elaphus*	Negative	–	–	–	–	–	–	–	–	–	–	–	–	–	–	–	–	–	–	Negative	–	–	–	–	–	–	–	–	Negative	–	–	–
VLO31	LM	Red deer	*Cervus elaphus*	Negative	–	–	–	–	–	–	–	–	–	–	–	–	–	–	–	–	–	–	Negative	–	–	–	–	–	–	–	–	Negative	–	–	–
VLO32	LM	Red deer	*Cervus elaphus*	Negative	–	–	–	–	–	–	–	–	–	–	–	–	–	–	–	–	–	–	Negative	–	–	–	–	–	–	–	–	Negative	–	–	–
VLO33	LM	Red deer	*Cervus elaphus*	Negative	–	–	–	–	–	–	–	–	–	–	–	–	–	–	–	–	–	–	Negative	–	–	–	–	–	–	–	–	Negative	–	–	–
VLO34	LM	Red deer	*Cervus elaphus*	Negative	–	–	–	–	–	–	–	–	–	–	–	–	–	–	–	–	–	–	Negative	–	–	–	–	–	–	–	–	Negative	–	–	–
VLO35	LM	Red deer	*Cervus elaphus*	Negative	–	–	–	–	–	–	–	–	–	–	–	–	–	–	–	–	–	–	Negative	–	–	–	–	–	–	–	–	Negative	–	–	–
JLO1	LM	Wild boar	*Sus scrofa*	Negative	–	–	–	–	–	–	–	–	–	–	–	–	–	–	–	–	–	–	Negative	–	–	–	–	–	–	–	–	Negative	–	–	–
CLO1	LM	Roe deer	*Capreolus capreolus*	Negative	–	–	–	–	–	–	–	–	–	–	–	–	–	–	–	–	–	–	Negative	–	–	–	–	–	–	–	–	Negative	–	–	–
CLO2	LM	Roe deer	*Capreolus capreolus*	Negative	–	–	–	–	–	–	–	–	–	–	–	–	–	–	–	–	–	–	Negative	–	–	–	–	–	–	–	–	Negative	–	–	–
CLO3	LM	Roe deer	*Capreolus capreolus*	Negative	–	–	–	–	–	–	–	–	–	–	–	–	–	–	–	–	–	–	Negative	–	–	–	–	–	–	–	–	Negative	–	–	–
RLO6	LM	Red fox	*Vulpes vulpes*	Negative	–	–	–	–	–	–	–	–	–	–	–	–	–	–	–	–	–	–	Negative	Uncultured eukaryote	–	–	–	–	–	–	–	Not done	–	–	–
RLO7	LM	Red fox	*Vulpes vulpes*	Negative	–	–	–	–	–	–	–	–	–	–	–	–	–	–	–	–	–	–	Negative	–	–	–	–	–	–	–	–	Not done	–	–	–
RLO8	LM	Red fox	*Vulpes vulpes*	Negative	–	–	–	–	–	–	–	–	–	–	–	–	–	–	–	–	–	–	Negative	–	–	–	–	–	–	–	–	Not done	–	–	–
CLO4	LM	Roe deer	*Capreolus capreolus*	Negative	–	–	–	–	–	–	–	–	–	–	–	–	–	–	–	–	–	–	Negative	–	–	–	–	–	–	–	–	Negative	–	–	–
CLO5	LM	Roe deer	*Capreolus capreolus*	Negative	–	–	–	–	–	–	–	–	–	–	–	–	–	–	–	–	–	–	Negative	–	–	–	–	–	–	–	–	Negative	–	–	–
VLO36	LM	Red deer	*Cervus elaphus*	Negative	–	–	–	–	–	–	–	–	–	–	–	–	–	–	–	–	–	–	Negative	–	–	–	–	–	–	–	–	Negative	–	–	–
VLO37	LM	Red deer	*Cervus elaphus*	Negative	–	–	–	–	–	–	–	–	–	–	–	–	–	–	–	–	–	–	Negative	–	–	–	–	–	–	–	–	Negative	–	–	–
RMAL1	MNR	Red fox	*Vulpes vulpes*	Negative	–	–	–	–	–	–	–	–	–	–	–	–	–	–	–	–	–	–	Negative	–	–	–	–	–	–	–	–	Not done	–	–	–
RMAL2	MNR	Red fox	*Vulpes vulpes*	Negative	–	–	–	–	–	–	–	–	–	–	–	–	–	–	–	–	–	–	Negative	–	–	–	–	–	–	–	–	Not done	–	–	–
RMAL3	MNR	Red fox	*Vulpes vulpes*	Negative	–	–	–	–	–	–	–	–	–	–	–	–	–	–	–	–	–	–	Negative	–	–	–	–	–	–	–	–	Not done	–	–	–
RMAL4	MNR	Red fox	*Vulpes vulpes*	Negative	–	–	–	–	–	–	–	–	–	–	–	–	–	–	–	–	–	–	Negative	–	–	–	–	–	–	–	–	Not done	–	–	–
FMAL1	MNR	Stone marten	*Martes foina*	Negative	–	–	–	–	–	–	–	–	–	–	–	–	–	–	–	–	–	–	Negative	–	–	–	–	–	–	–	–	Not done	–	–	–
RMAL5	MNR	Red fox	*Vulpes vulpes*	Negative	–	–	–	–	–	–	–	–	–	–	–	–	–	–	–	–	–	–	Negative	–	–	–	–	–	–	–	–	Not done	–	–	–
FMAL2	MNR	Stone marten	*Martes foina*	Negative	–	–	–	–	–	–	–	–	–	–	–	–	–	–	–	–	–	–	Negative	–	–	–	–	–	–	–	–	Not done	–	–	–
CMAL1	MNR	Roe deer	*Capreolus capreolus*	Negative	–	–	–	–	–	–	–	–	–	–	–	–	–	–	–	–	–	–	Negative	–	–	–	–	–	–	–	–	Negative	–	–	–
CMAL2	MNR	Roe deer	*Capreolus capreolus*	Negative	–	–	–	–	–	–	–	–	–	–	–	–	–	–	–	–	–	–	Negative	–	–	–	–	–	–	–	–	Negative	–	–	–
CMAL3	MNR	Roe deer	*Capreolus capreolus*	Negative	–	–	–	–	–	–	–	–	–	–	–	–	–	–	–	–	–	–	Negative	–	–	–	–	–	–	–	–	Negative	–	–	–
CMAL4	MNR	Roe deer	*Capreolus capreolus*	Negative	–	–	–	–	–	–	–	–	–	–	–	–	–	–	–	–	–	–	Negative	–	–	–	–	–	–	–	–	Negative	–	–	–
CMAL5	MNR	Roe deer	*Capreolus capreolus*	Negative	–	–	–	–	–	–	–	–	–	–	–	–	–	–	–	–	–	–	Negative	–	–	–	–	–	–	–	–	Negative	–	–	–
CMAL6	MNR	Roe deer	*Capreolus capreolus*	Negative	–	–	–	–	–	–	–	–	–	–	–	–	–	–	–	–	–	–	Negative	–	–	–	–	–	–	–	–	Negative	–	–	–
CMAL7	MNR	Roe deer	*Capreolus capreolus*	Negative	–	–	–	–	–	–	–	–	–	–	–	–	–	–	–	–	–	–	Negative	–	–	–	–	–	–	–	–	Negative	–	–	–
CMAL8	MNR	Roe deer	*Capreolus capreolus*	Negative	–	–	–	–	–	–	–	–	–	–	–	–	–	–	–	–	–	–	Negative	–	–	–	–	–	–	–	–	Negative	–	–	–
CMAL9	MNR	Roe deer	*Capreolus capreolus*	Negative	–	–	–	–	–	–	–	–	–	–	–	–	–	–	–	–	–	–	Negative	–	–	–	–	–	–	–	–	Negative	–	–	–
CMAL10	MNR	Roe deer	*Capreolus capreolus*	Negative	–	–	–	–	–	–	–	–	–	–	–	–	–	–	–	–	–	–	Negative	–	–	–	–	–	–	–	–	Negative	–	–	–
CMAL11	MNR	Roe deer	*Capreolus capreolus*	Negative	–	–	–	–	–	–	–	–	–	–	–	–	–	–	–	–	–	–	Negative	–	–	–	–	–	–	–	–	Negative	–	–	–
CMAL12	MNR	Roe deer	*Capreolus capreolus*	Negative	–	–	–	–	–	–	–	–	–	–	–	–	–	–	–	–	–	–	Negative	–	–	–	–	–	–	–	–	Negative	–	–	–
JMAL1	MNR	Wild boar	*Sus scrofa*	Negative	–	–	–	–	–	–	–	–	–	–	–	–	–	–	–	–	–	–	Negative	–	–	–	–	–	–	–	–	Negative	–	–	–
JMAL2	MNR	Wild boar	*Sus scrofa*	Negative	–	–	–	–	–	–	–	–	–	–	–	–	–	–	–	–	–	–	Negative	–	–	–	–	–	–	–	–	Negative	–	–	–
JMAL3	MNR	Wild boar	*Sus scrofa*	**Positive**	39.1	Not done	–	–	–	Not done	–	–	–	Not done	–	–	–	Not done	–	–	–	Unknown	Negative	–	–	–	–	–	–	–	–	Negative	–	–	–
JMAL4	MNR	Wild boar	*Sus scrofa*	Negative	–	–	–	–	–	–	–	–	–	–	–	–	–	–	–	–	–	–	Negative	–	–	–	–	–	–	–	–	Negative	–	–	–
FMAL3	MNR	Stone marten	*Martes foina*	Negative	–	–	–	–	–	–	–	–	–	–	–	–	–	–	–	–	–	–	Negative	–	–	–	–	–	–	–	–	Not done	–	–	–
JMAL5	MNR	Wild boar	*Sus scrofa*	Negative	–	–	–	–	–	–	–	–	–	–	–	–	–	–	–	–	–	–	Negative	–	–	–	–	–	–	–	–	Negative	–	–	–
JMAL6	MNR	Wild boar	*Sus scrofa*	Negative	–	–	–	–	–	–	–	–	–	–	–	–	–	–	–	–	–	–	Negative	–	–	–	–	–	–	–	–	Negative	–	–	–
JMAL7	MNR	Wild boar	*Sus scrofa*	Negative	–	–	–	–	–	–	–	–	–	–	–	–	–	–	–	–	–	–	Negative	–	–	–	–	–	–	–	–	Negative	–	–	–
JMAL8	MNR	Wild boar	*Sus scrofa*	**Positive**	34.9	Negative	–	–	–	Not done	–	–	–	Not done	–	–	–	Not done	–	–	–	Unknown	Negative	–	–	–	–	–	–	–	–	Negative	–	–	–
JMAL9	MNR	Wild boar	*Sus scrofa*	Negative	–	–	–	–	–	–	–	–	–	–	–	–	–	–	–	–	–	–	Negative	–	–	–	–	–	–	–	–	Negative	–	–	–
JMAL10	MNR	Wild boar	*Sus scrofa*	Negative	–	–	–	–	–	–	–	–	–	–	–	–	–	–	–	–	–	–	Negative	–	–	–	–	–	–	–	–	Negative	–	–	–
CMAL13	MNR	Roe deer	*Capreolus capreolus*	Negative	–	–	–	–	–	–	–	–	–	–	–	–	–	–	–	–	–	–	Negative	–	–	–	–	–	–	–	–	Negative	–	–	–
CMAL14	MNR	Roe deer	*Capreolus capreolus*	Negative	–	–	–	–	–	–	–	–	–	–	–	–	–	–	–	–	–	–	Negative	–	–	–	–	–	–	–	–	Negative	–	–	–
CMAL15	MNR	Roe deer	*Capreolus capreolus*	Negative	–	–	–	–	–	–	–	–	–	–	–	–	–	–	–	–	–	–	Negative	–	–	–	–	–	–	–	–	Negative	–	–	–
CMAL16	MNR	Roe deer	*Capreolus capreolus*	Negative	–	–	–	–	–	–	–	–	–	–	–	–	–	–	–	–	–	–	Negative	–	–	–	–	–	–	–	–	Negative	–	–	–
CMAL17	MNR	Roe deer	*Capreolus capreolus*	Negative	–	–	–	–	–	–	–	–	–	–	–	–	–	–	–	–	–	–	Negative	–	–	–	–	–	–	–	–	Negative	–	–	–
CMAL18	MNR	Roe deer	*Capreolus capreolus*	Negative	–	–	–	–	–	–	–	–	–	–	–	–	–	–	–	–	–	–	Negative	–	–	–	–	–	–	–	–	Negative	–	–	–
CMAL19	MNR	Roe deer	*Capreolus capreolus*	Negative	–	–	–	–	–	–	–	–	–	–	–	–	–	–	–	–	–	–	Negative	–	–	–	–	–	–	–	–	Negative	–	–	–
CMAL20	MNR	Roe deer	*Capreolus capreolus*	Negative	–	–	–	–	–	–	–	–	–	–	–	–	–	–	–	–	–	–	Negative	–	–	–	–	–	–	–	–	Negative	–	–	–
CMAL21	MNR	Roe deer	*Capreolus capreolus*	Negative	–	–	–	–	–	–	–	–	–	–	–	–	–	–	–	–	–	–	Negative	–	–	–	–	–	–	–	–	Negative	–	–	–
LIME1	GV	Iberian lynx	*Lynx pardinus*	**Positive**	31.3	Negative	–	–	–	Negative	–	–	–	Negative	–	–	–	Negative	–	–	–	Unknown	Negative	–	–	–	–	–	–	–	–	Not done	–	–	–
LIME2	GV	Iberian lynx	*Lynx pardinus*	**Positive**	34.4	Negative	–	–	–	Not done	–	–	–	Not done	–	–	–	Not done	–	–	–	Unknown	Negative	–	–	–	–	–	–	–	–	Not done	–	–	–
LIME3	GV	Iberian lynx	*Lynx pardinus*	**Positive**	39.8	Not done	–	–	–	Not done	–	–	–	Not done	–	–	–	Not done	–	–	–	Unknown	Negative	–	–	–	–	–	–	–	–	Not done	–	–	–
LIME4	GV	Iberian lynx	*Lynx pardinus*	Negative	–	–	–	–	–	–	–	–	–	–	–	–	–	–	–	–	–	–	Negative	–	–	–	–	–	–	–	–	Not done	–	–	–
LIME5	GV	Iberian lynx	*Lynx pardinus*	Negative	–	–	–	–	–	–	–	–	–	–	–	–	–	–	–	–	–	–	Negative	–	–	–	–	–	–	–	–	Not done	–	–	–
LIME6	GV	Iberian lynx	*Lynx pardinus*	Negative	–	–	–	–	–	–	–	–	–	–	–	–	–	–	–	–	–	–	Negative	Unspecific	–	–	–	–	–	–	–	Not done	–	–	–
LIME7	GV	Iberian lynx	*Lynx pardinus*	Negative	–	–	–	–	–	–	–	–	–	–	–	–	–	–	–	–	–	–	Negative	–	–	–	–	–	–	–	–	Not done	–	–	–
LIME8	GV	Iberian lynx	*Lynx pardinus*	**Positive**	37	Not done	–	–	–	Not done	–	–	–	Not done	–	–	–	Not done	–	–	–	Unknown	Negative	–	–	–	–	–	–	–	–	Not done	–	–	–
LIME9	GV	Iberian lynx	*Lynx pardinus*	Negative	–	–	–	–	–	–	–	–	–	–	–	–	–	–	–	–	–	–	Negative	Unspecific	–	–	–	–	–	–	–	Not done	–	–	–
LIME10	GV	Iberian lynx	*Lynx pardinus*	Negative	–	–	–	–	–	–	–	–	–	–	–	–	–	–	–	–	–	–	Negative	–	–	–	–	–	–	–	–	Not done	–	–	–
LIME11	GV	Iberian lynx	*Lynx pardinus*	Negative	–	–	–	–	–	–	–	–	–	–	–	–	–	–	–	–	–	–	Negative	–	–	–	–	–	–	–	–	Not done	–	–	–
LIME12	GV	Iberian lynx	*Lynx pardinus*	Negative	–	–	–	–	–	–	–	–	–	–	–	–	–	–	–	–	–	–	Negative	–	–	–	–	–	–	–	–	Not done	–	–	–
LIME13	GV	Iberian lynx	*Lynx pardinus*	Negative	–	–	–	–	–	–	–	–	–	–	–	–	–	–	–	–	–	–	Negative	–	–	–	–	–	–	–	–	Not done	–	–	–
LIME14	GV	Iberian lynx	*Lynx pardinus*	Negative	–	–	–	–	–	–	–	–	–	–	–	–	–	–	–	–	–	–	Negative	–	–	–	–	–	–	–	–	Not done	–	–	–
LIME15	GV	Iberian lynx	*Lynx pardinus*	Negative	–	–	–	–	–	–	–	–	–	–	–	–	–	–	–	–	–	–	Negative	Unspecific	–	–	–	–	–	–	–	Not done	–	–	–
LIME16	GV	Iberian lynx	*Lynx pardinus*	**Positive**	30.6	Negative	–	–	–	Negative	–	–	–	Negative	–	–	–	Negative	–	–	–	Unknown	Negative	–	–	–	–	–	–	–	–	Not done	–	–	–
LIME17	GV	Iberian lynx	*Lynx pardinus*	**Positive**	36.1	Not done	–	–	–	Not done	–	–	–	Not done	–	–	–	Not done	–	–	–	Unknown	Negative	–	–	–	–	–	–	–	–	Not done	–	–	–
LIME18	GV	Iberian lynx	*Lynx pardinus*	Negative	–	–	–	–	–	–	–	–	–	–	–	–	–	–	–	–	–	–	Negative	Unspecific	–	–	–	–	–	–	–	Not done	–	–	–
LIME19	GV	Iberian lynx	*Lynx pardinus*	Negative	–	–	–	–	–	–	–	–	–	–	–	–	–	–	–	–	–	–	Negative	Unspecific	–	–	–	–	–	–	–	Not done	–	–	–
LIME20	GV	Iberian lynx	*Lynx pardinus*	Negative	–	–	–	–	–	–	–	–	–	–	–	–	–	–	–	–	–	–	Negative	–	–	–	–	–	–	–	–	Not done	–	–	–
LIME21	GV	Iberian lynx	*Lynx pardinus*	Negative	–	–	–	–	–	–	–	–	–	–	–	–	–	–	–	–	–	–	Negative	–	–	–	–	–	–	–	–	Not done	–	–	–
LIME22	GV	Iberian lynx	*Lynx pardinus*	Negative	–	–	–	–	–	–	–	–	–	–	–	–	–	–	–	–	–	–	Negative	–	–	–	–	–	–	–	–	Not done	–	–	–
LIME23	GV	Iberian lynx	*Lynx pardinus*	Negative	–	–	–	–	–	–	–	–	–	–	–	–	–	–	–	–	–	–	Negative	*Doratomyces stemonitis*	–	–	–	–	–	–	–	Not done	–	–	–
LIME24	GV	Iberian lynx	*Lynx pardinus*	**Positive**	37.4	Negative	–	–	–	Not done	–	–	–	Not done	–	–	–	Not done	–	–	–	Unknown	Negative	*Doratomyces stemonitis*	–	–	–	–	–	–	–	Not done	–	–	–
LIME25	GV	Iberian lynx	*Lynx pardinus*	Negative	–	–	–	–	–	–	–	–	–	–	–	–	–	–	–	–	–	–	Negative	*Doratomyces stemonitis*	–	–	–	–	–	–	–	Not done	–	–	–
LIME26	GV	Iberian lynx	*Lynx pardinus*	**Positive**	37.5	Negative	–	–	–	Not done	–	–	–	Not done	–	–	–	Not done	–	–	–	Unknown	**Positive**	***C. felis***	C579T	Yes	–	–	–	–	***C. felis***	Not done	–	–	–
LIME27	GV	Iberian lynx	*Lynx pardinus*	Negative	–	–	–	–	–	–	–	–	–	–	–	–	–	–	–	–	–	–	Negative	Uncultured eukaryote	–	–	–	–	–	–	–	Not done	–	–	–
LIME28	GV	Iberian lynx	*Lynx pardinus*	Negative	–	–	–	–	–	–	–	–	–	–	–	–	–	–	–	–	–	–	Negative	Uncultured eukaryote	–	–	–	–	–	–	–	Not done	–	–	–
LIME29	GV	Iberian lynx	*Lynx pardinus*	Negative	–	–	–	–	–	–	–	–	–	–	–	–	–	–	–	–	–	–	Negative	*Meira nashicola*	–	–	–	–	–	–	–	Not done	–	–	–
LIME30	GV	Iberian lynx	*Lynx pardinus*	Negative	–	–	–	–	–	–	–	–	–	–	–	–	–	–	–	–	–	–	Negative	–	–	–	–	–	–	–	–	Not done	–	–	–
